# An integrated tumor, immune and microbiome atlas of colon cancer

**DOI:** 10.1038/s41591-023-02324-5

**Published:** 2023-05-19

**Authors:** Jessica Roelands, Peter J. K. Kuppen, Eiman I. Ahmed, Raghvendra Mall, Tariq Masoodi, Parul Singh, Gianni Monaco, Christophe Raynaud, Noel F.C.C. de Miranda, Luigi Ferraro, Tatiana C. Carneiro-Lobo, Najeeb Syed, Arun Rawat, Amany Awad, Julie Decock, William Mifsud, Lance D. Miller, Shimaa Sherif, Mahmoud G. Mohamed, Darawan Rinchai, Marc Van den Eynde, Rosalyn W. Sayaman, Elad Ziv, Francois Bertucci, Mahir Abdulla Petkar, Stephan Lorenz, Lisa Sara Mathew, Kun Wang, Selvasankar Murugesan, Damien Chaussabel, Alexander L. Vahrmeijer, Ena Wang, Anna Ceccarelli, Khalid A. Fakhro, Gabriele Zoppoli, Alberto Ballestrero, Rob A.E.M. Tollenaar, Francesco M. Marincola, Jérôme Galon, Souhaila Al Khodor, Michele Ceccarelli, Wouter Hendrickx, Davide Bedognetti

**Affiliations:** 1 Translational Medicine Division, Research Branch, Sidra Medicine, Doha, Qatar; 2 Department of Surgery, Leiden University Medical Center, Leiden, The Netherlands; 3 Department of Pathology, Leiden University Medical Center, Leiden, The Netherlands; 4 Department of Immunology, St. Jude Children’s Research Hospital, Memphis, TN USA; 5 Biotechnology Research Center, Technology Innovation Institute, Abu Dhabi, United Arab Emirates; 6 Institute for Transfusion Medicine and Gene Therapy, Medical Center-University of Freiburg, Freiburg, Germany; 7 Neuropathology, Medical Center-University of Freiburg, Freiburg, Germany; 8 BIOGEM Institute of Molecular Biology and Genetics, Ariano Irpino, Italy; 9 Department of Electrical Engineering and Information Technology (DIETI), University of Naples Federico II, Naples, Italy; 10 Integrated Genomics Services, Research Branch, Sidra Medicine, Doha, Qatar; 11 Translational Cancer and Immunity Center, Qatar Biomedical Research Institute (QBRI), Hamad Bin Khalifa University (HBKU), Qatar Foundation, Doha, Qatar; 12 College of Health and Life Sciences, Hamad Bin Khalifa University, Qatar Foundation, Doha, Qatar; 13 Department of Pathology, Sidra Medicine, Doha, Qatar; 14 Weill-Cornell Medicine Qatar, Doha, Qatar; 15 Department of Cancer Biology, Wake Forest School of Medicine, Winston-Salem, NC USA; 16 Women’s Wellness and Research Center, Hamad Medical Corporation, Doha, Qatar; 17 Department of Internal Medicine and Medical Specialties (DiMI), University of Genoa, Genoa, Italy; 18 Laboratory of Human Genetics of Infectious Diseases, The Rockefeller University, New York, NY USA; 19 Institut Roi Albert II, Cliniques Universitaires Saint-Luc, UCLouvain, Brussels, Belgium; 20 Department of Laboratory Medicine, Helen Diller Family Comprehensive Cancer Center, University of California, San Francisco, CA USA; 21 Department of Medicine, Institute for Human Genetics, Helen Diller Family Comprehensive Cancer Center, University of California, San Francisco, CA USA; 22 Laboratory of Predictive Oncology, Centre de Recherche en Cancérologie de Marseille, Institut Paoli-Calmettes, Aix-Marseille Université, Inserm UMR1068, CNRS UMR725, Marseille, France; 23 Department of Medical Oncology, Institut Paoli-Calmettes, Marseille, France; 24 Department of Laboratory Medicine and Pathology, Hamad Medical Corporation, Doha, Qatar; 25 Computational Sciences Department, The Jackson Laboratory, Farmington, CT USA; 26 Nurix Therapeutics, San Francisco, CA USA; 27 Medical Oncology, Fondazione Policlinico Universitario Agostino Gemelli IRCCS- Università Cattolica del Sacro Cuore, Rome, Italy; 28 IRCCS Ospedale Policlinico San Martino, Genoa, Italy; 29 Sonata Therapeutics, Watertown, MA USA; 30 Inserm, Laboratory of Integrative Cancer Immunology, Equipe Labellisée Ligue Contre Le Cancer, Centre de Recherche de Cordeliers, Université de Paris, Sorbonne Université, Paris, France; 31 Sylvester Comprehensive Cancer Center, Miller School of Medicine, University of Miami, Miami, FL, USA

**Keywords:** Colon cancer, Tumour immunology, Microbiome, T cells

## Abstract

The lack of multi-omics cancer datasets with extensive follow-up information hinders the identification of accurate biomarkers of clinical outcome. In this cohort study, we performed comprehensive genomic analyses on fresh-frozen samples from 348 patients affected by primary colon cancer, encompassing RNA, whole-exome, deep T cell receptor and 16S bacterial rRNA gene sequencing on tumor and matched healthy colon tissue, complemented with tumor whole-genome sequencing for further microbiome characterization. A type 1 helper T cell, cytotoxic, gene expression signature, called Immunologic Constant of Rejection, captured the presence of clonally expanded, tumor-enriched T cell clones and outperformed conventional prognostic molecular biomarkers, such as the consensus molecular subtype and the microsatellite instability classifications. Quantification of genetic immunoediting, defined as a lower number of neoantigens than expected, further refined its prognostic value. We identified a microbiome signature, driven by *Ruminococcus* *bromii*, associated with a favorable outcome. By combining microbiome signature and Immunologic Constant of Rejection, we developed and validated a composite score (mICRoScore), which identifies a group of patients with excellent survival probability. The publicly available multi-omics dataset provides a resource for better understanding colon cancer biology that could facilitate the discovery of personalized therapeutic approaches.

## Abstract (web summary)

A large, publicly available dataset integrating RNA, whole-exome, T cell receptor and 16S rRNA sequencing from patients with colon cancer enables the discovery of a prognostic score consisting of tumor, immune and microbial features.

## Main

Although there has been a substantial amount of research conducted on biomarkers for primary colon cancer, the current clinical guidelines in the USA and Europe (including the National Comprehensive Cancer Network and European Society for Medical Oncology guidelines) only rely on the tumor-node-metastasis staging and the detection of DNA mismatch repair (MMR) deficiency or microsatellite instability (MSI), in addition to standard clinicopathological variables, to determine treatment recommendations^1,2^. MSI is caused by somatic or germline defective of MMR genes and leads to the accumulation of somatic mutations, neoantigens resulting in immune recognition and high density of tumor infiltrating lymphocytes^3^.

The strength of the in situ adaptive immune reaction, as captured for instance by the evaluation of the density and spatial distribution of T cells (Immunoscore), is associated with a reduced risk of relapse and death independently of other clinicopathological variables, including MSI status^4,5^.

However, despite the overwhelming evidence of the prognostic effect of the Immunoscore and other immune-related parameters in colon cancer^6,7^, a lack of association between gene-expression-based estimates of immune response and patient survival in The Cancer Genome Atlas (TCGA) colon adenocarcinoma (COAD) cohort has been noted by the research community^8–10^. TCGA, for its genomic data richness and curation, represents the preeminent dataset for omics analyses; however, the collecting of comprehensive clinical data, including survival outcomes was neither a primary objective of TCGA nor a practical possibility in view of its worldwide scope and time constraints^11^. As such, the limited patient follow-up data associated with TCGA-COAD and other TCGA datasets has hindered statistically rigorous survival analyses^11^. In addition, TCGA did not include dedicated assays for T cell receptor (TCR) repertoire analysis or microbiome characterization, which was later performed using bulk DNA and RNA sequencing (RNA-seq) data and includes only few healthy solid tissue (for example healthy colon) samples^12,13^. Furthermore, as TCGA focused initially on cataloging genomic and molecular changes that occur in cancer cells, sample inclusion criteria based on stringent tumor purity cutoffs were imposed^14^, potentially biasing the population toward less-immune- or stroma-rich tumor specimens.

In recent years, while quantitative features of primary colon cancer, including those that are cancer cell intrinsic, immunological, stromal or microbial in nature, have been reported to be significantly associated with clinical outcomes, individually^15–17^, knowledge of how their interactions impact patient outcome is fragmentary.

To dissect this phenotypic complexity with respect to outcomes, we used orthogonal genomic platforms to rigorously profile a large collection of primary colon cancer specimens (unselected for tumor cell purity) and matched healthy colon tissue, complemented with curated clinical and pathological data annotation and appropriate follow-up.

## Results

### AC-ICAM overview

**Fig. 1:**
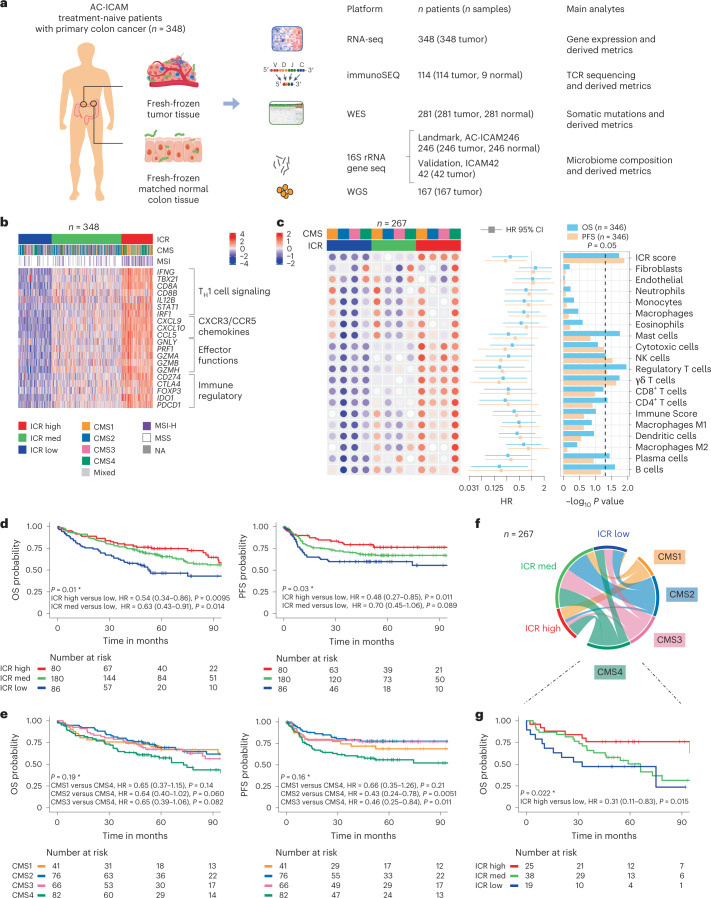
AC-ICAM study design, immune-related gene signatures, immune and molecular subtypes and survival. **a**, Samples from a total of 348 patients with colon cancer were included in AC-ICAM. Number of profiled samples and resulting analytes are indicated for each platform, including RNA-seq, WES, TCR sequencing (immunoSEQ TCRβ assay), 16S rRNA gene sequencing and metagenomic analysis from whole-genome sequencing (WGS) to profile microbiome composition. An additional 42 tumor samples were profiled with 16S rRNA gene sequencing that did not have any matched normal tissue available (ICAM42). **b**, Heat map of 20 ICR genes (normalized, log_2_-transformed expression values, *z* scored by row). Columns represent samples (*n* = 348) annotated with ICR cluster, CMS and MSI status. NA, not available. **c**, Deconvoluted abundancies of distinct infiltrating cell populations by ConsensusTME and their association with OS and PFS. Median enrichment scores (*z* scored by row) within each CMS, stratified by ICR cluster are indicated in the dotted heat map (left). HR (center) and corresponding 95% confidence intervals (error bars) as calculated by Cox proportional hazard regression are displayed as a forest plot (middle) (*n* = 346 independent samples from 346 patients). *P* values for the associated HRs are indicated in the bar chart (−log10 *P* value, right). **d**, Kaplan–Meier survival curves of ICR clusters for OS (left) and PFS (right). **e**, Kaplan–Meier survival curves of CMS for OS (left) and PFS (right). **f**, Circos plot of the relations between ICR and CMS classification. Size of each element is proportional to number of samples in each respective category. **g**, OS Kaplan–Meier curve of ICR clusters within the CMS4 subtype. (**d**,**e**,**g**) HRs and 95% confidence intervals are calculated by Cox proportional hazard regression. *Overall *P* value is calculated by log-rank test. Vertical lines indicate censor points. *P* values are two-sided.

**Extended Data Fig. 1:**
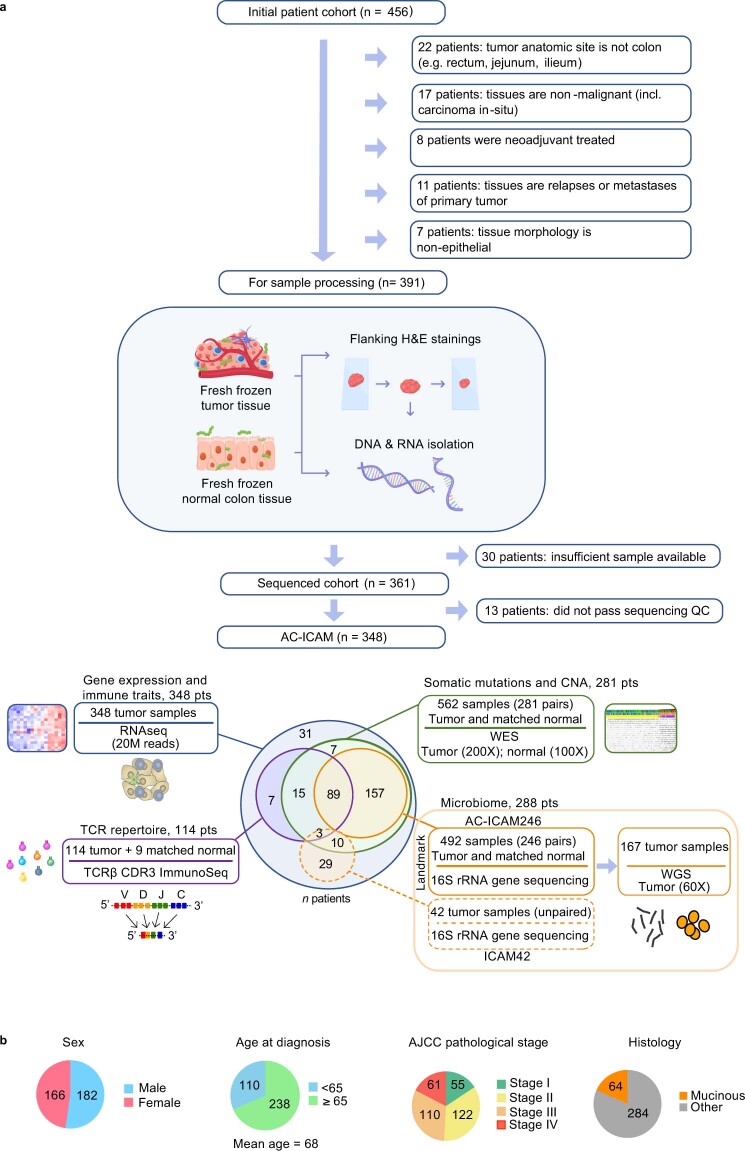
Study design for comprehensive genomic profiling of colon cancer. AC-ICAM study design. **a**, Visual representation of exclusion criteria and number of excluded samples from the 456 available samples in the LUMC biobank, followed by overview of tissue processing and genomic profiling of fresh-frozen tumor and matched normal colon tissue samples. Samples of a total of 348 colon cancer patients were included in AC-ICAM. Number of profiled samples and technical specifications are indicated for each platform, including RNA Sequencing (RNA-Seq), Whole-Exome Sequencing (WES), TCR sequencing (immunoSEQ TCRβ assay) and 16 S rRNA gene sequencing to profile the microbiome. AC-ICAM246 is a subset of AC -ICAM with tumor–normal matched rRNA 16 S microbiome data, while AC-ICAM42 only has tumor samples with 16 S rRNA gene sequencing. Venn diagram reflects overlap in number of patients between the different platforms applied. **b**, Summary of patient characteristics of colon cancer cohort (*n* = 348). Number in pie chart indicates number of patients in each category.

Fresh-frozen tumor samples and matched neighboring healthy colon tissues (tumor–normal pairs) from systemic treatment-naive, patients with histological diagnosis of colon carcinoma were profiled with orthogonal genomic platforms. After cross-platform quality control (based on whole-exome sequencing (WES) and RNA-seq data) and inclusion criteria checking, genomic data from 348 patients were retained and used for downstream analyses (Fig. 1a and Extended Data Fig. 1a,b; Methods provides further details). The median follow-up time was 4.6 years. We refer to this resource as the Sidra-LUMC AC-ICAM: an Atlas and Compass of Immune–Cancer–Microbiome interactions.

### The ICR outperforms conventional molecular classifications

A modular immune gene signature capturing the continuum of cancer immune surveillance, termed as the Immunologic Constant of Rejection (ICR)^18^, had been proposed^19^. We subsequently optimized and condensed it into a fixed 20-gene panel, showing prognostic significance in different cancer types (for example, melanoma^10^, bladder cancer^10^, breast cancer^20,21^, neuroblastoma^22^ and soft-tissue sarcoma^23^). The ICR also correlates with response to immunotherapy across multiple cancer types, including breast^24^, melanoma^10^ and non-small-cell lung cancer^25^. The ICR signature includes gene modules that reflect the activation of type 1 T (T_H_1) cell signaling, expression of CXCR3/CCR5 chemokine ligands, cytotoxicity and counter-activation of immunoregulatory mechanisms^21^ (Fig. 1b).

**Extended Data Fig. 2:**
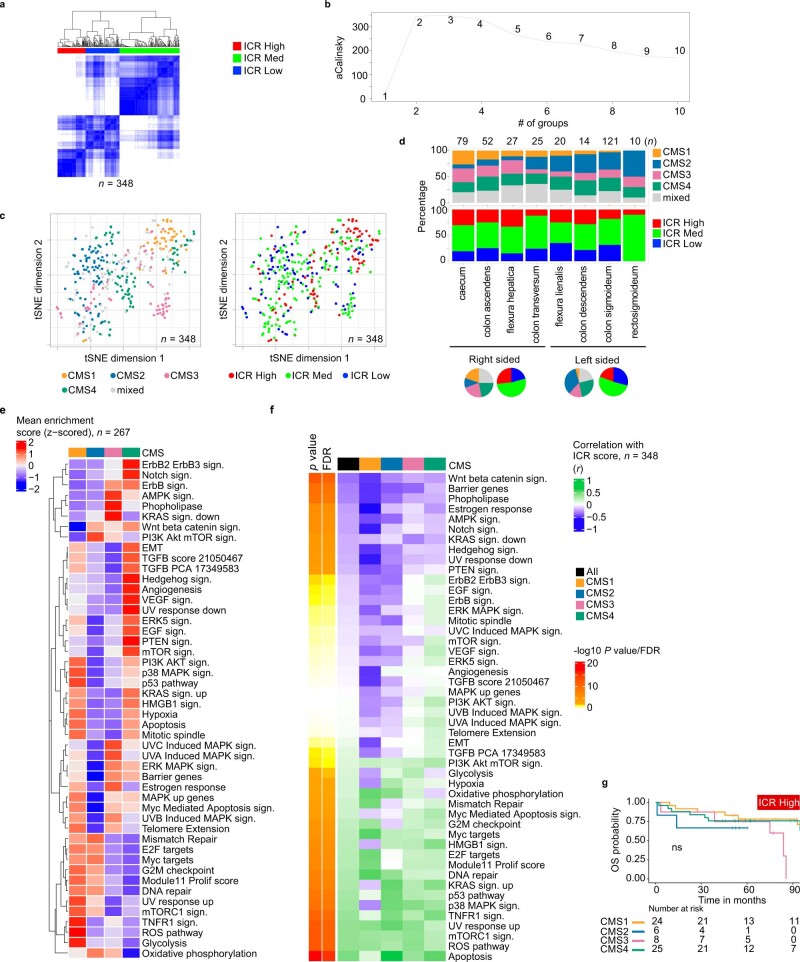
ICR clustering and oncogenic pathways. **a**, Visual representation of consensus clustering with a heat map of the 20 ICR genes, using 5000 repeats, agglomerative hierarchical clustering with ward criterion inner and complete outer linkage. **b**, The optimal number of clusters allowing for the best segregation of samples based on the Calinski-Harabasz criterion. **c**, First and second dimension from t-distributed stochastic neighbor embedding (t-SNE) dimension-reduction algorithm applied to whole-transcriptome data of colon tumor samples (*n* = 348) colored by CMS (left) and ICR cluster (right). **d**, Stacked bar chart showing proportion of CMS by anatomic location of the tumor. Pie charts reflect proportions within right sided (ceceum until colon transversum) and left sided tumors (flexura lienalis until rectosigmoideum). **e**, Mean Singe Sample Gene Set Enrichment Analysis (ssGSEA) score for oncogenic pathways by each CMS. **f**, Pearson correlation (two-sided) of oncogenic pathways with ICR score; EMT: epithelial to mesenchymal transition. **g**, Kaplan–Meier curves for Overall Survival (OS) of CMS subtypes within ICR High. Overall *P* value is calculated by log-rank test. All *P* values are two-sided; *n* reflects the independent number of samples in all panels.

**Extended Data Fig. 3:**
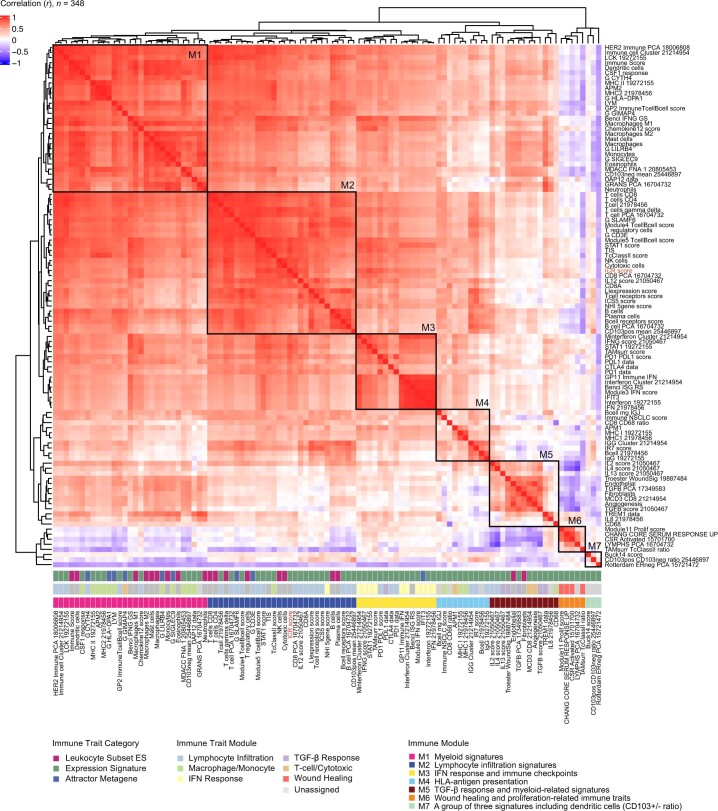
Immune Trait clustering and Module assignment. Pairwise Pearson correlation matrix of the 103 immune traits. Seven clusters of highly correlated immune traits were defined as Immune Modules: Myeloid signatures (M1), lymphocyte infiltration signatures (M2), IFN response and immune checkpoints (M3), HLA-antigen presentation signatures (M4), TGF-β response and myeloid signatures (M5), wound healing and proliferation-related immune traits (M6) and three separately clustered signatures (M7). Top annotation displays the Immune traits with their respective Immune module (M1-M7), Immune Trait Module and corresponding immune trait categories (as described in Sayaman et al, 2021).

As a first objective, we conducted a validation of the ICR signature on the AC-ICAM cohort. This objective was predefined before data were generated (prospective validation of retrospectively collected samples; Methods provides detail). A consensus-clustering approach based on the ICR genes (Extended Data Fig. 2a,b) segregated the cohort in three clusters/immune subtypes: ICR high (hot tumors), ICR medium and ICR low (cold tumors) (Fig. 1b). Systematic transcriptomic analysis using 103 previously defined immune traits (Methods) revealed co-clustering of these traits into seven different modules (M1–M7) (Extended Data Fig. 3), with ICR belonging to M2 (lymphocyte infiltration signature), together with other immune signatures, including the tumor inflammation signature^9^. We then characterized the immune disposition in relation to Consensus Molecular Subtypes (CMS)^16^, a well-defined transcriptomic-based classification of colon cancers. CMS categories include CMS1/immune, CMS2/canonical, CMS3/metabolic and CMS4/mesenchymal. Overall, *t*-distributed stochastic neighbor embedding (*t*-SNE) plotting of the whole expression data segregated CMS1–CMS3 samples, but a high heterogeneity was observed for CMS4 (Extended Data Fig. 2c, left). Within CMS subtypes, ICR varied considerably (Extended Data Fig. 2c, right). While most of the CMS1 samples were ICR high, implying immune activation^26^, CMS4 samples were spread across the three ICR immune subtypes. According to the anatomic location, a progressive right-to-left colon enrichment (for CMS2) and depletion (for CMS1) (Extended Data Fig. 2d), was evident^16^. ICR score (average of the 20 ICR genes) and leukocyte subsets enrichment scores, showed only a modest decrease from right-to-left colon, with ICR high being more prevalent in cecum versus rectosigmoid tumors (Supplementary Fig. 1). The enrichment scores of cancer-cell-related pathways^10^ were clearly distinct across CMS subtypes (Extended Data Fig. 2e). ICR score correlated negatively with certain cancer-cell pathways in all CMS subtypes (for example, WNT-β catenin and NOTCH signaling), whereas a positive correlation with immunosuppressive and stromal-related pathways (for example, transforming growth factor (TGF)-β, epithelial to mesenchymal transition and vascular endothelial growth factor signaling) was only observed in CMS4 tumors (Extended Data Fig. 2f).

The abundance of natural killer (NK) cell and T cell subsets was the highest in the ICR-high immune subtype across all CMS, whereas other leukocyte subsets were more variable (Fig. 1c, heat map). Conversely, the abundance of fibroblast and endothelial cells was increased in CMS4, irrespective of ICR assignment, confirming the increased stromal content in these tumors. Based on statistical significance, the association between ICR score and progression-free survival (PFS) was stronger than what observed for any stromal cell or leukocyte subsets; similar results were obtained for the association with overall survival (OS) (Fig. 1c, forest plot).

ICR immune subtypes had distinct OS and PFS, which gradually increased from ICR low to high (Fig. 1d). As expected, CMS4 was associated with poor survival^16^; however, ICR reverted this negative trend in survival, with ICR high being associated with longer OS even within the CMS4 group (Fig. 1e–g). Conversely, CMS did not stratify the ICR-high cluster (Extended Data Fig. 2g). ICR remained significantly associated with improved OS in the Cox multivariate analysis (together with pathological stage and age), whereas microsatellite instability (MSI) status and CMS did not (Supplementary Table 2). The relationships between ICR and CMS depicted in Fig. 1 were confirmed in the TCGA colon cancer cohort (TCGA-COAD; Supplementary Fig. 2). Overall, in TCGA, the survival differences were attenuated (in the PFS analysis) or absent (in the OS analysis) for ICR, immune infiltrates and CMS. Nevertheless, ICR still stratified survival in patients with CMS4 cancers (Supplementary Fig. 2; PFS analysis). Overall, we validated the prognostic role of ICR in colon cancer.

### ICR captures tumor-enriched, clonally expanded T cells

It has been reported that only a minority of T cells infiltrating a tumor tissue is specific for tumor antigens (less than 10%)^27–29^. Most intratumoral T cells are therefore referred to as bystander T cells. We then sought to address why ICR, which measures T cell infiltration and functional orientation without considering antitumor specificity, bears such a strong prognostic connotation.

**Fig. 2:**
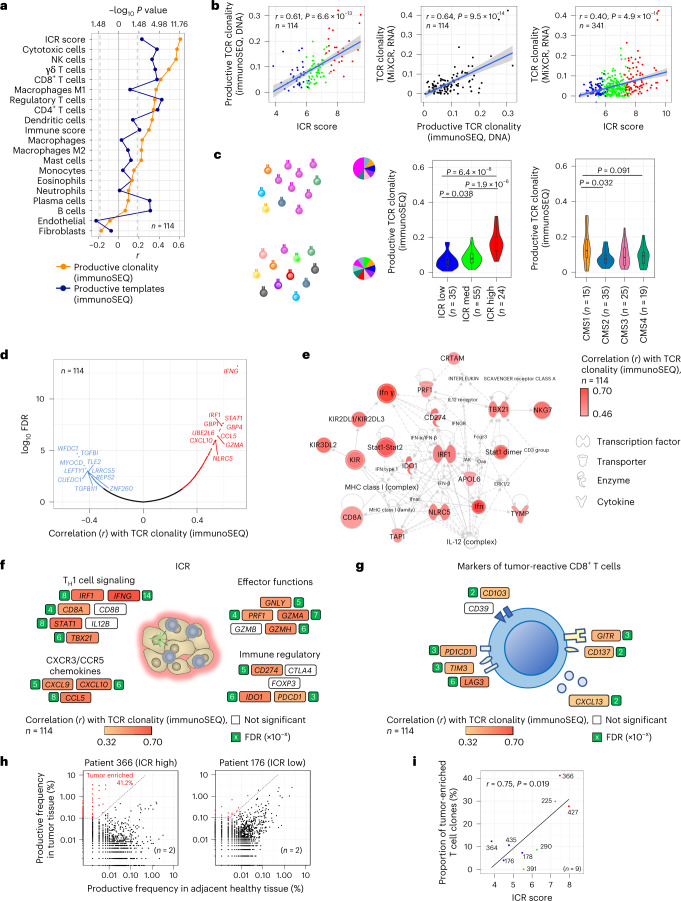
TCR metrics and correlation with immune-related genes, immune and molecular subtypes. **a**, Correlation between immune gene signatures and TCR metrics from immunoSEQ DNA sequencing. **b**, Scatter-plots visualizing correlation between ICR score, productive TCR clonality by immunoSEQ DNA sequencing and TCR clonality as determined by MiXCR using RNA-seq data. Pearson’s *r* and *P* value of the correlations are indicated. The gray area reflects the 95% confidence level interval for predictions of the linear regression model. **c**, Visualization of a T cell repertoire with a high clonality (top) and low clonality (bottom). Each color represents a unique T cell clone, proportions are represented as illustrative circle diagrams. Violin plots show the relationship between productive TCR clonality and ICR classification and CMS subtypes, center line, box limits and whiskers represent the median, interquartile range and 1.5× interquartile range. *P* values were calculated using a two-sided, unpaired Student’s *t*-test. **d**, Pearson correlation between all genes (*n* = 18,270) and TCR clonality (colored, FDR < 0.05). Top ten genes with highest positive correlation and top ten genes with highest inverse correlation are labeled. FDR calculated by Benjamini–Hochberg correction. **e**, Core network of genes with the highest association with productive TCR clonality (top 50 genes) using Ingenuity Pathway Analysis. **f**,**g**, Pearson’s correlation between immunoSEQ-based TCR productive clonality and the expression of ICR genes (**f**) and genes that express markers of tumor-reactive CD8^+^ T cells (**g**). The magnitude of significance for each correlation is represented by the number in the green square indicating the exponent (x) in the scientific notation of the FDR (x10^-x^). **h**, Example scatter-plots for an ICR-high sample and an ICR-low sample showing overlap between clones from the primary tumor and its matching healthy colon tissue sample. Tumor-enriched T cell clones (>0.1% in the tumor, which are at least 32 times higher in the tumor compared to normal) are highlighted. **i**, Correlation of proportion of tumor-enriched T cell clones in the tumor (in percent) with ICR score. Pearson’s *r* and *P* value of the correlation are indicated in the plot. All *P* values are two-sided.

**Extended Data Fig. 4:**
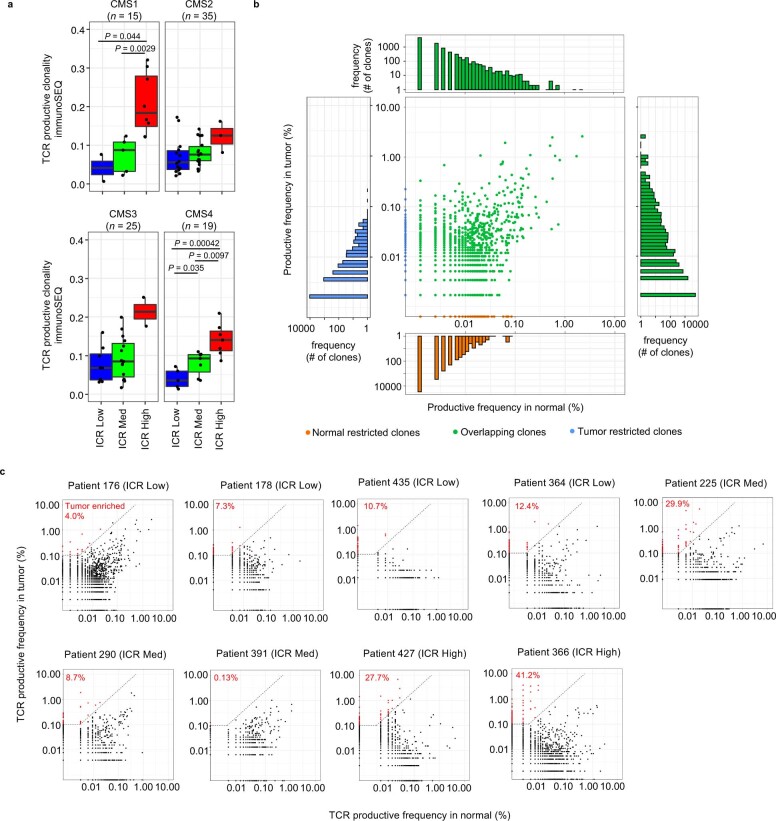
TCR productive clonality by CMS and comparison of clonotypes in paired tumor and normal colon tissue. **a**, Box-plot of TCR clonality for each ICR, stratified by CMS. *P* values are calculated using unpaired, two-sided t-test. Center line, box limits and whiskers represent the median, interquartile range and 1.5x interquartile range respectively. *n* reflects independent number of samples. **b**, Example of a plot showing productive TCR frequencies in normal tissue (x-axis) and corresponding frequency in tumor tissue (y-axis). Blue indicates T cell clones that are restricted to the tumor, while T cell clones that are unique to normal colon tissue are orange. Represented in green are T cell clones found both in tumor and normal colon tissue. The side panels represent a cumulative histogram of the TCR productive frequencies across that axis. **c**, Productive TCR scatter-plots of all nine patients for which TCR sequencing was performed on both tumor and matched normal colon samples. T cell clones in the upper left region (red) are considered significantly enriched in the tumor. Tumor-enriched clones were defined as T cell clones with an abundance of >0.1% in the tumor, that are at least 32 times more abundant in the tumor compared to the normal.

A dedicated deep sequencing of the *TRB* gene by immunoSEQ was performed on all samples (114 tumors and 9 healthy colon tissues) with sufficient DNA for this assay. *TRB* gene sequence information was also extracted from bulk RNA-seq using MiXCR (*n* = 341)^30^. Among stromal cell and leukocyte subsets (measured by RNA-seq), the strongest correlation with the number of conventional (αβ) T cells with a productive TCR (immunoSEQ TCR productive DNA templates), was observed for estimates of T cell subsets (Fig. 2a), implying robustness of DNA and RNA-based measurements; however, the strongest correlation with immunoSEQ TCR productive clonality was observed for ICR score (*r* = 0.61), substantiating the ability of ICR to capture additional features beyond T cell abundance (Fig. 2a,b). Despite the inherent limitation in terms of sensitivity and specificity of TCR repertoire analysis using bulk RNA-seq, MiXCR TCR clonality correlated well with immunoSEQ TCR clonality (*r* = 0.64) as well as with ICR (*r* = 0.40) (Fig. 2b). Consistently, among ICR clusters (overall and within CMS categories), the immunoSEQ TCR clonality was the highest in the ICR-high group and in the CMS1/immune group among CMS subtypes (Fig. 2c and Extended Data Fig. 4a), which has the highest proportion of ICR-high tumors (Fig. 1f). Using the whole transcriptome (18,270 genes), six out of the top ten genes positively correlating with TCR immunoSEQ clonality were represented by ICR genes (*IFNG*, *STAT1*, *IRF1*, *CCL5*, *GZMA* and *CXCL10*) (Fig. 2d). Furthermore, the network of the top 50 genes correlating with immunoSEQ TCR clonality were centered on the ICR master regulators *IRF1* and *STAT1* (Fig. 2e). The correlation of immunoSEQ TCR clonality with most of the ICR genes was stronger compared to the one observed with markers of tumor-reactive CD8^+^ T cells defined by single-cell sequencing approaches^31^ (Fig. 2f,g).

For nine patients, immunoSEQ TCR profiles were available on both the tumor and matched healthy colon tissue. This allowed the definition of overlap between T cell clones observed in the tumor and healthy colon sample for each of these patients (Extended Data Fig. 4b,c). The proportion of tumor-enriched T cell clones correlated with ICR score (*r* = 0.75, *P* = 0.019; Fig. 2h,i). This implies that the T cell clones infiltrating ICR-high tumors are highly divergent from those infiltrating healthy tissue, whereas T cells in ICR-low tumors are also present in healthy tissue.

In conclusion, our analyses demonstrated that the ICR signature captures the presence of tumor-enriched, clonally expanded T cells, possibly explaining its prognostic connotation.

### Somatic alterations associated with weak immune response

**Extended Data Fig. 5:**
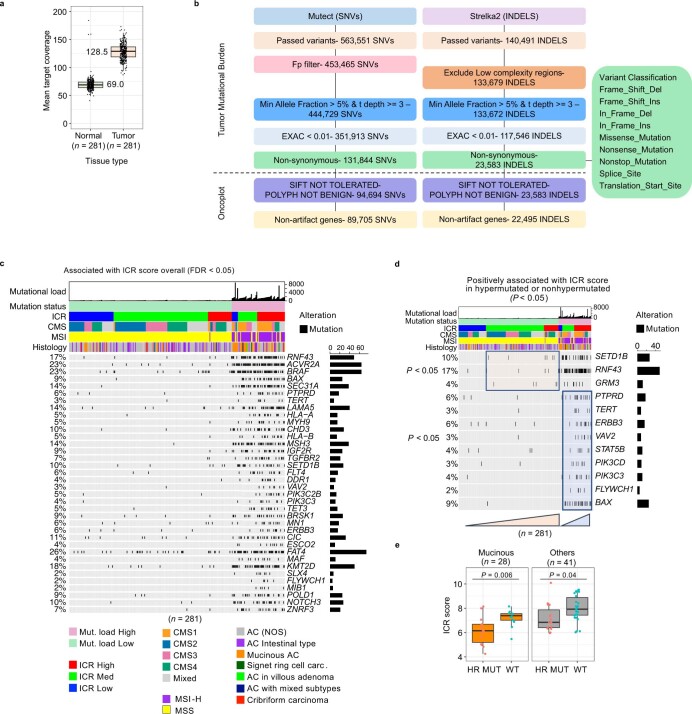
Mutation calling and associations between ICR and somatic alterations. **a**, The mean target coverage in tumor and normal colon tissue. Center line, box limits and whiskers represent the median, interquartile range and 1.5x interquartile range respectively. **b**, Visual representation of the mutect-strelka2 based mutation calling pipeline, indicating at each step the remaining SNV and INDELS in the AC-ICAM cohort. **c**, Oncoprint of genes with the highest correlation with ICR score according to fitting of a binomial linear regression model using all samples as input. **d**, Oncoprint of genes with the highest correlation with ICR score according to fitting of binomial linear regression models using either hypermutated or non-hypermutated samples. All genes with *P* value < 0.05 as predictor variable in the regression model are displayed (**c**, **d**). **e**, Box-plot of ICR score in samples with a mutation in any HR repair gene (*BRCA1*, *BRCA2*, *FANCA*) versus those without mutation or wild-type (WT), stratified by tumor histology (mucinous versus all other histologies) in hypermutated samples. *P* value is calculated using unpaired, t-test. Center line, box limits, and whiskers represent the median, interquartile range and 1.5x interquartile range respectively. All *P* values are two-sided; *n* reflects the independent number of samples in all panels.

We sought to identify potential drivers of immune responsiveness related to cancer cell somatic alterations, such as mutations and copy-number variations by performing WES (Extended Data Fig. 5a,b) on 281 tumor samples and corresponding healthy tissue.

**Fig. 3:**
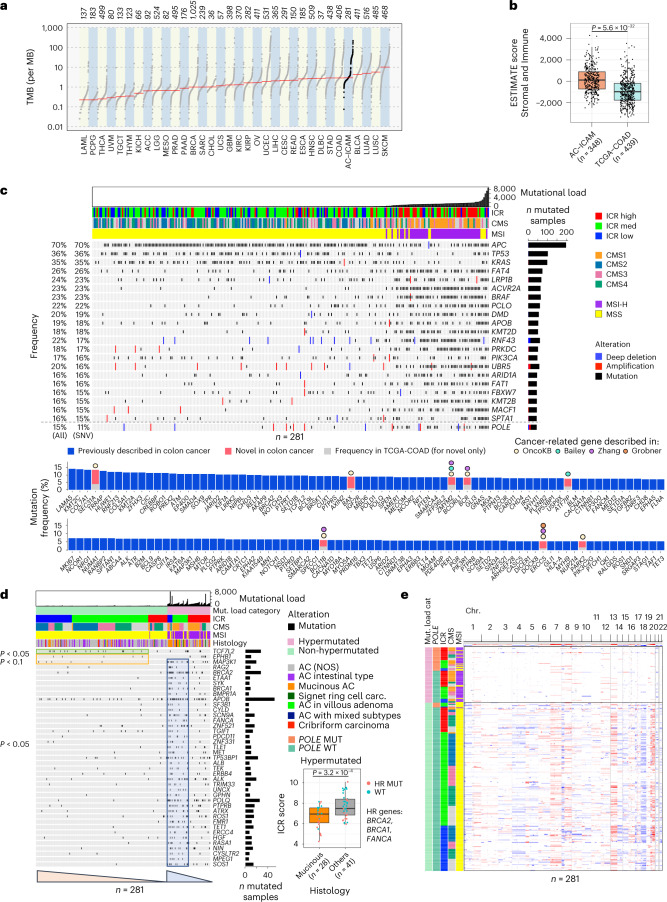
Detection of somatic alterations and association with tumor immune subtypes. **a**, TMB in the AC-ICAM cohort and all TCGA cohorts. **b**, ESTIMATE scores in AC-ICAM and TCGA-COAD cohorts. Unpaired two-sided Student’s *t*-test. **c**, Oncoprint of cancer-related genes that are most frequently somatically altered. Samples are ordered by nonsynonymous mutational load. Frequency of mutated samples as percentage of the total number of samples is shown on the left side of the plot, including the percentage of all somatic alterations, including deep deletions, amplifications and single-nucleotide variants (SNVs) and for only SNVs. Genes are ordered by frequency of SNVs. Genes with an SNV frequency ≥15% are included in the oncoprint, whereas genes with a frequency between 5–15% are included in the bar chart below. *POLE* is included below the dotted gray line in the oncoprint to visualize the *POLE* mutation in relation to MSI status. **d**, Oncoprint of genes with somatic mutations that are associated with low ICR score as determined by fitting of binomial linear regression models. Binomial linear models were generated for non-hypermutated and hypermutated subgroups separately. All genes with *P* value < 0.05 as predictor variable in the regression model are displayed. Orange triangle marks genes that were associated with lower ICR score in non-hypermutated samples, whereas the blue triangle highlights genes associated with low ICR in hypermutated samples. Significance of the association is indicated on the left of the plot. Box-plot of ICR score by tumor histology (mucinous versus all other histological classifications) in hypermutated samples, mutated in either of the homologous recombination (HR) repair genes (*BRCA1*, *BRCA2* and *FANCA*) are indicated by the color of the dots. *P* value is calculated using unpaired, two-sided Student’s *t*-test. AC, adenocarcinoma; NOS, not otherwise specified; MUT, mutant; WT, wild-type. **e**, Heat map of copy-number changes of the 22 autosomes, with red indicating gains and blue indicating losses. Samples are sorted by mutational load category, *POLE* mutation status, ICR, CMS and MSI, consecutively. All *P* values are two-sided; *n* reflects the independent number of samples. For all box-plots, center line, box limits and whiskers represent the median, interquartile range and 1.5× interquartile range, respectively.

In terms of somatic mutations, the tumor mutational burden (TMB) of the AC-ICAM dataset was highly comparable to the TCGA-COAD cohort (Fig. 3a), as were the clinicopathological parameters (Supplementary Fig. 3). Unlike the TCGA-COAD cohort, however, inclusion of samples in our study did not depend on tumor purity. In fact, stromal and immune content (ESTIMATE score) and the infiltration of individual lymphocyte subpopulations (Fig. 3b and Supplementary Fig. 4) was significantly increased in the AC-ICAM compared to the TCGA-COAD datasets, whereas the opposite was observed for cancer-cell-intrinsic signatures (Supplementary Fig. 5). This was paralleled by a lower proportion of CMS1 and a higher proportion of ICR low in the TCGA-COAD compared to AC-ICAM (Supplementary Fig. 6). While the same proportion of MSI-high (MSI-H) cases was observed in the two cohorts (Supplementary Fig. 3), MSI-H TCGA-COAD samples displayed lower levels of CD8^+^ T cells (Supplementary Fig. 6), which is consistent with a positive selection of less-immune-infiltrated specimens. We then subsampled the cohort 100 times using two methodologies: one was random and the other was on a subgroup of samples with an ESTIMATE distribution that approximates that of the TCGA-COAD. The random subsampling resulted in tripling the number of subsets in which the Cox proportional regression showed a statistically significant survival benefit of the ICR score compared to the sampling method approximating the TCGA-COAD ESTIMATE distribution (*P* < 0.0001, chi-squared test) (Supplementary Figs. 7 and 8). These findings suggest that a lower immune-stroma infiltration could have an impact on survival analysis, contributing to the lack of correlation between immune traits and OS observed in TCGA-COAD (Supplementary Fig. 2).

An overview of the somatic alterations landscape of the AC-ICAM cohort is represented in Fig. 3c. We identified eight cancer-related genes^32–35^ with a mutation frequency of <5% in TCGA-COAD^36^ and Nurses’ Health Study (NHS)-Health Professionals Follow-up Study (HPFS) cohorts^37^ that were enriched in AC-ICAM and that had not been previously reported as colon cancer oncogenic mediators^38^ or cancer driver genes for colorectal cancer^32^ (highlighted in pink in Fig. 3c).

Overall, we observed somatic mutations in 42 genes associated positively (*P* < 0.05) with ICR score, whereas no mutations were enriched in samples with a lower ICR score (Extended Data Fig. 5c). When we stratified the analysis according to the hypermutation status, we identified gene mutation frequencies that were associated with both a higher (Extended Data Fig. 5d) or lower ICR score (Fig. 3d, orange and green squares). Mutations of *MAP3K1*, which were previously associated with low ICR in breast and pan-cancer TCGA analysis^10,21^, were the only ones with a negative correlation with ICR score in both hypermutated and non-hypermutated cancers in AC-ICAM. In hypermutated tumors, mutations in the homologous recombination repair genes *BRCA1*, *BRCA2* and *FANCA* and the mucinous histology were associated with a lower ICR score, consistently with the previously reported enrichment of *BRCA1* and *BRCA2* somatic mutations in mucinous colorectal tumors^39^ (Fig. 3d, box-plot and Extended Data Fig. 5e).

With respect to somatic copy-number genomic aberrations (SCNAs), no clear association was observed with ICR immune classification as they were dependent primarily on the mutational load/MSI status and secondarily on the CMS status^16,40^ (Fig. 3e).

Altogether, this analysis identified a relationship between specific cancer-related genes and/or histological characteristics and a lower level of intratumoral immune activation.

### Genetic immune editing refines the prognostic value of ICR

**Fig. 4:**
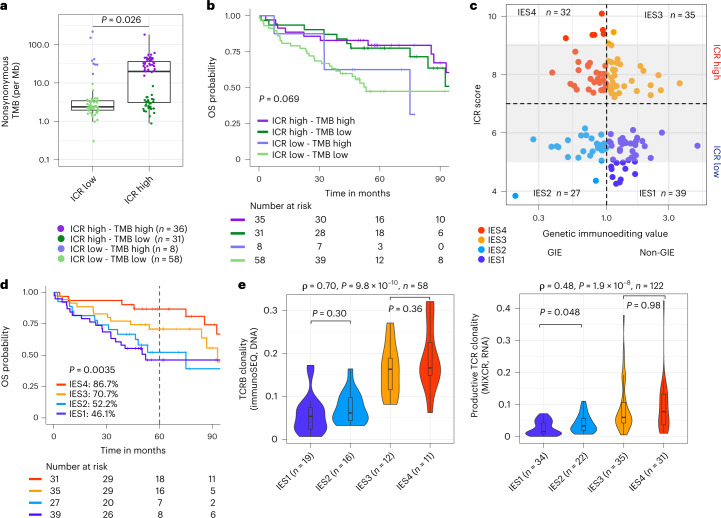
Tumor mutational burden, immunoediting score, TCR clonality and survival. **a**, Nonsynonymous mutation frequency per mega base (Mb) by ICR cluster. *P* value was calculated using unpaired, two-sided Student’s *t*-test. Center line, box limits and whiskers represent the median, interquartile range and 1.5× interquartile range, respectively. **b**, Kaplan–Meier OS curve for the combination of ICR cluster and mutational load category. Mutational load high is defined as nonsynonymous mutation frequency of >12 per Mb. Overall *P* value is calculated by log-rank test. **c**, Scatter-plot of ICR score by genetic immunoediting (GIE) value for ICR-high and ICR-low samples. Number of samples in each quadrant is indicated in the graph. Gray area delineates ICR scores from 5–9. **d**, Kaplan–Meier for OS by IES. Censor points are indicated by vertical lines and corresponding table of number of patients at risk in each group is included below the Kaplan–Meier plot. Overall *P* value is calculated by log-rank test. **e**, Violin plot of IES by productive TCR clonality (immunoSEQ) (left) and MiXCR-derived TCR clonality (right). Spearman correlation statistics are indicated above each plot. Significance within ICR low and high is indicated. Center line, box limits and whiskers represent the median, interquartile range and 1.5× interquartile range, respectively. *P* values are two-sided, *n* reflects the independent number of samples.

**Extended Data Fig. 6:**
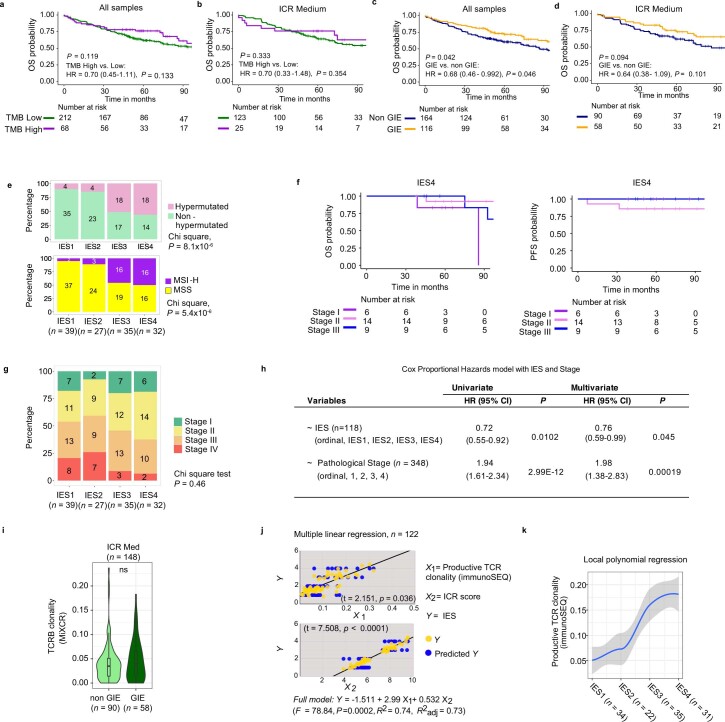
Immunoediting score and survival analysis. **a**, Kaplan–Meier curves for OS for patients with a tumor with High (>12/Mb) versus Low (<12/Mb) TMB. **b**, Same as **a**, but only including ICR Medium. **c**, Kaplan–Meier curves for OS by GIE status. **d**, Same as **c** in ICR Medium patients. Overall *P* value is calculated by log-rank test and *P* value corresponding to HR is calculated using cox proportional hazard regression (**a**-**d**). **e**, Stacked bar charts of mutational load category (top) and MSI status (bottom) per IES. **f**, Kaplan–Meier curves for OS (left) and PFS (right) stratified by AJCC pathological stage (I, II, III) within IES4. Stratification was not performed for stage IV due to the limited number (*n* = 2). **g**, Stacked bar chart of distribution of AJCC Pathological Tumor Stage by IES. **h**, Multivariate cox proportional hazards model for OS including IES (ordinal, IES1, IES2, IES3, IES4) and AJCC Pathological Tumor Stage (ordinal, Stage I, II, III, IV). *P* values corresponding to HR calculated by cox proportional hazard regression analysis are indicated. **i**, Violin plot represents TCR clonality as determined by MiXCR in ICR Medium samples. Center line, box limits, and whiskers represent the median, interquartile range and 1.5x interquartile range respectively. *P* value calculated by unpaired, two-sided t-test. **j**, Results of the multiple linear regression model showing the respective contributions of productive TCR clonality (*X*_*1*_) and (*X*_*2*_) for prediction of IES (*Y*). Corresponding significance of the effects are indicated in the scatter-plots (left). **k**, Local Polynomial Regression Fitting of productive TCR clonality by IES (ordinal variable). The gray band reflects the 95% confidence interval for predictions of the local polynomial regression model. All *P* values are two-sided; *n* reflects the independent number of samples in all panels. Overall Survival (OS). Tumor Mutational Burden (TMB). Genetic Immunoediting (GIE). ImmunoEditing Score (IES).

We then proceeded by integrating ICR and TMB data. While hypermutated samples frequently displayed an ICR-high phenotype, a considerable proportion of ICR-high samples (46%) had a low TMB (Fig. 4a), which did not impact the OS within or across ICR classes (Fig. 4b and Extended Data Fig. 6a,b), coherently with what previously observed for the Immunoscore^4,5^.

While we observed no difference in OS between high versus low TMB (Extended Data Fig. 6a) tumors, the presence of genetic immunoediting (GIE; calculated as the ratio of the observed versus the expected number of neoantigens) was nevertheless associated with improved OS (Extended Data Fig. 6c). We then explored a composite score, called the immunoediting score (IES), based on both ICR cluster assignment and presence or absence of GIE (IES1 = ICR low and no GIE; IES2 = ICR low and GIE; IES3 = ICR high and no GIE; IES4 = ICR high and GIE) (Fig. 4c), similar to what was proposed in metastatic colon cancer by combining the Immunoscore and GIE^41^. We propose that the combination of the two parameters may more accurately reflect the presence of an active, antitumor immune response. Consistently with this hypothesis, a progressive increase of OS was observed from IES1 to IES4 (Fig. 4d). The additive value of combining ICR with GIE was confirmed in ICR-medium samples (Extended Data Fig. 6d), which served here as an internal validation. While the TMB was higher in GIE versus non-GIE samples, GIE was observed in a significant proportion of both hypermutated and non-hypermutated tumors (55.1 versus 38.7%) (Supplementary Fig. 9). Patients with IES4 tumors, of which ∼50% were hypermutated or MSI-H (Extended Data Fig. 6e), indeed demonstrated improved survival, with similar survival across stage I–III (Extended Data Fig. 6f). No conclusion could be made in the IES4 stage IV subgroup as it only included two patients. No statistically significant difference was observed in terms of stage distributions and IES (chi-squared test, *P* = 0.46; Extended Data Fig. 6g). IES remained significantly associated with OS in a multivariable Cox model corrected by stage (*P* = 0.045; Extended Data Fig. 6h). IES categories also differed in term of TCR clonality, with increasing clonality from IES1 to IES4 (Fig. 4e). The same trend was observed within the ICR-medium subgroup, in which the TCR clonality was increased (although not significantly) in the GIE samples compared to the non-GIE samples (Extended Data Fig. 6i). The positive correlation between IES and TCR clonality was statistically significant when corrected for ICR score using multiple regression analysis and was confirmed by local polynomial regression analysis (Extended Data Fig. 6j,k). Overall, these results suggest that the level of immune editing (IES) accurately reflects the level of a protective antitumor immune response driven by clonally expanded T cells.

### Microbiome composition in healthy and colon cancer tissue

**Fig. 5:**
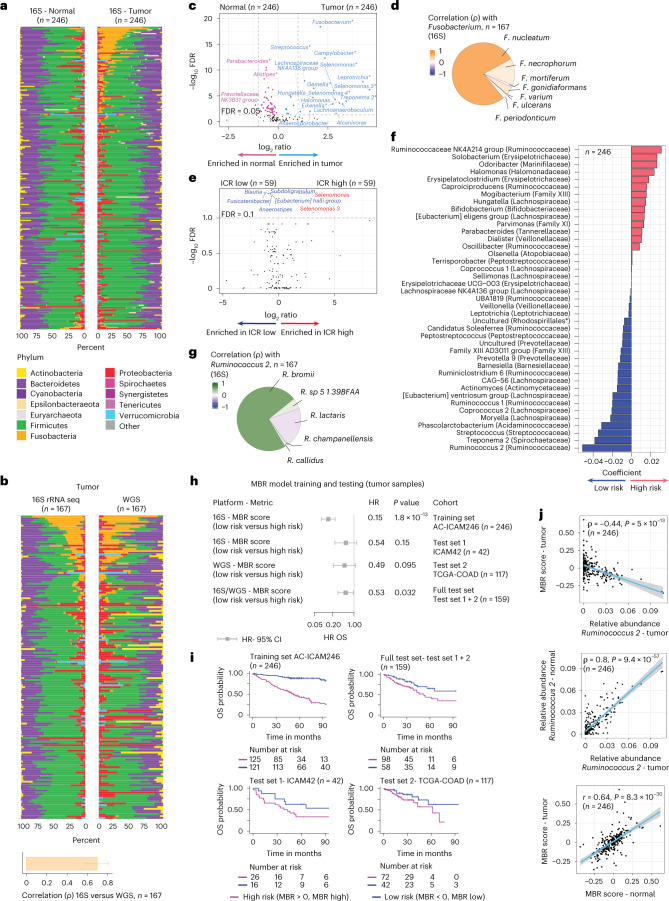
Microbiome in tumor and healthy tissue and relationship with ICR and survival. **a**, Microbiome composition at phylum level using 16S rRNA gene-sequencing estimates in tumor and matched healthy colon tissue; samples are ordered by difference in *Fusobacteria* between tumor and healthy tissue. **b**, Side-by-side microbiome composition at the phylum level using 16S rRNA gene sequencing and WGS estimates in colon cancer tissue. Bar plot shows mean of Spearman correlation between the two techniques for each phylum, error bar represents s.d. **c**, Differences between tumor and matched healthy colon genera (paired Mann–Whitney *U*-test). *Previously described associations (Supplementary Table 5). **d**, Pie chart reflects the contribution of each individual species to the total *Fusobacterium* sp. as determined by WGS data; color gradient reflects the Spearman correlation between the relative abundance of individual species derived from WGS and the relative abundance of *Fusobacterium* determined by 16S rRNA gene sequencing. **e**, Differences of microbiome genera between ICR high and ICR low tumor samples (unpaired Mann–Whitney *U*-test). **f**, The coefficients of the 41 taxa in the MBR classifier as selected by the OS elastic-net Cox regression model. Family is indicated between parentheses. *The taxonomical order is indicated between brackets, as family was unassigned (uncultured). **g**, Pie chart as in **d** but for *Ruminococcus* sp. **h**, Forest plot showing the HR (center), 95% confidence intervals (error bars) and corresponding *P* value calculated by Cox proportional hazard regression analysis for OS of the 16S MBR classifier scores in training and test sets. **i**, Kaplan–Meier curves corresponding to **h**. **j**, Correlation between MBR score in the tumor versus relative abundance of *Ruminococcus 2* (top), relative abundance of *Ruminococcus 2* in healthy tissue versus tumor (middle) and MBR score in tumor versus healthy colon (bottom). The gray band reflects the 95% confidence interval for predictions of the linear regression model between the plotted variables. *P* value for Spearman correlation for relative abundance and *P* value for Pearson correlation for MBR scores are indicated. OS. All *P* values are two-sided; *n* reflects the independent number of samples.

We sequenced the 16S rRNA gene using DNA extracted from matched tumor and healthy colon tissues from 246 patients (Fig. 5a; AC-ICAM246 cohort). This dataset was used for the microbiome landmark analysis. Whole-genome sequencing (WGS, median coverage 76×) was performed in a subgroup of these samples (*n* = 167; Fig. 5b) for technical validation. For validation purposes, once the landmark analysis was completed, we analyzed 16S rRNA gene-sequencing data from 42 additional tumor samples for which no matched normal DNA was available for this assay (referred here as ICAM42 cohort, see also Fig.1a).

After applying the same abundance filter to AC-ICAM246 and TCGA-COAD datasets, AC-ICAM captured all the genera detected in TCGA-COAD^13^, which displayed almost identical co-correlation patterns in the two cohorts, in additional to several other genera (Supplementary Fig. 10).

**Extended Data Fig. 7:**
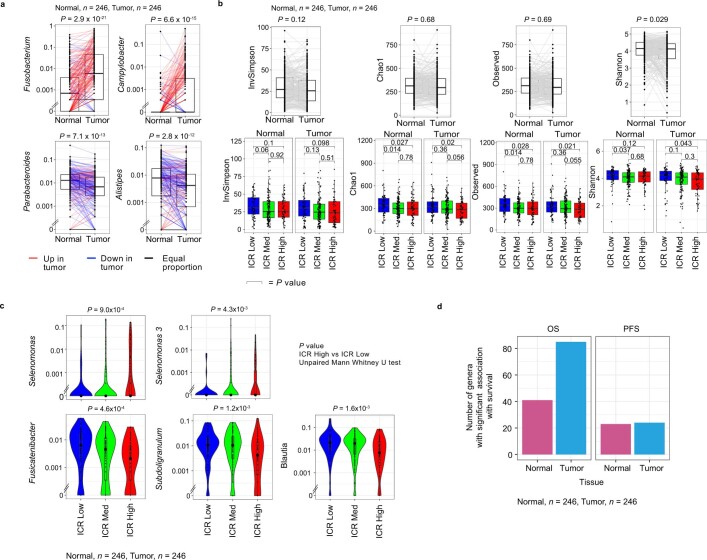
Microbiome in tumor and normal tissue. **a**, Paired box-plot for microbial genera that were most significantly enriched in tumors compared to matched normal, and vice versa. Tumor and normal pairs are connected, red lines reflect pairs with an increased proportion in the tumor, while blue lines reflect a decreased proportion in the tumor compared to matched normal. Center line, box limits, and whiskers represent the median, interquartile range and 1.5x interquartile range respectively. **b**, Distinct alpha diversity matrices (InvSimpson, Chao1, Observed and Shannon) between tumor and normal colon tissue, *P* values were calculated using paired Mann–Whitney U-test (upper). Alpha diversity between ICR High, ICR Medium, and ICR Low, in normal (N) and tumor (T) tissues (lower), *P* values were calculated using an unpaired Mann–Whitney U-test. Center line, box limits, and whiskers represent the median, interquartile range and 1.5x interquartile range respectively. **c**, Violin plots of relative abundance of microbial genera stratified by ICR cluster (top two most significantly enriched in ICR High, and top three in ICR Low). Center line, box limits, and whiskers represent the median, interquartile range and 1.5x interquartile range respectively. ICR High, *n* = 59; ICR Medium, *n* = 128; ICR Low, *n* = 59. **d**, Number of genera significantly associated with OS and PFS using either the tumor or normal tissue. All *P* values are two-sided. *n* reflects the independent number of samples in all panels. Overall Survival (OS). Progression-Free Survival (PFS).

First, we compared the relative abundance of taxa between matched tumor and healthy colon tissues. At the phylum level, we observed a significant increase of Fusobacteria in tumor compared to healthy samples (Fig. 5a) with a high concordance between the two methods (Fig. 5b). At the genus level, as expected^42^, the strongest changes were observed for *Fusobacterium* (Fig. 5c and Extended Data Fig. 7a), which was mostly represented by *F.* *nucleatum* (Fig. 5d). Our analysis captured several additional taxa highly enriched in either tumor or healthy tissues (false discovery rate (FDR) < 0.05 and fold change > 2) (Fig. 5c and annotated in Supplementary Table 5). No major difference in α diversity (the variety and abundance of species within an individual sample) was observed between tumor and healthy samples (Extended Data Fig. 7b) and only a modestly reduced microbial diversity was observed in ICR-high versus ICR-low tumors (Extended Data Fig. 7b). *Selenomonas* and *Selenomonas 3* were the taxa most significantly increased in ICR-high versus -low tumors (Fig. 5e, Extended Data Fig. 7c and Supplementary Table 6). In terms of survival analysis, the highest number of nominally significant associations was obtained using tumor data (rather than healthy colon data) and OS as the end point (Extended Data Fig. 7d and Supplementary Table 7).

**Extended Data Fig. 8:**
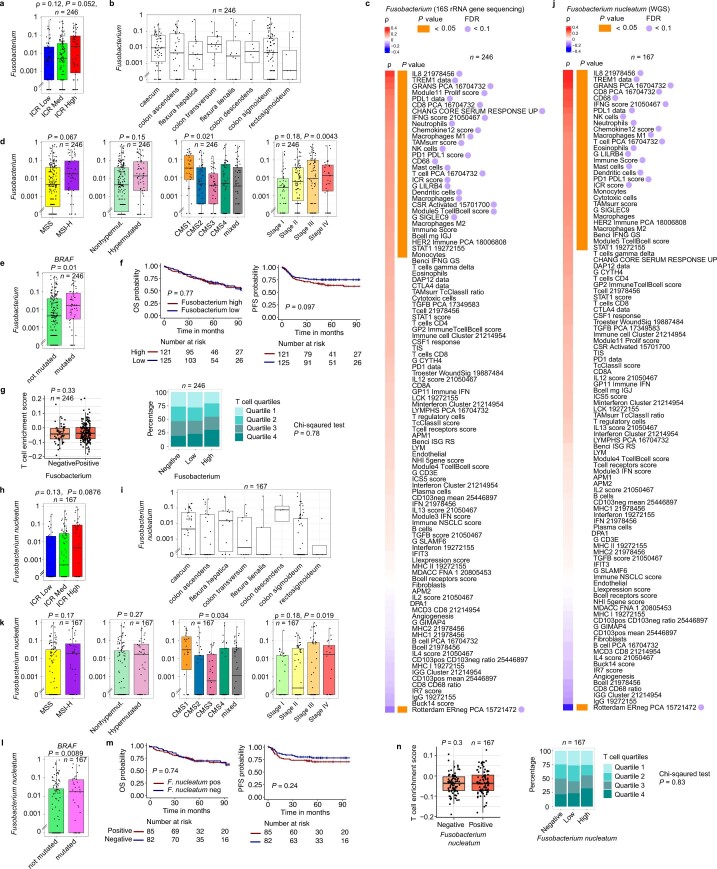
Relation between relative abundance of Fusobacterium and tumor characteristics in AC-ICAM. Relative abundance of Fusobacterium (derived from 16 S rRNA gene sequencing) **a**-**g**, and *Fusobacterium nucleatum* (derived from WGS) **h**-**m** in relation with tumor characteristic and the tumor microenvironment in AC-ICAM. **a**, Box-plot for relative abundance of Fusobacterium in tumor samples by ICR cluster. Spearman correlation statistics and corresponding *P* value is indicated. **b**, Box-plots for relative abundance of Fusobacterium in tumor samples by anatomical location. **c**, Spearman correlation between the relative abundance of Fusobacterium in tumor samples as determined by 16 S rRNA gene sequencing and immune gene signatures **d**, Relative abundance of Fusobacterium in tumor samples by MSI status, hypermutation status, CMS (CMS1 vs the rest, unpaired t-test, *P* = 0.021), and by pathological stage, *P* values are calculated using unpaired t-test. **e**, Relative abundance of Fusobacterium in tumor samples by BRAF mutation status. Green box is not mutated, pink box with nonsynonymous mutation. **f**, Kaplan–Meier curves corresponding to patients with tumor samples with a relative abundance of Fusobacterium above the median compared to those below the median. Overall *P* value is calculated by log-rank test. Vertical lines indicate censor points. **g**, T cell enrichment score (ssGSEA using Bindea et al, T cell signature) in tumor samples with absence of Fusobacterium (negative) or presence (positive) (left). Stacked bar chart of distribution of T cell quartiles by Fusobacterium categories (negative, low, high) (right). **h**, Same as **a**, but for *Fusobacterium nucleatum* as determined by metagenomic analysis of WGS. **i**, Same as **b**, but for *Fusobacterium nucleatum*. **j**, Same as **c**, but for *Fusobacterium nucleatum*. **k**, Same as **d**, but for Fusobacterium nucleatum. **l**, Same as **e**, but for *Fusobacterium nucleatum*. **m**, Same as **f**, but for *Fusobacterium nucleatum*. **n**, Same as **g**, but for *Fusobacterium nucleatum*. All *P* values are two-sided; *n* reflects the independent number of samples in all panels. Overall Survival (OS). Progression-Free Survival (PFS). For all box-plots: Center line, box limits, and whiskers represent the median, interquartile range and 1.5x interquartile range respectively.

*Fusobacterium* and *F.* *nucleatum* abundances were associated with advanced stage^17^, presence of *BRAF* mutations^43^, MSI-H status^17,44^ and a trend toward worse PFS survival (Extended Data Fig. 8)^17^, as previously observed. Instead of a negative correlation with T cells^44^, *Fusobacterium* or *F.* *nucleatum* abundances were associated with cytotoxic T cells and NK cells paralleled by an increase of myeloid markers and signaling (for example, *CD68*, *TREM1* and *IL8* signature). The lack of association with a favorable outcome might be explained by the ability of *F.* *nucleatum* to inhibit T and NK cell killing of tumor cells by binding and activating the inhibitory receptors TIGIT^45^ and CEACAM1 (ref. ^46^) or by induction of IL-8-mediated myeloid activation^47^ (Extended Data Fig. 8 and Supplementary Fig. 11).

### A microbiome signature (MBR score) predictive of survival

To detect clinically relevant associations between the microbial repertoire and clinical outcome, we aimed at identifying a microbiome signature predictive of survival using genus-level data from 16S rRNA gene sequencing, as part of our landmark microbiome analysis (AC-ICAM246, *n* = 246, testing set). On the AC-ICAM246, we ran a multivariable elastic-net OS Cox regression model that selected 41 features (taxa) with a coefficient different to zero (associated with differential risk of death; Methods). We termed this list of taxa and associated coefficients MBR classifier (Fig. 5f). A score was assigned to each sample (MBR score) by applying the MBR classifier. The MBR score displayed stability across different anatomic locations (in both tumor and healthy samples (Supplementary Fig. 12), despite the variable abundances of some taxa with respect to anatomic location; Supplementary Fig. 12d).

**Extended Data Fig. 9:**
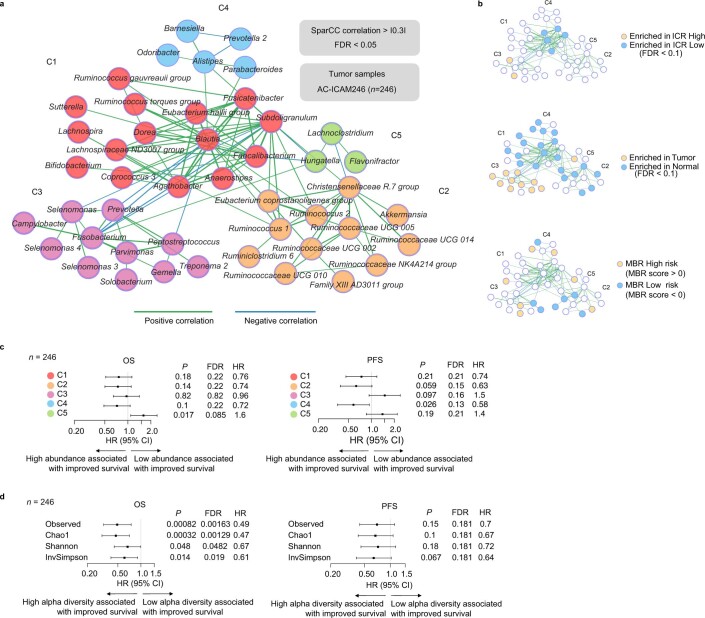
Co-occurrence network of microbial taxa and associations of identified clusters with biological and clinical parameters. **a**, SparCC co-occurrence network using centered log-ratio transformed OTUs in the AC-ICAM246 tumor samples. **b**, Overlay of network taxa with taxa enriched in ICR High or Low group (left panel), with taxa enriched in tumor vs normal colon samples (middle panel), and when present in the MBR classifier, either as low or high risk (right panel). **c**, Association between OS and PFS and the sum of the relative abundance of each genus (High vs low based on median) in each cluster in AC-ICAM246. **d**, Association between OS and PFS and distinct alpha diversity metrics (High vs low based on median) of the tumor microbiome in AC-ICAM246. HR (center), corresponding 95% confidence intervals (error bars) and corresponding *P* values are calculated by cox proportional hazard regression (**c**-**d**). All *P* values are two-sided; *n* reflects the independent number of samples in all panels. Overall Survival (OS). Progression-Free Survival (PFS).

Co-abundance network inference using SparCC^48^ correlation coefficients revealed five distinct clusters of taxa (Extended Data Fig. 9a). Taxa enriched in ICR-high versus ICR-low samples or in tumor versus healthy colon samples displayed high co-abundance (enriched in C3) and the same was observed for taxa enriched in healthy colon or in ICR-low samples (enriched in C1; Extended Data Fig. 9b). Low and high-risk taxa (according to MBR classifier) were spread across the different clusters (Extended Data Fig. 9b). Only marginal differences in survival were observed using estimates based on the cumulative abundance of genera belonging to each cluster identified by the network analysis (Extended Data Fig. 9c). The only survival association with an FDR <0.1 was detected for C5 (OS analysis, *P* = 0.017, hazard ratio (HR) 1.6, high versus low abundance, FDR = 0.085). C5 was constituted by three taxa, including one MBR-high-risk genera and no MBR-low-risk genera. Overall, these results suggest that clinical outcome is influenced by microbiome diversity, which is captured by the MBR classifier. Consistently, a high α diversity was associated with a prolonged OS FDR < 0.05 for all the α diversity estimates (Extended Data Fig. 9d).

**Extended Data Fig. 10:**
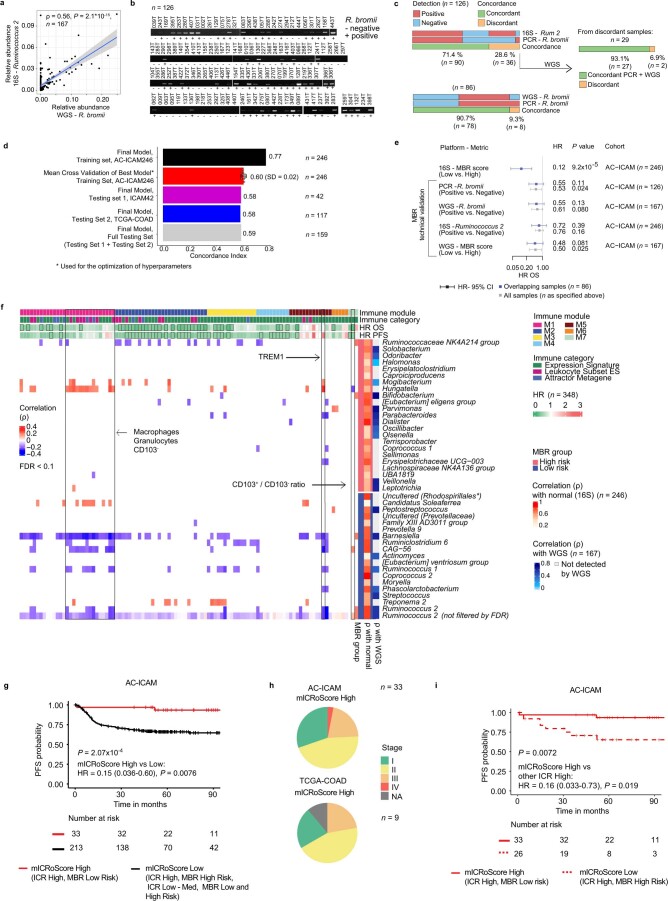
Technical validation of microbiome data, MBR and mICRoScore assessment, and correlation of MBR taxa with immune traits. **a**, 16 S rRNA gene sequencing versus WGS relative abundance of *Ruminococcus 2*. Spearman correlation and *P* value are indicated. The gray band reflects the 95% confidence interval for predictions of the linear regression model between the plotted variables. **b**, PCR gel images of 126 DNA samples amplified for *R. bromii*. **c**, Concordance between *R. bromii* PCR and detection of Ruminococcus 2 by 16 S rRNA gene sequencing or of *R. bromii* by WGS (positivity was defined as a relative abundance > 0). **d**, Concordance index of optimal multivariate cox regression model per dataset. The cross-validation performance highlights the mean concordance of 10-different folds with the optimal hyper parameters (gamma and lambda) that is, the same parameters as the optimal model. **e**, Forest plot with HR (center), corresponding 95% confidence intervals (error bars), and *P* value calculated by cox proportional hazard regression analysis for OS, using: 1) the 16 S MBR score in AC-ICAM, 2) WGS *R. bromii* abundance 3) PCR-based *R. bromii* abundance, 4) 16 S *Ruminococcus 2* relative abundance and 5) MBR score calculated using WGS data. **f**, Heat map of Spearman correlation between the relative abundance of the MBR classifier taxa in tumor samples and immune traits. Only correlations with an FDR > 0.1 are visualized. An additional row is added for Ruminococcus 2 showing all correlations, unfiltered for FDR. * The taxonomical order is indicated between brackets, as family was unassigned. **g**, Kaplan–Meier curve for PFS in AC-ICAM, with all patients stratified by mICRoScore High vs Low. HR and *P* value are calculated using cox proportional regression. **h**, AJCC pathological stage within the mICRoScore High group in AC-ICAM and within TCGA-COAD **i**, Kaplan–Meier curve for PFS in AC-ICAM, with all patients with ICR High stratified by mICRoScore. Overall *P* value is calculated by log-rank test and *P* value corresponding to HR is calculated using cox proportional hazard regression. Overall Survival (OS), Progression-Free Survival (PFS). All *P* values are two-sided; *n* reflects the independent number of samples in all panels. Source data

Because of the strong contribution of *Ruminococcus 2* to the MBR classifier, we sought to identify the actual *Ruminococcus* species. In WGS data, the *Ruminococcus* genus mostly consisted of *Ruminococcus* *bromii*, which also had the strongest correlation with *Ruminococcus 2* (Fig. 5g and Extended Data Fig. 10a). *R.* *bromii* presence was confirmed by PCR, which had strong correlation with sequencing data (for example, 91% concordance between WGS and PCR; Extended Data Fig. 10b,c).

### Validation of the MBR score

A low MBR score (MBR < 0, MBR low), in our training cohort (ICAM246, training set) was associated with a considerable (85%) reduction of risk of death (Fig. 5h). We confirmed the association between MBR low (risk) and prolonged OS in two independent testing sets (ICAM42 and TCGA-COAD cohorts), individually and combined (Fig. 5h,i, testing sets). The performance of the final MBR model was lower on the test sets than on the training set, which is typical for machine-learning models (Extended Data Fig. 10d); however, the concordance index of the final MBR model in both the test sets were superimposable to the ones obtained via cross-validation of the best MBR model on the training set (Extended Data Fig. 10d), substantiating that the model can generalize well to new (unseen) data.

A similar, but less-pronounced trend in terms of reduction of the risk of death was detected by simply using intratumoral *Ruminococcus 2* (based on 16S data) or *R.* *bromii* presence (based on either PCR or WGS data) (Extended Data Fig. 10e). Intratumoral *Ruminococcus 2* and MBR score, which strongly correlated with each other, were similar in tumor and healthy colon tissues (Fig. 5j).

**Fig. 6:**
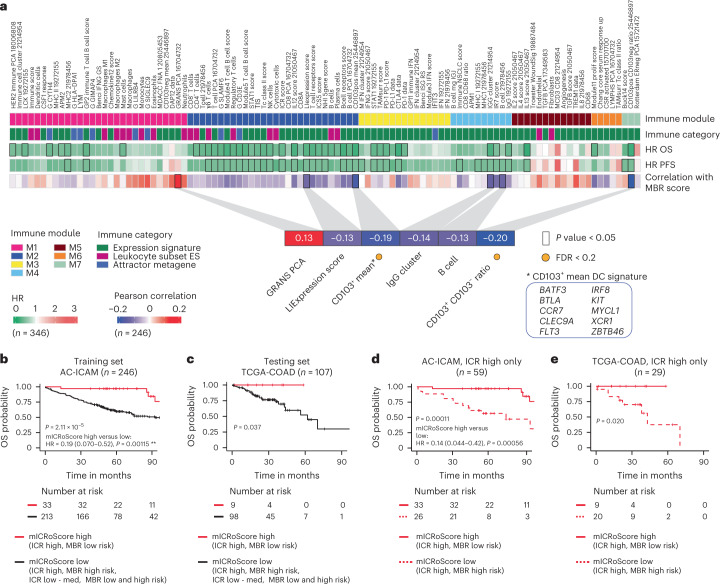
Correlation between MBR score and immune traits and development and validation of the mICRoScore. **a**, Visual representation of the associations between immune modules, immune categories, OS, PFS and the Pearson correlation between the MBR score and the immune traits. Inset highlights the significant Pearson correlations (*P* < 0.05), associations with FDR < 0.2 are indicated with a yellow dot. IFN, interferon; ES, enrichment score. **b**,**c**, Kaplan–Meier curves of OS by mICRoScore in AC-ICAM (**b**) and TCGA-COAD (**c**). **d**,**e**, Kaplan–Meier curve of OS in ICR-high samples by mICRoScore in AC-ICAM (**d**) and TCGA-COAD (**e**). Overall *P* value is calculated by log-rank test. Vertical lines indicate censor points. HRs and 95% confidence intervals are calculated by Cox proportional hazard regression. All *P* values are two-sided; *n* reflects the independent number of samples.

The relationship between the microbiome and clinical outcome pointed to an interaction between the microbiome and biological processes occurring in the tumor. When correlating immune trait values with the MBR score, the strongest (inverse) correlation with the MBR score was observed for signatures capturing the prevalence of CD103^+^ dendritic cells (DCs) with unique antigen processing and presentation capabilities for efficient antigen cross-presentation to CD8^+^ T cells (CD103^+^, mean signature (*P* = 0.003) and CD103^+^ signature to CD103^−^ signature ratio (*P* = 0.001)) (Fig. 6a and Supplementary Table 8)^49^. Consistently, correlation analyses between individual taxa included in the MBR classifier and immune traits demonstrated, with few exceptions, a positive correlation with myeloid signatures and a negative correlation with the CD103^+/^^−^ ratio for taxa with positive MBR coefficient (higher risk of death), while the reverse was observed for taxa with a negative MBR coefficient (Extended Data Fig. 10f).

### Development and validation of the mICRoScore

We then sought to develop a multi-omics parameter that could capture a subgroup of patients with exceptional survival.

Among single-omics parameters that were significant in the univariate Cox regression OS analysis (ICR, MBR and GIE categories), only ICR and MBR were retained by the multivariable Cox models (*P* < 0.05; Supplementary Table 9) adjusted for age, CMS subtypes, stage and MSI status. MBR and ICR were therefore combined into an integrated score (mICRoScore*)*.

Indeed, in the training cohort (AC-ICAM246), the co-presence of ICR high and MBR low (mICRoScore high) identified a subgroup of patients with a 97% 5-year OS, with only three deaths detected at a later follow-up (Fig. 6b) that were not related to colon cancer (Extended Data Fig. 10g). No deaths were observed during the entire follow-up in patients with mICRoScore high in the TCGA-COAD cohort (*n* = 107, testing set; Fig. 6c). In both the training (AC-ICAM) and the testing (TCGA-COAD) sets, the mICRoScore-high group consisted of patients at different stages (Extended Data Fig. 10h). The additive effect of the two parameters was due to the ability of MBR to segregate ICR high into two distinct risk categories (Fig. 6d,e and Extended Data Fig. 10i).

## Discussion

Our multi-omics approach allowed us to thoroughly examine the molecular characteristics of immune responsiveness in colon cancer and uncover interactions between the microbiome and the immune system. We found that a T_H_1 cell/cytotoxic immune activation, as captured by the ICR, immunoediting, concurrent expansion of TCR clonotypes and specific intratumoral microbiome composition, were associated with a favorable clinical outcome. ICR was associated with OS independently of MSI and CMS, which both lost statistical significance in the multivariate analysis. Its prognostic impact increased when combined with a metric capturing the genetic immunoediting (IES).

Using deep TCR sequencing in tumor and healthy tissues, we showed that the prognostic effect of ICR could be due to its ability to capture the presence of tumor-enriched and possibly tumor-antigen specific, T cell clones.

The AC-ICAM addressed the limitations of the TCGA colon cancer cohort noted by the scientific community^8–10^ and corroborated by our comparative analyses. While several studies have described associations between response to immunotherapy and the gut microbiome^50^ and identified cancer-specific microbiome compositions^12,13,51^, comprehensive microbiome analyses focused on patients with primary colon cancer are lacking. By analyzing the tumor microbiome composition using 16S rRNA gene sequencing in AC-ICAM samples, we identified a microbiome signature (MBR risk score) with strong prognostic value. This signature was derived from tumor samples, but there was a strong correlation between the healthy colon and tumor MBR risk scores, suggesting that this signature may capture the patient’s gut microbiome composition.

Additional analysis and technical validation using orthogonal platforms such as WGS and PCR indicated that the detected signal was driven by *R.* *bromii*. Correlation analyses between the MBR risk score and immune traits suggest a specific positive modulation of CD103^+^ dendritic cells, which are critical for antitumor immune responses. We speculate that the identified consortium of bacteria favors optimal T cell priming mediated by CD103^+^ dendritic cell activation and suppression of the myeloid compartment, leading to the induction of a partially protective antitumor immunity.

By combining the ICR and MBR scores, we were able to identify and validate a multi-omics biomarker (mICRoScore) that could predict exceptionally long survival in patients with colon cancer.

Studies on the gut microbiome compositions of patients receiving immunotherapy, including anti-CD19 CAR T cell treatment^52^, have shown favorable associations with *Ruminococcus* and or *R.* *bromii* and response^53–55^. Here, we propose the *R.* *bromii* as the possible link between prognostic and predictive microbiome-based signatures. Our findings support the testing of adjuvant microbiota-targeted/dietary interventions^56,57^ aimed at decreasing the risk of recurrence and death in patients with colon cancer through the induction of an antitumor response against minimal residual disease. These approaches might also be investigated in the context of neoadjuvant immunotherapy^58^.

For example, data from breast and sarcoma mouse models suggest that the gut microbiome can be enriched with *R.* *bromii* through the administration of castalagin (an ellagitannin found in certain aliments including the berry *Myrciaria* *dubia*), resulting in enhanced antitumor immunity, possibly mediated by boosting antigen presentation and T cell response^59^.

Administration of *Myrciaria* *dubia* powder concomitantly with immune checkpoint inhibitors is currently being explored in patients with melanoma and non-small-cell lung cancer (NCT05303493).

Our study has some notable limitations. While the cohort was relatively large and compares favorably with the TCGA-COAD colon cohort (for example, ~50% OS events more in AC-ICAM versus TCGA-COAD^60^), it remains underpowered for stage-specific survival analysis. For the mICRoScore, we were unable to assess and quantify potential data overfitting as we did not reserve internal samples for this purpose; however, we observed a good performance of the mICRoScore in the external validation cohort (TCGA-COAD), which may be due to the combination of two biologically relevant variables (ICR and MBR) into the model. This combination likely contributed to the model’s impact and suggests that the mICRoScore might be generally applicable. We did not perform in situ spatial profiling, which could reveal more complex spatial immune–microbiome interactions^61^. Additional research is needed to confirm the validity of the mICRoScore and investigate its potential applications in clinical treatment decision-making. Both the mICRoScore and IES could be tested in the context of cancer immunotherapy as predictive biomarkers. Data from the NIBIT-M4 trial and publicly available datasets suggest that the combination of the genetic immunoediting and ICR (IES) has predictive value in melanoma patients treated with immune checkpoint inhibitors^62^. The quantification of the immunoediting using WES data is an emerging subject of research^63,64^. These scores might also be explored to define a subgroup of patients with stage III tumors that could be eligible for a reduced chemotherapy regimen.

In conclusion, the AC-ICAM provided insight into the biology of colon cancer that could be utilized to establish clinical-grade prognostic or predictive biomarkers and to identify targeted therapies for personalized treatment approaches. We hope that further exploitations of our resource by physicians and scientists around the globe will lead to the discovery of new concepts within cancer research, ultimately improving life expectancy of patients suffering from this frequent and aggressive disease.

## Methods

Samples used in this observational cohort study (tumor tissue and matched healthy colon tissue, AC-ICAM cohort) are from patients with colon cancer diagnosed at Leiden University Medical Center, the Netherlands, from 2001 to 2015 that did not object for future use of human tissues for scientific research and that were consented on biospecimen protocol ‘Immunology and Genetic of colon Cancer’ approved by the Committee on Medical Ethics of Leiden University Medical Center (study protocol no. P00.193 (06/2001)). Snap-frozen tumor and healthy colon tissue were stored at −80°C until processing for DNA and RNA extraction. DNA and RNA from those samples were extracted at Leiden University Medical Center and then transferred to Sidra Medicine for sequencing together with de-identified clinicopathological data of the corresponding patients (Sidra Medicine IRB study protocols no. 1768087-1 (04/2016)/1602002725 (06/2022)). All genomic assays (WES, WGS, 16S RNA gene sequencing, RNA-seq, TCR sequencing and PCR) were performed at Sidra Medicine.

Patient information was de-identified and patient samples were anonymized and handled according to the medical guidelines described in the Code of Conduct for Proper Secondary Use of Human Tissue of The Federation of Dutch Medical Scientific Societies. This research was performed according to the recommendations outlined in the Helsinki Declaration.

For each assay we included all samples that had sufficient material (for example, DNA or RNA) available at the time of processing considering the need to preserve aliquots for additional/future assays.

### Collection of biological samples

Snap-frozen tumor and healthy colon tissue were collected from patients with colon cancer who underwent surgical resection of the primary tumor between 2001 and 2015 at Leiden University Medical Center. Patients who received radiotherapy and/or chemotherapy before resection and patients with a primary tumor of non-epithelial origin were excluded. Based on tissue availability, successful nucleic acid extraction and subsequent sequencing quality control (QC), data from 348 patients were retained in the final AC-ICAM cohort (Extended Data Fig. 1). Clinicopathological and follow-up data were retrospectively collected from hospital records. Patient information was de-identified and patient samples were anonymized and handled according to the medical guidelines described in the Code of Conduct for Proper Secondary Use of Human Tissue of The Federation of Dutch Medical Scientific Societies. Extensive clinicopathological and survival data of the cohort are available (Supplementary Table 1).

### Statistical analysis

Details of the statistical analysis are described in each method section. All *P* values were two-sided. Multiple testing corrections were performed by calculating the FDR using the Benjamini–Hochberg method, as appropriate. For missing data, no data imputations were used.

### Survival analysis

Kaplan–Meier curves were generated using ggsurvplot from R package survminer (v.0.4.9). HRs between any two groups of interest and corresponding *P* values based on a Cox proportional hazard regression analysis and 95% confidence intervals (95% CI), were calculated using R package survival (v.2.41–3). Cox proportional hazard analysis was only computed when both groups of comparison consisted of at least ten patients. Overall *P* value for comparison of survival between two or more groups was also calculated by log-rank test.

Multivariate Cox regression was performed using conventional clinical and biological variables, as explained in the specific section. Separate multivariate Cox regression analyses were run including age (continuous), pathological stage (ordinal), MSI status (binary) and CMS (categorical). Additional variables that were found significant in univariate Cox proportional hazard regression analysis were added to these models. These variables included, ICR score (continuous) or ICR cluster (ordinal), GIE (binary) and MBR group (binary). Forest plots were generated using ‘forestplot’ (v.1.7.2).

### Tissue processing

Tumor and healthy tissue samples (unselected for tumor cell purity) were sectioned in a cryostat until the surface area was sufficient to assess tissue morphology by H&E staining. Non-target tissue was removed by macrodissection, including necrotic or adipose tissue and for tumor tissue samples, healthy colon tissue. When macrodissection was required, an H&E-stained slide was examined after this to confirm removal of unwanted tissue types. Frozen tissue was then sectioned at 20 µm until approximately ~10–15 mg was collected per sample. A final section post-sample processing was made for H&E staining. The collected tissue was stored at −80 °C for a few months until DNA and RNA extraction.

QC metrics of RNA and DNA data were superimposable between samples collected over the years (Supplementary Figs. 13 and 14).

### DNA and RNA extraction

Nucleic acid extraction from fresh-frozen tissue sections was performed using the QIAGEN AllPrep DNA/RNA Mini kit following the manufacturer’s protocol. This process was fully automated on a QIAGEN QIAcube. β-mercaptoethanol (β-ME) was added to the lysis buffer on the day of use. Lysis was performed by completely submerging the sections in 350 µl lysis buffer. Tubes were rotated for at least 1 h at room temperature to allow complete homogenization. QIAcube AllPrep DNA/RNA Mini kit Standard (v.2) program was run, after which DNA and RNA samples were stored at −80 °C. The same DNA was used for human and microbiome sequencing. Samples were shipped from Leiden University Medical Centre (LUMC), The Netherlands to Sidra Medicine, Qatar under a temperature-controlled environment at −80 °C (for 4 d). Samples from 361 patients were sequenced by WES and RNA-seq. Samples from 13 patients were excluded as they did not pass QC, including concordance between healthy and tumor samples (Extended Data Fig. 1). The final cohort included 348 patients, for which RNA-seq for tumor samples was possible and passed QC. A subset of samples from these patients were processed with additional assays including WGS, TCR sequencing and 16S RNA gene sequencing, based on the availability of samples for these assays, as described in the following sections.

### RNA sequencing

The integrity and concentration of the extracted RNA was assessed on the LabChip GXII Touch HT using the RNA Assay and the DNA 5K/RNA/Charge Variant Assay LabChip (PerkinElmer). Sequencing mRNA libraries were constructed from 500 ng of total RNA using the Illumina TruSeqStranded mRNA kit (Illumina). cDNA was synthesized using Superscript IV Reverse Transcriptase (Thermo Fisher) and amplified for 15 cycles after ligating with TruSeq RNA Combinatorial Dual-Index adapters. Clonal amplification and cluster generation was performed using Illumina’s cBot 2 System. Sequencing libraries were run on Illumina HiSeq platforms using 75 bp (93% of samples) or 150 bp (7% of samples) paired-end reads at the Clinical Genomics Laboratory, Sidra Medicine. We targeted a coverage of 20 M reads per sample. Obtained coverage was 18.4 M (s.d. 4.7 M).

### Transcriptomic data processing

Data conversion and demultiplexing was performed using bcl2fastq2 conversion software (v.2.20). FastQC was run to perform QC checks on the raw sequence data (Python v.2.7.1, FastQC v.0.11.2). Trimming of adaptor sequences was performed using flexbar (v.3.0.3) using Illumina primers FASTA file. Subsequently, reads were aligned to reference genome GRCh38.93 by Hisat2 (v.2.1.0) using SAMtools (v.1.3). After alignment, QC was performed to verify quality of the alignment and paired-end mapping overlap (Bowtie2, v.2.3.4.2). Finally, the featureCounts function of subreads (v.1.5.1) was used to count paired reads per genes. Gene expression normalization was performed within lanes, to correct for gene-specific effects (including GC content) and between lanes, to correct for sample-related differences (including sequencing depth) using R package EDASeq (Exploratory Data Analysis and Normalization for RNA-seq) (v.2.12.0). The resulting expression values were quantile normalized using R package preprocessCore (v.1.36.0). All downstream analysis of the expression data was performed using R (v.3.5.1, or later).

### Whole-exome sequencing

DNA concentrations were quantified using Quant-iT broad range dsDNA Assay (Thermo Fisher) on the FlexStation 3 Microplate reader (Molecular Devices). DNA of both tumor and matched normal samples was available for 294 patients. Whole-exome libraries were constructed with the Agilent SureSelect XT Target enrichment kit and the exonic DNA was captured using the Agilent SureSelect XT Human All Exon V6r2 capture library for 60-Mb exonic regions. Libraries were constructed using 250 ng of DNA and were sequenced on Illumina’s HiSeq 4000 platform using 150 bp paired-end reads (150PE) at the Genomics Core, Sidra Medicine. Reads were mapped to reference genome hs37d5 (1000 Genomes Phase2 Reference Genome Sequence) based on GRCh37/hg19 using BWA (v.0.7.12)^65^. WES (200× for tumor and 100× for normal) had an on-target sequencing rate of 65–70%. The median (across samples) of the average target coverage (per sample) was 129× (interquartile range (IQR) 18) for tumor samples and 69× (IQR 10) for normal samples (Extended Data Fig. 5a). In tumors, sequencing achieved >20-fold coverage of at least 99% of targeted exons and >70-fold in at least 81% targeted exons. In healthy samples, sequencing achieved >20-fold coverage of at least 94% of targeted exons and >30-fold in at least 84% targeted exons. Adaptor trimming was performed using the tool trimadap (v.0.1.3). ConPair was run to evaluate concordance and estimate contamination between matched tumor–normal pairs. In eight of the pairs a mismatch was detected and for five pairs, a potential contamination was indicated. HLA typing data were used to validate these results. All potential mismatches and contaminations were excluded, retaining 281 patients for data analysis.

### TCGA data

#### RNA sequencing

RNA-seq data (raw counts) from TCGA were downloaded and processed using R package TCGAbiolinks (v.2.18.0). Gene symbols were converted to official HGNC gene symbols and genes without symbol or gene information were excluded. Normalization was performed within lanes, to correct for gene-specific effects (including GC content) and between lanes, to correct for sample-related differences (including sequencing depth) using R package EDASeq (v.2.12.0) and quantile normalized using preprocessCore (v.1.36.0). After normalization, samples were extracted to obtain a single primary tumor tissue (TP) sample per patient. Clinical data were sourced from the TCGA Pan-Cancer Clinical Data Resource^11^ and survival events OS and progression-free interval (relabeled here as PFS) were used. ICR clustering and calculation of ICR score was performed exactly as described for the AC-ICAM cohort. For the TCGA-COAD cohort, the optimal number of clusters for best segregation based on the Calinski–Harabasz criterion was three. CMS classification of TCGA-COAD samples was performed as described for the AC-ICAM cohort. The Single Sample Predictor by ‘CMSclassifier’ (v.1.0) was used for comparison of CMS classification between AC-ICAM and TCGA-COAD.

A renormalized matrix of both TCGA-COAD and AC-ICAM datasets was generated by merging the raw counts matrices and performing the EDASeq normalization, as described above, on this combined matrix. These data were used to calculate ssGSEA scores for deconvoluted immune cell subpopulations, immune signatures and oncogenic pathways, to compare between cohorts.

#### Somatic mutation data

Somatic mutation calls from the TCGA MC3 Project were downloaded using R package TCGAmutations (v.0.3.0) using the function tcga_load() with parameters ‘COAD’ for study and ‘MC3’ for source. The downloaded Mutation Annotation Format (MAF) file contained 406 distinct TCGA tumor sample barcodes and 18,183 genes (Hugo Symbol). This file was filtered to only include nonsynonymous mutations (‘Frame_Shift_Del’, ‘Frame_Shift_Ins’, ‘In_Frame_Del’, ‘In_Frame_Ins’, ‘Missense_Mutation’, ‘Nonsense_Mutation’, ‘Splice_Site’, ‘Translation_Start_Site’, ‘Nonstop_Mutation’), analogous to the variant filter applied to the AC-ICAM somatic mutation calls.

#### Microbiome

Microbiome genus relative abundance matrix for TCGA-COAD cohort (125 tumor samples and 221 genera, WGS data) was downloaded from The Cancer Microbiome Atlas website ^13^. TCGA-COAD relative abundance matrix was filtered to exclude duplicated samples (samples from vial B, eight samples). Overall, 81 genera were present with a nonzero abundance in at least one of the 117 samples (main matrix). When we applied the same filter as the one used for AC-ICAM 16S RNA gene-sequencing data (presence in at least 10% of the samples with at least 1% relative abundance in one sample), 27 taxa at the genus level were retained.

### NHS and the HPFS study data

#### Somatic mutation data

Somatic mutations in NHS and HPFS Colorectal Cancers were downloaded from the supplementary data of the Giannakis et al. study (Giannakis, Supplementary Table 3). The downloaded file contained 619 distinct tumor sample barcodes and 19,208 genes (Hugo Symbol). We excluded the samples with tumor anatomic site specified as rectum (anatomic site is available in Giannakis Supplementary Table 1) and retained 482 colon cancer samples. Only nonsynonymous mutations were included at the variant filter (‘Frame_Shift_Del’, ‘Frame_Shift_Ins’, ‘In_Frame_Del’, ‘In_Frame_Ins’, ‘Missense_Mutation’, ‘Nonsense_Mutation’, ‘Splice_Site’, ‘Translation_Start_Site’, ‘Nonstop_Mutation’), analogous to the variant filter applied to the AC-ICAM and TCGA-COAD somatic mutation files.

### Cancer-related gene annotation

A cancer-related gene list was constructed from using different sources, as previously described:^35^ (1) genes used by two consortia to define germline genetic variations in pediatric cancers (*n* = 159;^34^ *n* = 565 (ref. ^33^)); (2) genes with at least one pathogenic or likely pathogenic germline variants in the TCGA cohort (*n* = 99)^66^; (3) genes classified as driver genes according to the most updated TCGA analysis (*n* = 299)^32^; (4) genes included in the MSK-IMPACT (*n* = 505), MSK-IMPACT HEME (*n* = 575), Foundation One CDx (*n* = 324) and Foundation One Heme (*n* = 593) panels; (5) cancer genes cataloged as tier 1 by the Sanger Cancer Gene Census (*n* = 576); and (6) cancer genes defined as such by Vogelstein et al.^67^. Sources 4–6 were downloaded from OncoKB^68^. Original sources’ gene names were converted into Ensemble GRCh37 gene symbols. The final list included 1,219 unique cancer genes and is provided in the Supplementary Information.

### Transcriptome analysis

#### ICR score and clustering

Consensus clustering based on 20 a priori selected ICR genes (*IFNG*, *IRF1*, *STAT1*, *IL12B*, *TBX21*, *CD8A*, *CD8B*, *CXCL9*, *CXCL10*, *CCL5*, *GZMB*, *GNLY*, *PRF1*, *GZMH*, *GZMA*, *CD274/PDL1*, *PDCD1*, *CTLA4*, *FOXP3* and *IDO1*)^21^, was applied to the normalized log_2_-transformed expression matrix using R package ConsensusClusterPlus (v.1.42.0)^69^ using 5,000 repeats, agglomerative hierarchical clustering with Ward criterion inner and complete outer linkage. The optimal number of clusters allowing for the best segregation of samples was based on the Calinski–Harabasz criterion. Optimal number of clusters used for segregation was three. Colon cancer samples in the cluster with the highest expression of ICR genes were designated as ‘ICR high’, the intermediate cluster as ‘ICR medium’ and the cluster with the lowest expression was designated ‘ICR low’. The mean log_2_-transformed expression value of the 20 ICR genes is referred to as the ICR score.

#### CMS classification

Samples were classified according to CMS by R package ‘CMSclassifier’ (v.1.0) using random forest method^16^. The obtained CMS labels (from the column ‘RF.predictedCMS’ in output dataframe) were used for all downstream analyses with the exception of the comparison of CMS subtypes between AC-ICAM and TCGA cohort. To allow between-cohort comparison, we ran the CMSclassifier using the ‘single-sample predictor’ method. This method makes it possible to predict unique samples, with a constant output whether the sample is predicted alone or within a series of samples^16^ and can therefore be used for comparison across cohorts.

Dimension-reduction of the complete expression matrix was performed using *t*-SNE by ‘Rtsne’ (v.0.15) and visualized using ggplot2 (v.3.3.2). The *t*-SNE plot was annotated with distinct colors to visualize the distribution of samples of different CMS (using random forest method) in high-dimensional space. The same *t*-SNE plot was annotated by ICR cluster in a separate panel. A circos plot to visualize the relation between CMS and ICR classifications was generated using the chordDiagram function from R package ‘circlize’ (v.0.4.8).

#### Immune cell deconvolution and ESTIMATE

Consensus tumor microenvironment cell estimation (ConsensusTME)^70^ was performed to estimate relative abundancies of specific immune cell subsets from bulk transcriptome data. This method relies on integrated gene sets from multiple sources that have been curated and validated on a per-cancer-type basis, using benchmark datasets and seems to outperform previously published methods^70^. We applied ConsensusTME using R package ConsensusTME (v.0.0.1.9) using parameters ‘COAD’ to specify cancer type and ‘ssgsea’ as statistical method.

The median of each ConsensusTME score was calculated per CMS stratified by ICR cluster and was displayed in a dotted heat map using R package ComplexHeatmap (v.2.1.2). The association of each ConsensusTME score with OS and PFS was calculated by Cox proportional hazard regression. HR and corresponding 95% CIs as are displayed as forest plots (forestplot v.2.0.1).

To infer estimated levels of overall stromal and immune cell infiltration to the tumor, the ESTIMATE algorithm (v.1.0.13) was applied to the expression data in R. ESTIMATE was run for both TCGA-COAD dataset and the AC-ICAM cohort. The combined ESTIMATE score for both the stromal and immune signature was compared between cohorts and a box-plot was generated using ggplot2 (v.3.3.2).

#### Analysis of tumor-related signatures and immune traits

Single-sample gene set enrichment analysis (ssGSEA) was applied to the log_2_-transformed, normalized gene expression matrix^71^ (GSVA, v.1.38.2). Gene sets that reflect specific tumor-related pathways were selected from multiple sources as described in detail in Roelands et al.^10^ and Supplementary Source Data Table 6a. Enrichment scores of each of these 48 pathways by CMS were visualized using ComplexHeatmap (v.2.1.2). To better understand the interactions between tumor-intrinsic signaling and the immune microenvironment, we calculated the Pearson correlation between the ICR score and the scores of the 48 tumor-related pathways. This analysis was performed in the total cohort as well as across CMS subtypes.

Immune traits considered for analysis were based on a collection of well-characterized immune traits^3,72^. This collection includes 68 gene signatures related to immunomodulatory signaling, including IFN signaling, TGF-β, wound healing (core serum response) and T cell/B cell response^3,73^. Gene expression values were median centered and gene symbols were mapped to EntrezIDs (org.Hs.eg.db_3.6.0). Signatures scores were then mean centered and their s.d. values were scaled to one. For all other immune traits, ssGSEA was applied. These included signatures for antigen-presenting machinery (APM1 and APM2) and angiogenesis and nine TCGA-based coexpression signatures (metagene attractors). This collection was supplemented with the tumor inflammation signature^9^ and two non-overlapping signatures of IFN-stimulated genes (ISGs), including IFNG hallmark gene set IFNG.GS and ISG resistance signature (ISG.RS)^74^, calculated using ssGSEA. Finally, the deconvoluted immune cell abundancies by ConsensusTME^70^ and ICR score^10^ were included among the immune traits. In total we used 103 immune traits (including ConsensusTME) (Supplementary Source Data 6 provides gene signatures and corresponding references).

The pairwise Pearson correlation between all immune traits was calculated and the resulting correlation matrix was plotted using ComplexHeatmap (v.2.1.2) with hierarchical clustering. Co-clustering immune traits that formed distinct modules were visualized and labeled according to the immune traits’ enrichment. The clustering was compared to previously defined immune trait modules within a pan-cancer setting, by annotation of the correlation matrix with the previously defined clusters in Sayaman et al.^3^.

#### Survival analysis on AC-ICAM subsampling

We subsampled AC-ICAM hundreds of times in two ways, one was random, the other was on a subgroup of samples with an ESTIMATE distribution that approximates that of the TCGA-COAD. The function ‘approxfun’ in R was used to generate a function to approximate the density of ESTIMATE scores in TCGA-COAD. Cases were sampled from AC-ICAM using the ‘sample’ function in R with prob argument set to sample points with probability distribution of the TCGA-COAD. Each subsampled cohort consisted of 200 samples. The number of subsets in which the Cox proportional regression for ICR score was significant was compared between the two ways of subsampling, statistical significance was determined using a chi-squared test.

### TCR targeted sequencing by immunoSEQ assay

This sensitive and specific dedicated assay requires high quantity of genomic DNA (>2 µg) and sample selection was exclusively based on DNA availability. TCR sequencing was performed using extracted DNA of 114 primary tissue samples and ten matched healthy colon tissues with sufficient DNA available.

DNA samples were normalized to a concentration of 125 ng μl^−1^ using 3.840 μg of DNA as input per sample. The immunoSEQ assay from Adaptive Biotechnologies was used to amplify all possible variable, diversity and joining (VDJ) gene rearrangements of the TCRβ locus (*TRB*) using a multiplex PCR method. PCR and magnetic bead cleanup were performed according to manufacturer’s instructions. Recommended QC was performed after the first PCR and second PCR amplification steps by running the PCR product on an agarose gel. Purified second PCR amplification products were pooled and the library pool was quantified using Agilent Bioanalyzer 2100. Subsequently, pools were diluted to a concentration of 1 pM and sequenced on Illumina NextSeq 500/550 system with Mid Output kit (150 cycles) and Custom NextSeq Sequencing Primer (P/N, M150) (read 1, 156 cycles and read 2, 9 cycles). Sequencing was performed using survey resolution (two replicates per sample). A sample manifest was created in immunoSEQ Analyzer and the raw sequencing data were uploaded to the Adaptive Biotechnologies cloud following the manufacturer’s instructions. Data were processed using the company’s proprietary pipeline. Number of total templates analyzed per sample ranged 1,906–95,834 (median 21,258). The average read coverage per sample ranged 11.4–80.6 (median 36.2).

### TCR analysis

#### TCR immunoSEQ data analysis

ImmunoSEQ sample-based output variables, as made available by the immunoSEQ Analyzer, include the total number of templates analyzed, number of productive templates, fraction productive templates, number of total rearrangements, number of productive rearrangements, productive clonality and the maximum productive frequency. Herein, the total number of templates reflects the total number of T cells analyzed, of which only the productive templates can produce a functional protein receptor (rearrangement in the sample are inframe and do not contain a stop codon). The total number of productive rearrangements is the total number of unique T cell clones and clonality is calculated by normalizing the productive entropy using the total number of productive rearrangements and subtracting the result from 1. Values for (productive) clonality range from 0 to 1, with values near 0 reflecting more polyclonal samples and values near 1 representing samples with just few predominant rearrangements dominating the observed T cell repertoire (*TRB* gene). A high T cell clonality implies presence of expanded T cell clones.

Relationships between ICR score, immune traits, number of productive templates and productive clonality were tested using Pearson’s correlation and visualized by scatter-plots using ggplot2 (v.3.3.2). Similarly, Pearson’s correlation coefficient was calculated between productive clonality and each of the 18,270 genes in the expression matrix. A volcano plot was used to visualize significant results (ggplot2). The top 50 genes with the highest correlation with TCR productive clonality were mapped to the Global Molecular Network and core network analysis was performed using Ingenuity Pathway Analysis software.

Data on all productive rearrangements per sample were exported from the immunoSEQ Analyzer Rearrangement Details View. This file includes the exact nucleotide sequence generated through V(D)J recombination, corresponding amino acid sequence, number of templates and productive frequency. Overlapping TCR sequences between tumor samples and matched healthy colon tissues (*n* = 9) were evaluated and visualized by scatter-plots (ggplot2). Sequences with a productive frequency at least 32-fold higher in the tumor compared to the healthy colon tissue and a tumor productive frequency >0.1% were defined as tumor-enriched sequences, as previously implemented by Beausang et al.^75^. The fraction of tumor-enriched TCR sequences in the tumor was calculated by dividing the number of productive templates of tumor-enriched sequences by the total number of productive templates per tumor sample. Pearson’s correlation coefficient between the fraction tumor-enriched TCR sequences and ICR score was calculated.

#### MiXCR for TCR repertoire derived from bulk RNA-seq

The software MiXCR (v.3.0.13)^30^ was used to retrieve the VDJ repertoire from bulk RNA-seq data aligned to reference genome GRCh37. MiXCR was run through docker and with the single command analyze shotgun. The R package ‘immunarch’ was used to analyze the MiXCR output into the R environment. For the TCRβ locus (*TRB*), the TCR clonality was calculated as 1 − normalized Shannon entropy (see Calculation section for details) for all samples, except seven cases for which MiXCR failed to identify clones.

### Whole-exome-sequencing data analysis

#### Somatic mutation calling and small insertions and deletions

SNVs were called using mutect (v.1.1.7) and somatic small insertions and deletions (indels) using strelka2 (bcbio-nextgen v.1.1.1). We applied an optimized variant filtering pipeline (Extended Data Fig. 5b). To filter out false-positive single-nucleotide polymorphism calls, fpfilter was used, the applied filtering parameters are specified in the fpfiler.pl script shared on GitHub. Subsequently, MAF files were generated using VCFtoMAF tool (v.1.6.16), which also appended the SIFT (sorting intolerant from tolerant), PolyPhen and Exome Aggregation Consortium annotations. MAF files were loaded into R where indels with low complexity regions were excluded. For both SNVs and indels, a cutoff for minimum allele fraction of 5% and tumor depth of more than three reads was applied. The Exome Aggregation Consortium data were then used to filter out common variants that are encountered in >1% in the general population. After these technical exclusion criteria, biological filters were applied, including selection of nonsynonymous mutations (frame shift deletions, frame shift insertions, inframe deletions, inframe insertions, missense mutations, nonsense mutations, nonstop mutations, splice site and translation start site mutations). The resulting number of variants/mutations per Mb (capture size is 40 Mb) per sample is referred to as the nonsynonymous TMB. Next, to identify most frequently mutated genes in our cohort that might play a role in cancer, we excluded variants that are predicted to be tolerated according to SIFT annotation or benign according to PolyPhen (polymorphism phenotyping). Finally, all artifact genes, which are typically encountered as bystander mutations in cancer that are mutated for example as a consequence of a high homology of sequences in the gene, were excluded^76^. The OncoPlot function from ComplexHeatmap (v.2.1.2) was used to visualize the most frequent somatic mutations.

#### Comparison of TMB with TCGA datasets

To compare the TMB in the AC-ICAM with all 33 TCGA cohorts derived from the MC3 project, we used the tcgaCompare function from maftools (v.2.6.05, R). For AC-ICAM, the filtered MAF for nonsynonymous mutations was used as input with specified capture size of 40.

#### Comparison of somatic mutations with other cohorts

To define mutated genes in the AC-ICAM that were not previously described in colon cancer, we performed a comparison of the most frequently mutated genes in AC-ICAM (>5% of the tumor samples) with frequencies detected in previously published datasets containing colon cancer samples (TCGA-COAD and NHS-HPFS) as well as reported cancer driver genes^32^ or colon oncogenic mediators^38^. First, we extracted genes with a nonsynonymous mutation frequency >5% in the AC-ICAM cohort. Subsequently, only genes that are likely involved in cancer development, as described in the section ‘Cancer-related gene annotation’, were retained. All artifact genes (mutations typically encountered as bystander mutations in cancer that are mutated for example as a consequence of a high homology of sequences in the gene), were excluded. Genes that have previously been reported as colon cancer oncogenic mediator^38^ or cancer driver gene for colorectal cancer (COADREAD)^32^ were also excluded. Finally, only genes with a mutation frequency <5% in the NHS-HPFS colon cancer cohort^37^ and <5% in TCGA-COAD^36^ were maintained. As a final filter, only genes that had a nonsynonymous mutation frequency of at least twofold in AC-ICAM compared to TCGA-COAD were labeled as potentially new in colon cancer.

#### Estimation of MSI from whole-exome sequencing data

We applied MANTIS (v.1.0.4), a tool for rapid detection of microsatellite instability on our WES data^77^. Briefly, a bed file suitable for use by MANTIS was created using RepeatFinder function of the MANTIS tool, to find microsatellites regions within the reference genome (GRCh37). MANTIS was then run for each tumor and matched normal BAM file pair using these detected microsatellite loci. The instability score between the two samples within the pair was used to classify samples either as MSI-H (MANTIS score > 0.4) or MSS (MANTIS score ≤ 0.4).

#### Somatic mutations associated with ICR

We investigated the association of specific somatic alterations, including SNVs and small insertions or deletions (indels) and ICR immune phenotype. Binomial linear regression models were fitted to define which specific mutations associate with ICR score using the glm function with family ‘binomial’ (R). This analysis was performed in the total cohort (*n* = 281) as well as within hypermutated (*n* = 69) and non-hypermutated (*n* = 212) subgroups separately. The estimate and *P* value were extracted for each gene and FDR was calculated using the Benjamini–Hochberg method. Significant genes with an FDR < 0.1 and that were mutated in at least five patients in the analysis subgroup were plotted as OncoPrints (ComplexHeatmap, v.2.1.2).

#### Mutations in homologous recombination genes, mucinous histology, and ICR

Genes with an inverse association with ICR score within hypermutated colon cancer included genes involved in homologous recombination repair. The frequency of mutations in either of the identified genes (*BRCA2*, *BRCA1* and *FANCA*) genes were compared between hypermutated cases of mucinous histology with hypermutated cases with other histological classifications. An unpaired Student’s *t*-test was used to compare ICR score between hypermutated cases of mucinous histology with hypermutated cases with other histological classifications.

### Somatic copy-number alteration segmentation

A segmentation file was generated for each sample and later a merged file for all samples was uploaded to IGV (v.2.11.0). We have used a pipeline using GATK (gatk-package-4. beta.6) to generate each tumor sample’s segmentation file. We performed the below steps:

1. Calculated the coverage of tumor and normal BAM files for each interval using GATK CalculateTargetCoverage.
2. Generated the panel of ‘normal’ using normal samples by GATK CreatePanelOfNormals options.
3. Normalizing the tumor data using GATK NormalizeSomaticReadCounts methods using PON generated during the above step.
4. Performed the segmentation of tumor data using input files from the above steps using GATK PerformSegmentation.
5. The merged segmentation file of all the samples was uploaded to IGV and snapshots were generated.

#### Overview of SCNAs

We explored the prevalence of SCNAs among ICR clusters and hypermutated and non-hypermutated subgroups by exploration of the segmentation file in IGV. Briefly, the log_2_-transformed segmentation file was loaded in IGV with reference genome GRCh37, including an annotation text file including mutational load category (hypermutated, non-hypermutated), POLE mutation status, ICR cluster, CMS and MSI status. The samples were ordered consecutively by MSI status, CMS, ICR, POLE and mutational load category. Prevalence of amplification and deletions was visually inspected and compared between groups.

### Genetic immunoediting and immunoediting score

#### HLA typing, neoantigen prediction and GIE

HLA typing was performed on both WES and RNA-seq data using OptiType (bcbio-nextgen v.1.1.5 in Python v.2.7.0)^78^. Neoantigen prediction tool pVACseq from pVACtools was run using the following predictors: MHCnuggetsI, NNalign, NetMHC, SMM, SMMPMBEC and SMMalign. The obtained vcfs from our somatic mutation calling pipeline were used as input for pVACseq, along with the predicted HLA type from WES data. Gene expression data aligned to GRCh37 in transcripts per million was annotated to the vcfs using vcf-expression-annotator. Mutant-specific binders, relevant to the restricted HLA-I allele, are referred to as neoantigens, as described in detail by Zhang et al.^79^. Mutated epitopes with a median IC_50_ binding affinity across all prediction algorithms used <500 nM, with a corresponding wild-type epitope with a median IC_50_ binding affinity > 500 nM, were used as criteria to infer neoantigens. Predicted neoantigens were used to calculate the GIE value. We calculated the GIE value by taking the ratio between the number of observed versus the number of expected neoantigens. The expected number of neoantigens was based on the assumption of a linearity between TMB and the number of neoantigens. We therefore assumed that samples that have a lower frequency of neoantigens than expected (lower GIE values), display evidence of immunoediting. A higher frequency of neoantigens than expected indicates a lack of immunoediting, see calculations section for details.

#### IES classification and analysis

The IES is a composite score based on both ICR and GIE. Tumors of IES4 are those predicted to be the most immune active, as they are ICR high and display GIE. Tumors of IES1 are expected to be most immune silent, classified both as ICR low and an absence of GIE. Tumors of the intermediate groups IES2 and IES3 reflect ICR-low and GIE and ICR-high and non-GIE tumors, respectively. Mutational load category, MSI status and pathological stage distribution was compared between IES groups using a chi-squared test. The OS was compared between patients with different IES and between GIE and non-GIE tumors in the ICR-medium group using Cox proportional hazard regression analysis. A Cox proportional hazard’s multivariate model was fitted with IES (ordinal) and pathological stage (ordinal).

#### Association between IES and TCR clonality

The Spearman correlation between IES as ordinal variable and TCR clonality from immunoSEQ as well as MiXCR-based clonality was calculated. We performed several additional analyses to assess whether the relationship between TCR productive clonality and IES was driven by ICR. Multiple regression analysis was performed with ICR score and immunoSEQ TCR clonality as continuous variables to predict productive TCR clonality (immunoSEQ). Second, the data were modeled through local polynomial regression fitting of the productive TCR clonality (immunoSEQ) by IES category (ordinal variable).

### Microbiome: bacterial 16S rRNA PCR sequencing

This study complies with the STORM reporting guidelines; the completed checklist can be found in Supplementary Table 12.

The 16S rRNA gene sequencing was performed at the Host–microbe Interaction Laboratory, Sidra Medicine.

Hypervariable regions V3–V4 of 16S rRNA gene were amplified with PCR using the amplicon primers with Illumina adaptors (underlined):

Forward:

5′TCGTCGGCAGCGTCAGATGTGTATAAGAGACAGCCTACGGGNGGCWGCAG′3

Reverse:

5′GTCTCGTGGGCTCGGAGATGTGTATAAGAGACAGGACTACHVGGGTATCTAATCC′3.

In brief, PCR was performed in a 25-μl reaction mixture containing 5 μl each forward and reverse primer (1 μM), 2.5 μl template DNA for the samples and 12.5 μl 1× Hot Master Mix (Phusion Hot Start Master Mix). No human DNA depletion was used. The amplifications were performed on a Veriti 96-well Thermal Cycler (Thermo Scientific) with the following program: initial denaturation at 95 °C for 2 min, followed by 30 cycles of denaturation at 95 °C for 30 s, primer annealing at 60 °C for 30 s and extension at 72 °C for 30 s, with a final elongation at 72 °C for 5 min. The presence of PCR products was confirmed by electrophoresis in a 1.5% agarose gel conducted at 80 V/cm in Tris-borate–EDTA (TBE) buffer. Amplicons were then purified using AgenCourt AMPure XP magnetic beads (Beckman Coulter) according to the Illumina MiSeq 16S Metagenomic Sequencing Library Preparation protocol. As positive controls, we included DNA from stool samples (extracted with QIAGEN QIAmp Fast DNA Stool Mini kit), using the same input of DNA as the one used for the AC-ICAM samples. We obtained similar 16S rRNA amplicon PCR products across the tissue samples and the positive controls, indicating that the DNA extraction protocol used resulted in enough recovery of the microbial DNA from our specimens.

Samples were multiplexed using a dual-index approach with the Nextera XT Index kit (Illumina) according to the manufacturer’s instructions. The concentration of amplicons was determined using the Qubit HS dsDNA assay kit (Life Technologies,) followed by pooling to achieve an equimolar library concentration. The final pooled product was paired-end sequenced at 2 × 300 bp using a MiSeq Reagent kit v3 on Illumina MiSeq platform (Illumina) at the Sidra Medicine research facility. Sequencing was also performed on 27 empty wells across plates to exclude the occurrence of large-scale cross-contamination among samples during sequencing procedures: the minimum and maximum read counts were 2 and 234, respectively and the average and median reads counts were 37 and 18, respectively. No negative controls for sampling or DNA extractions were included. Samples were aliquoted randomly in the plate.

### Microbiome: 16S rRNA gene sequencing and data processing

Sequenced data were demultiplexed using MiSeq Control Software. The overall quality of sequencing quality was evaluated using FastQC and the demultiplexed sequencing data were imported into Quantitative Insights into Microbial Ecology (QIIME2; v.2019.4.0) software package. The data were denoised with DADA2, which includes a multi-step process, including read filtering, dereplication and chimera removal. Paired 250-bp reads were trimmed of the initial five low-quality bases and further processed to generate the amplicon sequence variant, interchangeably called operational taxonomic units (OTUs). The data were subsampled at a depth of 22,704 and then normalized using the rarefaction on OTUs count at even depth. Taxonomic classification was performed utilizing 16S rRNA gene database from Silva classifier (silva-132-99-515-806-nb-classifier). The data were imported into R in a Biological Observation Matrix (biom) format, before further evaluation with Phyloseq (v.1.34.0). The 16S rRNA gene sequencing was performed on all samples with sufficient DNA available: 246 tumor samples and 246 matched healthy colon tissues from the same patients (AC-ICAM246) and on additional 42 tumor samples (ICAM42) for which there was no sufficient DNA available from the healthy colon counterpart.

The minimum and maximum read counts were 25,868 and 351,069, respectively. The average and median reads counts were 82,506 and 75,668, respectively. No samples were excluded from the analysis.

Alpha diversity (within sample community) was assessed by observed OTUs (sum of unique OTUs per sample), Chao1 (Chao 1987) an abundance-based richness estimator that is sensitive to rare OTUs, Shannon (Shannon 1948) and inverse Simpson (InvSimpson) (Simpson 1949), the last one being more dependent on highly abundant OTUs and less sensitive to rare OTUs. Indices were read into R using R package vegan (v.2.5–6).

Relative abundance of distinct microbiome elements was determined using the transform_sample_counts function from Phyloseq, such that sum of all abundance values per sample is equal to one (Microbiome_Relative = transform_sample_counts (pyloseq_object, function(x) x / sum(x))). OTU tables were aggregated by taxonomic ranks including phylum (26 unique phyla), class (48 classes), order (97 orders), family (207 families), genus (562 genera) and species (846 species). As the confidence for annotation of reads decreases with decreasing rank, some reads were only annotated with higher ranks.

### Microbiome: WGS and data processing

Library construction and sequencing was performed at the Sidra Clinical Genomics Laboratory Sequencing Facility. DNA was quantified using the Quant-iT dsDNA Assay (Invitrogen) on the FlexStation 3 (Molecular Devices). The library was constructed from 250 ng of DNA with the Illumina TruSeq DNA Nano kit. Library quality and concentration was assessed using the DNA 1k assay on a PerkinElmer GX2 and qPCR using the KAPA Library quantification kit on a Roche LightCycler 480 II. Genomic libraries were sequenced with paired-end 150 bp on HiSeq X (32% of samples) and Novaseq 6000 (68% of samples) systems (Illumina) following the manufacturer’s recommended protocol to achieve a minimum average coverage 60× for tumor samples. Quality passed reads were aligned to the human reference genome GRCh38 using BWA. Human sequencing reads were removed and unaligned nonhost reads were extracted using SAMtools. Low-quality unaligned reads were trimmed and samples were processed for taxonomic profiling using MetaPhlAn2 (ref. ^80^). MetaPhlAn2 uses a library of unique clade-specific marker genes to estimate bacterial relative abundance at the species level. The program was run with default parameters except analysis type set to relative abundance and restricted to bacterial organisms only. WGS was targeted to achieve >60× coverage per sample. The median (across samples) of the average target coverage (per sample) was 76× (range of 50–92).

Of 3.2 × 10^11^ total reads (median 1.9 × 10^9^ reads per sample; IQR 2.1 ×10^8^), 1.5 × 10^8^ (median 1×10^5^ reads per sample; IQR 3.4 × 10^5^) were aligned to bacteria. A total of 132 taxa, at genus level were detected, of which 3 were excluded as possible contaminants (*Deinococcus*, *Ralstonia* and *Enhydrobacter*)^12^ (main matrix). When we applied the same filter as the one used for 16S RNA gene-sequencing data (presence in at least 10% of the samples with at least 1% relative abundance in one sample), 54 taxa at the genus level were retained. WGS was performed in all samples with sufficient DNA available (*n* = 167).

### *Ruminococcusbromii* PCR

PCR was performed based on Wang et al.^81^ using *R.* *bromii* 16S rDNA forward primer (GAAGTAGAGATACATTAGGTG) and *R.* *bromii* 16S rDNA reverse primer (ACGAGGTTGGACTACTGA). PCR was performed using AmpliTaq Gold 360 Master Mix (Thermo Fisher, 4398881), 20 ng of sample DNA and 5 nM of each primer. The amplification conditions were one cycle of 95 °C for 10 min, then 35 cycles of 95 °C for 30 s, 50 °C for 30 s and 72 °C for 30 s and finally one cycle of 72 °C for 7 min before storing at 4 °C. PCR products (10 μl each) were separated by electrophoresis in 2% agarose gels (Sigma, A4718) containing ethidium bromide (1 μg ml^−1^) (Sigma, E1510) using a 100-bp DNA ladder (New England Biolabs, N0551G) for size verification. PCR band intensity was defined as negative when intensity was absent or extremely faint. PCR was considered positive if band was gradually more intense (graded from 2 to 4). PCR was performed in all samples from the AC-ICAM246 cohort with sufficient amounts of DNA available (*n* = 126).

### Microbiome data analysis

#### Genus-level filtering

On tumor samples, microbiome genera were filtered to include genera which are present in at least 10% of the samples with at least 1% relative abundance in one sample; 138 out of 562 were retained. These included 137 genera and the genus labeled ‘unknown’ that reflects all reads for taxa with insufficient confidence at the genus level. The same filtering was applied to normal samples; 129 genera were retained. A total of 120 genera overlapped between normal and tumor samples, 9 genera were unique in normal samples and 18 genera were unique in tumor samples.

This set of filtered genera were used for all downstream analysis except for the comparison between tumor and normal pairs. For this analysis we include any genera that passed the filtering approach described above for either normal or tumor groups (if taxa passed the filtered in tumor samples they were retained in normal samples and vice versa; total 147 genera).

#### Contaminant assessment

To remove putative contaminants from the 16S rRNA gene-sequencing data, we used a list of microbial taxa that are typically found in negative blank reagents, as described by Salter et al.^82^. This list has previously been curated and annotated by Poore et al.^12^ by manual review of the literature. This curation allowed the discrimination of taxa that are ‘likely contaminants’, ‘potentially pathological or commensal genera’ and ‘mixed evidence’ genera that have been described both as pathogens as well as contaminants. We flagged those taxa that were ‘likely contaminants’ as well as ‘mixed evidence’ for potential exclusion from our 16S rRNA gene-sequencing microbiome abundance matrix.

In total, we detected 25 taxa that were ‘likely contaminants’ and 10 taxa with ‘mixed evidence’ in at least one out of the 492 samples. To evaluate the extent of potential contamination by these 35 taxa, we calculated the sum of these taxa for each sample. On average, only 0.04732% of the total microbial abundance per sample consisted of ‘flagged’ taxa (min, 0%; first quartile, 0%; median, 0%; third quartile, 0.03485%; and max, 4.46%). Furthermore, most of these putative contaminant taxa (*n* = 33) were detected in only fewer than 20 (out of 492) samples. Potential contaminating bacteria that we detected in the highest numbers of samples were *Oxalobacter* in 39 samples and *Micrococcus* in 28 samples. Detected putative contaminants and taxa with mixed evidence from the 16S rRNA-sequencing data were removed when we applied the minimal abundance filter (presence in at least 10% of the samples with at least 1% relative abundance in one sample).

#### Microbiome comparison between tumor and healthy colon tissue

At the phylum level, the overall distribution of microbiome composition was visualized using stacked bar charts. The order of samples was determined by descending relative abundance of the phylum *Fusobacteria* in tumor samples and the matching healthy colon samples from corresponding patients were ranked in the same order as the tumor stacked bar chart.

A paired Mann–Whitney *U*-test (two-sided) was used to determine microbial phyla/genera with significantly different relative abundance between tumor and paired normal samples. FDR was calculated using the Benjamini–Hochberg method. Results were visualized in volcano plots.

#### Microbiome comparison between ICR groups

An unpaired Mann–Whitney *U*-test (two-sided) was used to calculate which filtered genera (*n* = 138) were differentially abundant between ICR-high and ICR-low samples. FDR was calculated using the Benjamini–Hochberg method. Results were visualized in volcano plots.

#### Co-abundance network inference

We performed co-abundance analysis in tumor samples from the AC-ICAM246 cohort. Co-abundance analysis, which involves studying the presence of multiple components within a composition, can be difficult to perform accurately when using relative abundance. This is because the relative abundance of the different components is constrained to sum to 1, which can lead to the appearance of false correlations. To address this issue, techniques such as co-abundance network inference can be used to more accurately infer relationships between the components.

Before co-occurrence analysis, the genus labeled ‘unknown’ was excluded. SparCC^48,83^ was used to calculate the co-occurrences between the 137 remaining taxa using centered log-ratio (clr)-transformed OTU counts in tumor samples (Python, SparCC3). A total of 500 inference and 10 exclusion iterations were used to estimate the median correlation of each pairwise. The statistical significance of the correlations was calculated using a bootstrapping procedure to generate 500 simulated data^83^. For each component pair, pseudo *P* values (two-sided) were assigned as the proportion of simulated bootstrapped data with a correlation at least as extreme as the one computed for the original data. Benjamini–Hochberg FDR was used for multiple testing correction. All the correlations were then sorted using a statistically significant cutoff (FDR < 0.05) and SparCC correlation coefficient > ±0.3. Clusters among the networks (groups of at least three correlated genera using the cutoffs specified above) were defined via a fast greedy clustering algorithm. All co-occurrence networks were made using the R package ‘NetCoMI (v.1.1.0) – Network Construction and Comparison for Microbiome Data’^84^ and visualized using Cytoscape (v.3.9.1).

Within each cluster, the total relative abundance was calculated by summing up the relative abundance values for genera that positively correlated with each other. For each of the identified clusters, survival analysis was performed by binarizing each sample into high and low abundance based on the median total relative abundance of each cluster.

#### MBR model development, training set

We first normalized the genus abundance matrix by converting each genus column into a *z* score using mean and s.d. and treating the normalized abundance matrix as the training set. We built a relaxed multivariable elastic-net OS Cox regression model using the glmnet R package (v.4.1.4) on the training set. The optimal hyper parameters (γ and λ) for the best model were identified through fivefold cross-validation via a grid-search technique using the ‘cv.glmnet’ function. We used the concordance index as a performance metric. The parameters for which the mean cross-validation concordance index was the highest were selected as optimal hyper parameters. Next, the final model was built using these hyper parameters on the complete training set. To calculate risk scores in the training dataset (MBR scores), we passed the training set and best model to the ‘predict’ function. A total of 41 features (genera) were present in the best model with nonzero coefficients; we refer to these features as the ‘MBR classifier’, which represents the final model. A positive or negative coefficient of each genus of the MBR classifier can be binarized into ‘high-risk’ and ‘low-risk’ groups using the cutoff threshold of 0 and attributed to the strength of association with survival. A higher positive coefficient means high hazard risk of death, whereas a negative coefficient corresponds to lower risk of death.

#### MBR model validation, testing sets

We validated the final model on two datasets. Both datasets consist of samples that were not used for model training (unseen data). One is an independent internal (ICAM42) dataset, referred to as testing cohort 1 and the other is an external cohort (TCGA-COAD), referred to as testing cohort 2. The ICAM42 consists of 42 samples and TCGA-COAD consists of 117 samples. We processed the two datasets to convert the abundance values for each genus into *z* scores using the mean and s.d. derived from the training set. These abundance matrices were passed to the ‘predict’ function along with the best model to estimate corresponding risk scores. The risk score (MBR score) of any tested sample is only dependent on the relative abundance of the list of genera that overlap with the ones included in the MBR classifier (the risk score for each sample is not dependent on one of the other samples). Finally, the MBR scores are binarized using the cutoff threshold 0 to categorize the test sample into ‘high-risk’ (>0) and ‘low-risk’ (<0) groups as performed in the training set. Therefore, no cutoff optimization occurred in the validation phase.

#### MBR model performance assessment

We tested the concordance index (1) in the training set using the final MBR model; (2) in the training set using the cross-validation of the best MBR model (five permutations, 80% training and 20% validation partition); and (3) in each test set cohort separately (ICAM42 and TCGA-COAD) and in the full test set (ICAM42 and TCGA-COAD combined) using the final MBR model.

#### Taxa used for the MBR score calculation in other cohorts

To calculate the MBR score in each additional dataset we used taxa that overlapped with the 41 genera of the MBR classifier, which was developed using 16S rRNA gene sequencing on tumor samples.

There were 16 of 41 taxa in the TCGA-COAD (WGS data) and 18 of 41 taxa in the AC-ICAM WGS data (tumor sample) main matrices. All the 41 taxa were available in the ICAM42 cohort (tumor samples) and the MBR score for AC-ICAM healthy colon tissue samples was based on 36 genera that passed the applied genus-level filtering for healthy tissue (the list of the taxa used for each platform is available in Supplementary Table 11).

The Silva classifier used for genus attribution in the 16S rRNA gene-sequencing data includes ‘*Ruminococcus 1*’ and ‘*Ruminococcus 2*’, whereas WGS-WES TCGA data only include ‘*Ruminococcus*’ as genus-level taxa. Therefore, for matching purposes, when calculating the risk score, we replaced the labeling of ‘*Ruminococcus 1*’ and ‘*Ruminococcus 2*’ with ‘*Ruminococcus*’. In WGS AC-ICAM ‘*R.* *bromii*’ was used instead.

#### *R.bromii* validation analysis

We characterized the specific species underlying the reads supporting the *Ruminococcus 2* taxon from 16S sequencing data. Previously, a high degree of sequence similarity was reported between the *Ruminococcus 2* taxa from the Silva classifier and the species *R.* *bromii*^85^. The subset of samples that had both 16S sequencing and WGS data available was used to calculate the Spearman correlation between each *Ruminococcus* species (from WGS data) and the 16S *Ruminococcus 2* (16S) relative abundance. In addition, the proportion of WGS reads that mapped to each specific *Ruminococcus* species was calculated as fraction of all WGS reads that were assigned to the *Ruminococcus* genus.

To confirm the presence of *R.* *bromii*, we performed a PCR specific to *R.* *bromii* on the 126 AC-ICAM tumor samples for which sufficient DNA was still available (see section *R.* *bromii* PCR for technical details on PCR). The concordance between detection of *R.* *bromii* in PCR and 16S *Ruminococcus 2* was defined as the percentage of samples for which both methods had identical results. The discordant cases were further examined by evaluation of WGS results. Furthermore, the concordance between detection of *R.* *bromii* in PCR and *R.* *bromii* in WGS was assessed in the 86 samples for which data from both methods were available.

#### mICRoScore development

In view of the individual contribution of analytes extrapolated by individual platforms such as the ICR (RNA-seq data), the GIE (WES data) and the MBR scores (16S data) and TCR clonality (immunoSEQ and MiXCR) we sought to develop a multi-omics parameter that could capture a subgroup of patients with exceptional survival.

Each parameter that was significant in the univariate Cox regression analysis (ICR, as ordinal variable, low, medium, high; GIE as binary variable, non-GIE versus GIE; and MBR score, as binary variable, low versus high), was assessed within a multivariable Cox regression model adjusted for age (as continuous variable), CMS subtypes (as categorical variable, CMS1–CMS3, versus CMS4), stage (as ordinal variable, I, II, III and IV) and MSI status (as binary variable, MSS versus MSI-H). The parameters that were retained by the multivariable Cox models were combined into an integrated score. For univariate analysis we used RNA-seq, WES and TCR clonality data from the entire AC-ICAM cohort and MBR score derived from 16S rRNA gene-sequencing data of the AC-ICAM246 cohort.

The mICRoScore reflects the co-presence of ICR high and MBR low risk, defined as mICRoScore high. On the AC-ICAM246 (training set), all samples with MBR-high risk and/or in ICR-medium or ICR-low group are defined as mICRoScore low. The survival between patients with mICRoScore high and mICRoScore low was compared using Cox proportional hazard regression analysis and a log-rank test.

#### mICRoScore validation

We used data from TCGA-COAD as external validation cohort to test the mICRoScore (testing set). The TCGA-COAD cohort includes 107 patients with both tumor microbiome data (downloaded from Dohlman et al.^10^) and RNA-seq data available (used for ICR estimation). ICR assignments from this cohort (see section TCGA data) were combined with the MBR classification to classify patients as mICRoScore high and mICRoScore low. The survival between patients with mICRoScore high and mICRoScore low was compared using a log-rank test.

### Sample size considerations

Sample size calculation is challenging in multi-omics studies due to the multitude of parameters that could be examined (implying the use of different tests from different platforms generating data with different data distribution) and empirical methods have been used by many consortia. Correlation between ICR and survival was declared as a primary objective in the research proposal submitted to the funding agency before any genomic data were generated, representing therefore a prospective–retrospective validation (JSREP07-010-3-005).

In the submitted proposal (2015), we planned to profile 400 tumors for gene expression analysis (samples from 456 patients were screened, samples from 391 patients were available for processing and samples from 348 patients retained after QC in the final cohort, see Extended Data Fig. 1) and at least 100 tumor–normal pairs for WES analysis (initially planned only for a subgroup of ICR-high versus -low tumors) and 100 for TCR sequencing using the immunoSEQ assay considering the high amount of DNA that is necessary (>2 μg). Securing additional funds allowed us to perform WGS and 16S rRNA sequencing and to expand the WES and TCR analyses to any sample with sufficient DNA available. No specific power calculation was performed at that time and the targeted sample size was based on the estimated number of samples that could be retrieved from LUMC (*n* = 400), which compared favorably with the sample size of similar studies in the field.

Regarding detection of somatic mutations and considering the overall somatic mutations frequency in colon cancer, 150 tumor exomes will give a power >90% to detect a 10% mutational frequency in 90% of genes^86^.

Regarding survival analysis, in terms of ICR (the primary objective in the submitted proposal), for the comparison between ICR high versus ICR low, with 77 OS events detected, our study has a power >80% for an HR of 0.5 with a two-sided α of 0.05. With 154 OS events in the whole cohort, our study has a power of 90% for an HR of 0.59 (assuming two group of equal size c) and a power of 90% for an HR of 0.57 (assuming groups with unequal sample size, 2:1) with a two-sided α of 0.05.

### Calculations

#### TCR clonality calculation by immunoSEQ assay data (targeted DNA)

Entropy (*H*) is calculated by a standard Shannon entropy calculation with log base 2. Clonality is the inverse of the normalized entropy calculation. The equations are below:

$${{{\mathrm{Shannon}}}}\;{{{\mathrm{entropy:}}}}\;{{{{H}}}}\left( {{{\mathrm{x}}}} \right){{{\mathrm{ = - }}}}\Sigma {{{{P}}}}\left( {{{\mathrm{x}}}} \right){{{\mathrm{log}}}}_{2}\left[ {{{{{P}}}}\left( {{{\mathrm{x}}}} \right)} \right]$$

Specifically, for our data:

For a productive (inframe) sequence x,

$${{{{P}}}}\left( {{{\mathrm{x}}}} \right) = {{{\mathrm{sequence}}}}\;{{{\mathrm{count}}}}/{{{\mathrm{total}}}}\;{{{\mathrm{productive}}}}\;{{{\mathrm{count}}}}$$

$$\begin{array}{l}{{{\mathrm{Entropy}}}}=\\ {{{\mathrm{ - 1}}}}\; \times \;{{{\mathrm{the}}}}\;{{{\mathrm{sum}}}}\;{{{\mathrm{over}}}}\;{{{\mathrm{all}}}}\;{{{\mathrm{unique}}}}\;{{{\mathrm{productive}}}}\;\left( {{{{\mathrm{inframe}}}}} \right)\;{{{\mathrm{sequences}}}}\;{{{\mathrm{of}}}}\\ \left(\right. \left( {{{{\mathrm{sequence}}}}\;{{{\mathrm{count}}}}/{{{\mathrm{total}}}}\;{{{\mathrm{productive}}}}\;{{{\mathrm{count}}}}} \right)\\ \times {{{\mathrm{log}}}}_{2}\left( {{{{\mathrm{sequence}}}}\;{{{\mathrm{count}}}}/{{{\mathrm{total}}}}\;{{{\mathrm{productive}}}}\;{{{\mathrm{count}}}}} \right) \left.\right)\end{array}$$

$$\begin{array}{l}{{{\mathrm{Normalized}}}}\;{{{\mathrm{entropy}}}} =\\{{{\mathrm{entropy}}}}/{{{\mathrm{log}}}}_{2}\left( {{{{\mathrm{productive}}}}\;{{{\mathrm{unique}}}}\;{{{\mathrm{inframe}}}}\;{{{\mathrm{sequences}}}}} \right)\end{array}$$

$${{{\mathrm{Clonality}}}} = 1 - {{{\mathrm{normalized}}}}\;{{{\mathrm{entropy}}}}$$

#### TCR clonality calculation on bulk RNA-seq data (MiXCR)

Entropy (*H*) is calculated by a standard Shannon entropy calculation with log base 2. The equations are below:

$${{{\mathrm{Shannon}}}}\;{{{\mathrm{entropy}}}\;{{{{H}}}}}\left( {{{\mathrm{x}}}} \right){{{\mathrm{ = - }}}}\Sigma {{{{P}}}}\left( {{{\mathrm{x}}}} \right){{{\mathrm{log}}}}_2\left( {{{{{P}}}}\left( {{{\mathrm{x}}}} \right)} \right)$$

For a sequence x,

$${{{{P}}}}\left( {{{\mathrm{x}}}} \right){{{\mathrm{ = sequence}}}}\;{{{\mathrm{count}}}}/{{{\mathrm{total}}}}\;{{{\mathrm{count}}}}$$

The Shannon entropy was normalized so that it can assume a value between 0 and 1. The normalized Shannon entropy is referred to as Pielou’s evenness and is calculated as below:

$${\mathrm{Pielou}}\hbox{'}{\mathrm{s}}\;{{{\mathrm{evenness:}}}\;{{{{J = H}}}}/{{{\mathrm{log}}}}}\left( {{{{S}}}} \right)$$

where *S* is the number of unique TCR/CDR3 sequences.

Clonality is calculated as the inverse of the normalized entropy (*J*) calculation:

$${{{\mathrm{Clonality = 1 - }{J}}}}$$

#### Genetic immunoediting value

The GIE value is calculated by taking the ratio between the observed (O) versus the expected (E) number of neoantigens:

$${{{\mathrm{GIE}}}}\;{{{\mathrm{value = O/E}}}}$$

in which E is a function of the number of nonsynonymous mutations in that specific sample (x):

$${{{\mathrm{E}}}}\left( {{{\mathrm{x}}}} \right){{{\mathrm{ = - 2}}}}{{{\mathrm{.38770 + 0}}}}{{{\mathrm{.09171}}}} \times {{{\mathrm{x}}}}$$

### Reporting summary

Further information on research design is available in the Nature Portfolio Reporting Summary linked to this article.

## Online content

Any methods, additional references, Nature Portfolio reporting summaries, source data, extended data, supplementary information, acknowledgements, peer review information; details of author contributions and competing interests; and statements of data and code availability are available at 10.1038/s41591-023-02324-5.

### Supplementary information

Supplementary Information Supplementary Figs. 1–14, Supplementary Table 1 and Supplementary Table 12 (STORMS checklist).

Reporting Summary

Supplementary Tables 2–11 All Supplementary Tables merged into a single Excel file, including separate sheets.

Supplementary Source Data Data are used across main figures and Extended Data Figs. 1–10 and Supplementary Figs. 1–12. A complete list of all the Source Data is available on sheet 1 of the Supplementary Data workbook, followed by a source data figure location in sheet 2.

### Source data

Source Data Extended Data Fig. 10 PDF file of the raw PCR blots seen in Extended Data Fig.10b.

## Data Availability

BAM files for RNA and WES data, along with FastQ files for 16S rDNA sequencing and non-aligned WGS reads are available through controlled access at dbGaP (phs002978.v1.p1) and public access SRA (PRJNA941834; 16S and WGS). Names of the raw data files contain barcodes with a fixed structure as follows: Example barcode: SER-SILU-CC-P0001-PT-01-A-01 -Study category: SER (Sidra Extrant Research) -Study: SILU (Sidra-LUMC) -Cancer type: CC (Colon Cancer) -Patient ID: P0001 (P for patient followed by four-digit number) -Sample: PT (primary tumor), AN (adjacent normal) -Portion: 01, 02, 03 (in case of multiple PT from same patient) -Assay + pipeline: A-01: RNA-seq, GRCh38 (used for gene expression) A-02: RNA-seq, GRCh37 (used for MiXCR and neoantigen prediction) B-02: WES, GRCh37 C-01: TCRSeq, Adaptive pipeline D-01: 16S rRNA gene sequencing D-02: WGS unaligned nonhost reads Source data for all main figures, Extended Data Figs. 1–10 and Supplementary Figs. 1–12 are available as Supplementary Data. The Supplementary Data workbook includes per-sample metrics from RNA-seq, WES, TCR immunoSEQ and microbiome profiling. A complete list of Source Data is available on sheet 1 of the Supplementary Data workbook, followed by source data figure location in sheet 2. A secondary repository for Supplementary Data is available via FigShare (10.6084/m9.figshare.16944775)^87^, including large files such as the MAF files for WES, segmentation file for the analysis of copy-number genomic aberrations, the 16S OTU tables. FigShare will be also updated with metrics that will be generated in the future. Processed data and clinical data are also available via cBioportal for interactive data exploration (Sidra-LUMC AC-ICAM dataset; https://www.cbioportal.org/). Access to SRA, cBioportal and FigShare is unrestricted and immediate, controlled access through dbGAP is managed by the National Institutes of Health/National Cancer Institute data access committee through the dbGAP portal. An estimation of the required time to obtain access to the data and detailed statistics on the outcome and timeline of the data access request can be found at https://www.ncbi.nlm.nih.gov/projects/gap/cgi-bin/DataUseSummary.cgi. Source data are provided with this paper.

## Code availability

Scripts and command lines used to analyze the sequencing and genomic data are available at GitHub AC-ICAM-NM, including the script used for the development of the MBR model and calculation of the MBR risk score (10.5281/zenodo.7766220).

## References

[1] National Comprehensive Cancer Network. NCCN Clinical Practice Guidelines in Oncology https://www.nccn.org/guidelines/category_1 (2023).

[2] Argilés G (2020). Localised colon cancer: ESMO Clinical Practice Guidelines for diagnosis, treatment and follow-up. Ann. Oncol..

[3] Sayaman RW (2021). Germline genetic contribution to the immune landscape of cancer. Immunity.

[4] Pagès F (2018). International validation of the consensus Immunoscore for the classification of colon cancer: a prognostic and accuracy study. Lancet.

[5] Mlecnik B (2016). Integrative analyses of colorectal cancer show immunoscore is a stronger predictor of patient survival than microsatellite instability. Immunity.

[6] Bruni D, Angell HK, Galon J (2020). The immune contexture and Immunoscore in cancer prognosis and therapeutic efficacy. Nat. Rev. Cancer.

[7] Foersch, S. et al. Multistain deep learning for prediction of prognosis and therapy response in colorectal cancer. *Nat. Med*. 10.1038/s41591-022-02134-1 (2023). 10.1038/s41591-022-02134-1 36624314

[8] Iglesia MD (2016). Genomic analysis of immune cell infiltrates across 11 tumor types. J. Natl Cancer Inst..

[9] Danaher P (2018). Pan-cancer adaptive immune resistance as defined by the Tumor Inflammation Signature (TIS): results from The Cancer Genome Atlas (TCGA). J. Immunother. Cancer.

[10] Roelands J (2020). Oncogenic states dictate the prognostic and predictive connotations of intratumoral immune response. J. Immunother. Cancer.

[11] Liu J (2018). An integrated TCGA pan-cancer clinical data resource to drive high-quality survival outcome analytics. Cell.

[12] Poore GD (2020). Microbiome analyses of blood and tissues suggest cancer diagnostic approach. Nature.

[13] Dohlman AB (2021). The cancer microbiome atlas: a pan-cancer comparative analysis to distinguish tissue-resident microbiota from contaminants. Cell Host Microbe.

[14] Aran D, Sirota M, Butte AJ (2015). Systematic pan-cancer analysis of tumour purity. Nat. Commun..

[15] Bindea G (2013). Spatiotemporal dynamics of intratumoral immune cells reveal the immune landscape in human cancer. Immunity.

[16] Guinney J (2015). The Consensus Molecular Subtypes of colorectal cancer. Nat. Med..

[17] Mima K (2016). *Fusobacterium nucleatum* in colorectal carcinoma tissue and patient prognosis. Gut.

[18] Wang E, Worschech A, Marincola FM (2008). The Immunologic Constant of Rejection. Trends Immunol..

[19] Galon J, Angell HK, Bedognetti D, Marincola FM (2013). The continuum of cancer immunosurveillance: prognostic, predictive, and mechanistic signatures. Immunity.

[20] Bertucci F (2018). The Immunologic Constant of Rejection classification refines the prognostic value of conventional prognostic signatures in breast cancer. Br. J. Cancer.

[21] Hendrickx W (2017). Identification of genetic determinants of breast cancer immune phenotypes by integrative genome-scale analysis. Oncoimmunology.

[22] Sherif S (2022). The immune landscape of solid pediatric tumors. J. Exp. Clin. Cancer Res..

[23] Bertucci F (2022). Immunologic Constant of Rejection signature is prognostic in soft-tissue sarcoma and refines the CINSARC signature. J. Immunother. Cancer.

[24] Rozenblit M (2019). Transcriptomic profiles conducive to immune-mediated tumor rejection in human breast cancer skin metastases treated with Imiquimod. Sci. Rep..

[25] Mason, M. et al. A community challenge to predict clinical outcomes after immune checkpoint blockade in non-small cell lung cancer. Preprint at *bioRxiv*10.1101/2022.12.05.518667 (2022). 10.1186/s12967-023-04705-3 PMC10880244 38383458

[26] Roelands J (2017). Immunogenomic classification of colorectal cancer and therapeutic implications. Int. J. Mol. Sci..

[27] Schumacher TN, Scheper W (2016). A liquid biopsy for cancer immunotherapy. Nat. Med.

[28] Simoni Y (2018). Bystander CD8^+^ T cells are abundant and phenotypically distinct in human tumour infiltrates. Nature.

[29] Scheper W (2019). Low and variable tumor reactivity of the intratumoral TCR repertoire in human cancers. Nat. Med..

[30] Bolotin DA (2015). MiXCR: software for comprehensive adaptive immunity profiling. Nat. Methods.

[31] van der Leun AM, Thommen DS, Schumacher TN (2020). CD8^+^ T cell states in human cancer: insights from single-cell analysis. Nat. Rev. Cancer.

[32] Bailey MH (2018). Comprehensive characterization of cancer driver genes and mutations. Cell.

[33] Zhang J (2015). Germline mutations in predisposition genes in pediatric cancer. N. Engl. J. Med..

[34] Gröbner SN (2018). The landscape of genomic alterations across childhood cancers. Nature.

[35] Saad M (2022). Genetic predisposition to cancer across people of different ancestries in Qatar: a population-based, cohort study. Lancet Oncol..

[36] Ellrott K (2018). Scalable open science approach for mutation calling of tumor exomes using multiple genomic pipelines. Cell Syst..

[37] Giannakis M (2016). Genomic correlates of immune-cell infiltrates in colorectal carcinoma. Cell Rep..

[38] Colaprico A (2020). Interpreting pathways to discover cancer driver genes with Moonlight. Nat. Commun..

[39] Harpaz N (2020). Mucinous histology, BRCA1/2 mutations, and elevated tumor mutational burden in colorectal cancer. J. Oncol..

[40] Muzny DM (2012). Comprehensive molecular characterization of human colon and rectal cancer. Nature.

[41] Angelova M (2018). Evolution of metastases in space and time under immune selection. Cell.

[42] Kostic AD (2012). Genomic analysis identifies association of *Fusobacterium* with colorectal carcinoma. Genome Res..

[43] Wei Z (2016). Could gut microbiota serve as prognostic biomarker associated with colorectal cancer patients’ survival? A pilot study on relevant mechanism. Oncotarget.

[44] Mima K (2015). *Fusobacterium nucleatum* and T cells in colorectal carcinoma. JAMA Oncol..

[45] Gur C (2015). Binding of the Fap2 protein of *Fusobacterium nucleatum* to human inhibitory receptor TIGIT protects tumors from immune cell attack. Immunity.

[46] Gur C (2019). *Fusobacterium nucleatum* supresses anti-tumor immunity by activating CEACAM1. Oncoimmunology.

[47] Udayasuryan B (2022). *Fusobacterium nucleatum* induces proliferation and migration in pancreatic cancer cells through host autocrine and paracrine signaling. Sci. Signal..

[48] Friedman J, Alm EJ (2012). Inferring correlation networks from genomic survey data. PLoS Comput. Biol..

[49] Broz ML (2014). Dissecting the tumor myeloid compartment reveals rare activating antigen-presenting cells critical for T cell immunity. Cancer Cell.

[50] Helmink BA, Khan MAW, Hermann A, Gopalakrishnan V, Wargo JA (2019). The microbiome, cancer, and cancer therapy. Nat. Med..

[51] Nejman D (2020). The human tumor microbiome is composed of tumor type-specific intracellular bacteria. Science.

[52] Smith M (2022). Gut microbiome correlates of response and toxicity following anti-CD19 CAR T cell therapy. Nat. Med..

[53] Gopalakrishnan V (2017). Gut microbiome modulates response to anti-PD-1 immunotherapy in melanoma patients. Science.

[54] Liang H (2022). Predicting cancer immunotherapy response from gut microbiomes using machine learning models. Oncotarget.

[55] Routy B (2018). Gut microbiome influences efficacy of PD-1-based immunotherapy against epithelial tumors. Science.

[56] Spencer CN (2021). Dietary fiber and probiotics influence the gut microbiome and melanoma immunotherapy response. Science.

[57] Simpson RC (2022). Diet-driven microbial ecology underpins associations between cancer immunotherapy outcomes and the gut microbiome. Nat. Med.

[58] Chalabi M (2020). Neoadjuvant immunotherapy leads to pathological responses in MMR-proficient and MMR-deficient early-stage colon cancers. Nat. Med..

[59] Messaoudene M (2022). A natural polyphenol exerts antitumor activity and circumvents anti-PD-1 resistance through effects on the gut microbiota. Cancer Discov..

[60] Liu L (2018). Breast cancer stem cells characterized by CD70 expression preferentially metastasize to the lungs. Breast Cancer.

[61] Galeano Niño JL (2022). Effect of the intratumoral microbiota on spatial and cellular heterogeneity in cancer. Nature.

[62] Noviello, T. M. R. et al. Guadecitabine plus ipilimumab in unresectable melanoma: five-year follow-up and correlation with integrated, multiomic analysis in the NIBIT-M4 trial. Preprint at *medRxiv*10.1101/2023.02.09.23285227 (2023).

[63] Łuksza M (2022). Neoantigen quality predicts immunoediting in survivors of pancreatic cancer. Nature.

[64] Zapata L (2023). Immune selection determines tumor antigenicity and influences response to checkpoint inhibitors. Nat. Genet..

[65] Li H, Durbin R (2010). Fast and accurate long-read alignment with Burrows–Wheeler transform. Bioinformatics.

[66] Huang K (2018). Pathogenic germline variants in 10,389 adult cancers. Cell.

[67] Vogelstein B (2013). Cancer genome landscapes. Science.

[68] Chakravarty D (2017). OncoKB: a precision oncology knowledge base. JCO Precis. Oncol.

[69] Wilkerson MD, Hayes DN (2010). ConsensusClusterPlus: a class discovery tool with confidence assessments and item tracking. Bioinformatics.

[70] Jiménez-Sánchez A, Cast O, Miller ML (2019). Comprehensive benchmarking and integration of tumor microenvironment cell estimation. Methods Cancer Res..

[71] Barbie DA (2009). Systematic RNA interference reveals that oncogenic KRAS-driven cancers require TBK1. Nature.

[72] Thorsson V (2018). The immune landscape of cancer. Immunity.

[73] Sayaman RW (2022). Analytic pipelines to assess the relationship between immune response and germline genetics in human tumors. STAR Protoc..

[74] Benci JL (2019). Opposing functions of interferon coordinate adaptive and innate immune responses to cancer immune checkpoint blockade. Cell.

[75] Beausang JF (2017). T cell receptor sequencing of early-stage breast cancer tumors identifies altered clonal structure of the T cell repertoire. Proc. Natl Acad. Sci. USA.

[76] D’Angelo F (2019). The molecular landscape of glioma in patients with neurofibromatosis 1. Nat. Med..

[77] Bonneville R (2017). Landscape of microsatellite instability across 39 cancer types. JCO Precis. Oncol..

[78] Szolek A (2014). OptiType: precision HLA typing from next-generation sequencing data. Bioinformatics.

[79] Zhang J (2019). The combination of neoantigen quality and T lymphocyte infiltrates identifies glioblastomas with the longest survival. Commun. Biol..

[80] Truong DT (2015). MetaPhlAn2 for enhanced metagenomic taxonomic profiling. Nat. Methods.

[81] Wang R-F, Cao W-W, Cerniglia CE (1997). PCR detection of *Ruminococcus* spp. in human and animal faecal samples. Mol. Cell. Probes.

[82] Salter SJ (2014). Reagent and laboratory contamination can critically impact sequence-based microbiome analyses. BMC Biol..

[83] Weiss S (2016). Correlation detection strategies in microbial data sets vary widely in sensitivity and precision. ISME J..

[84] Peschel S, Müller CL, von Mutius E, Boulesteix A-L, Depner M (2021). NetCoMi: network construction and comparison for microbiome data in R. Brief. Bioinform..

[85] Henderson G (2019). Improved taxonomic assignment of rumen bacterial 16S rRNA sequences using a revised SILVA taxonomic framework. PeerJ.

[86] Spratt DE (2016). Racial/ethnic disparities in genomic sequencing. JAMA Oncol..

[87] Roelands, J. et al. Supplementary Data AC-ICAM. *Figshare*10.6084/m9.figshare.16944775.v1 (2023).

