# Supplementary material for: An integrated tumor, immune and microbiome atlas of colon cancer
Source: Nat Med. 2023 May 19;29(5):1273–86. doi: 10.1038/s41591-023-02324-5 (PMC10202816; doi:10.1038/s41591-023-02324-5)
Supplement: Supplementary file 1 — Supplementary Figs. 1–14, Supplementary Table 1 and Supplementary Table 12 (STORMS checklist). [file 41591_2023_2324_MOESM1_ESM.pdf]

nature medicine

Resource

https://doi.org/10.1038/s41591-023-02324-5

An integrated tumor, immune and microbiome atlas of colon cancer

In the format provided by the authors and unedited

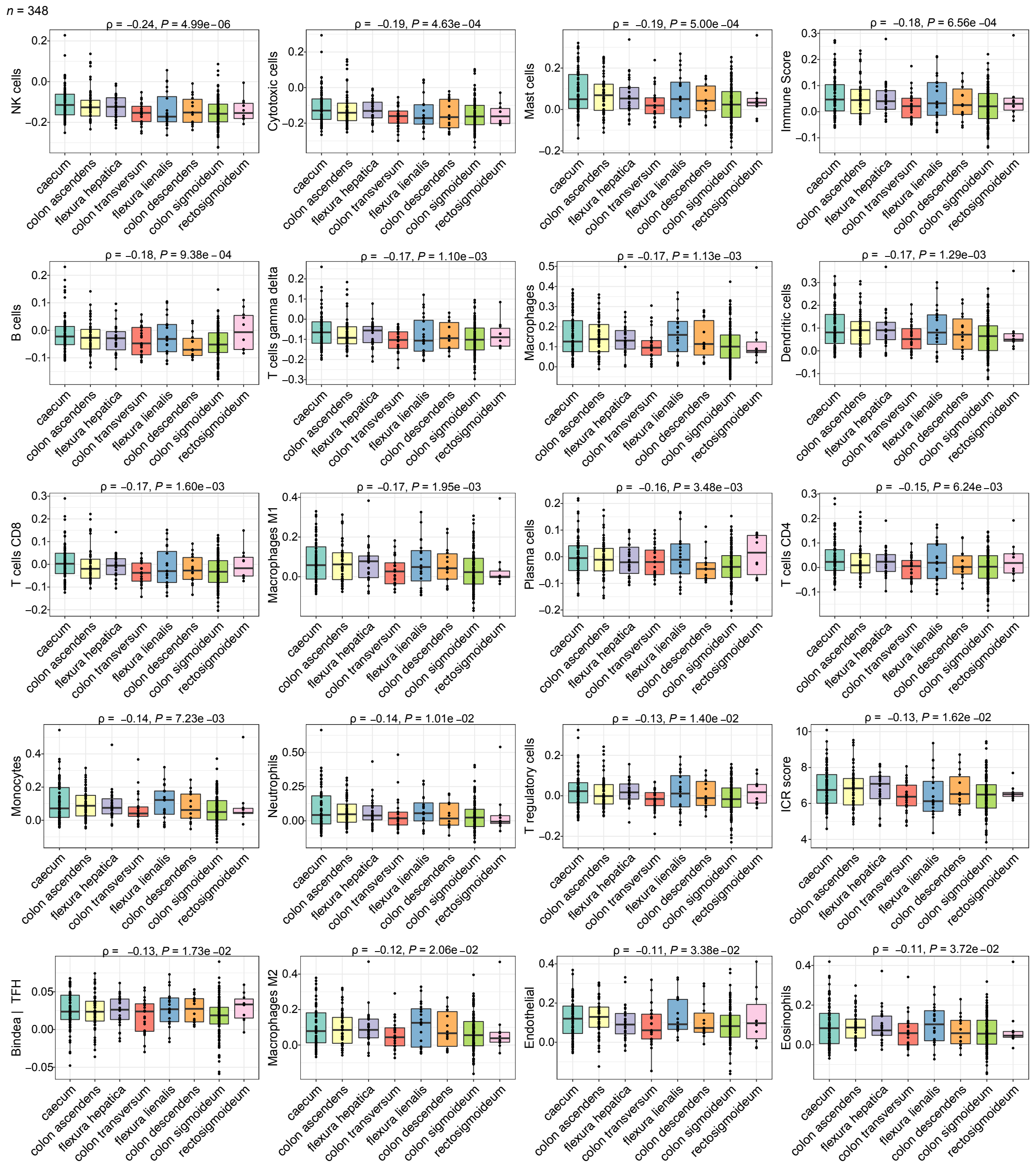

Supplementary Figure 1. ConsensusTME signatures, follicular helper T cells (TFH) and ICR score by anatomical location. ConsensusTME enrichment scores, Follicular T helper signature (TFH) from Bindea et al, 2013, and ICR score by anatomical location of the primary colon tumor. Spearman correlation coefficient (ρ) (two-sided) and corresponding P value are indicated. For all signatures FDR < 0.1. Center line, box limits, and whiskers represent the median, interquartile range and 1.5x interquartile range respectively. *n* = 348 independent samples from individual patients. All *P* values are two-sided; *n* reflects the independent number of samples in all panels.

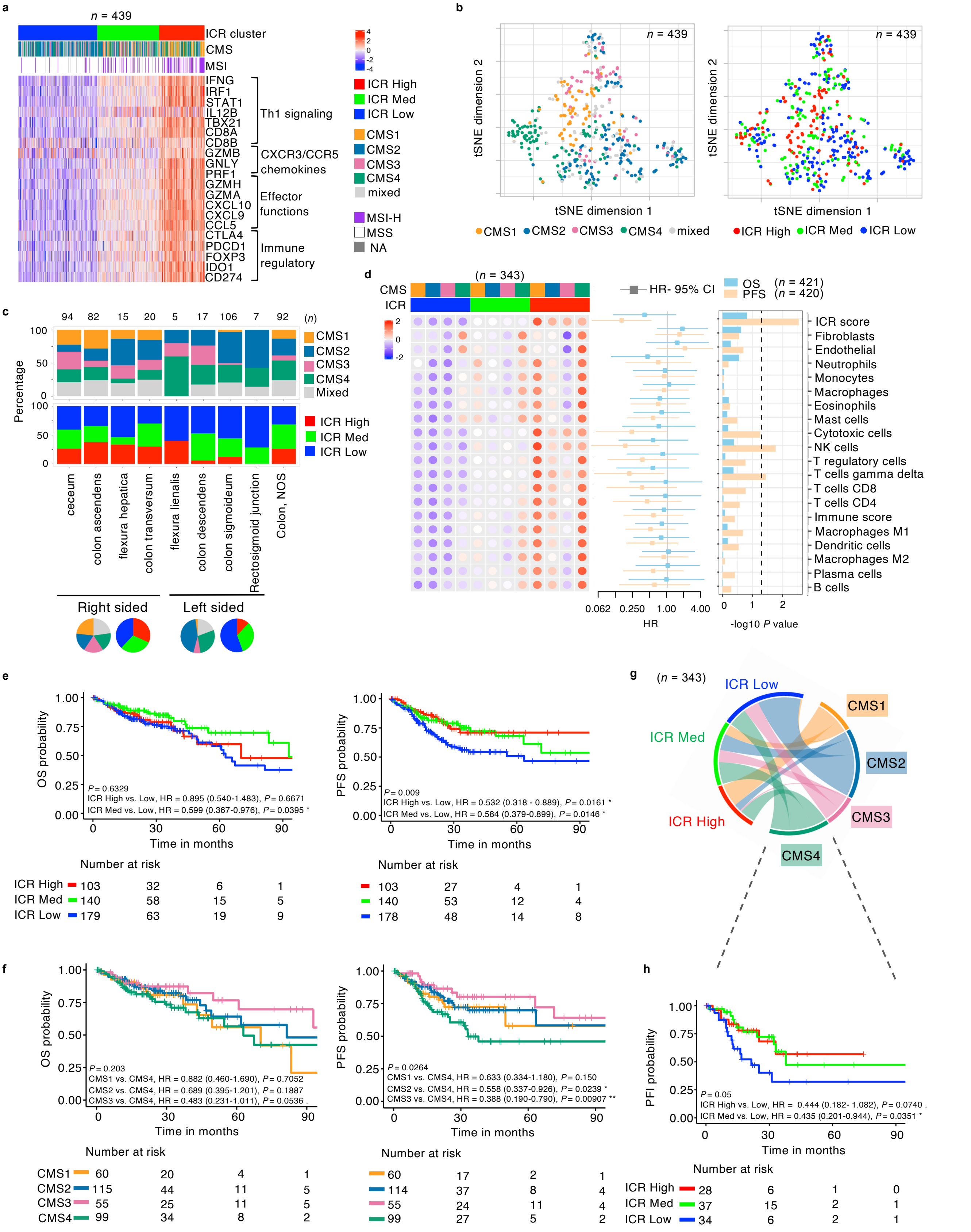

Supplementary Figure 2. Prognostic implications of immune-related gene signatures in TCGA-COAD. a, Heatmap of 20 ICR genes (normalized, log2 transformed expression values, z-scored by row) by sample. **b**, First and second dimension from t-distributed stochastic neighbor embedding (tSNE) dimension reduction algorithm applied to whole transcriptome data of colon tumor samples annotated by CMS (left) and ICR cluster (right). **c**, Stacked bar chart showing proportion of CMS by anatomic location of the tumor. Pie charts reflect the proportions within right sided (ceceum until colon transversum) and left sided tumors (flexura lienalis until rectosigmoid junction).

Supplementary Figure 2 (cont). d, Deconvoluted abundancies of distinct infiltrating cell populations by implementation of consensusTME and their association with OS and PFS. Median enrichment scores (z-scored by row) within each CMS, stratified by ICR cluster are indicated in the dotted heatmap (left). HRs (centre), corresponding 95% confidence intervals (error bars), and *P* values as calculated by cox proportional hazard regression are displayed in the forest plot (middle). **e**, Kaplan Meier survival curves of ICR clusters for OS (left) and PFI (right). HRs and 95%-confidence intervals are calculated by cox proportional hazard regression. Overall *P* value is calculated by log-rank test. Vertical lines indicate censor points. **f**, Kaplan Meier survival curves of CMS for OS (left) and PFI (right). HRs are calculated by cox proportional hazard regression. Overall *P* value is calculated by log-rank test. Vertical lines indicate censor points. **g**, Circos plot of the interrelation between ICR and CMS classifications. Size of each element is proportional to number of samples in each respective category. **h**, PFI curve of ICR clusters within the CMS4 subtype. HRs and 95%-confidence intervals are calculated by cox proportional hazard regression. Overall *P* value is calculated by log-rank test. Vertical lines indicate censor points. Hazard Ratio (HR). Overall Survival (OS). Progression Free Interval (PFI). All *P* values are two-sided; *n* reflects the independent number of samples in all panels.

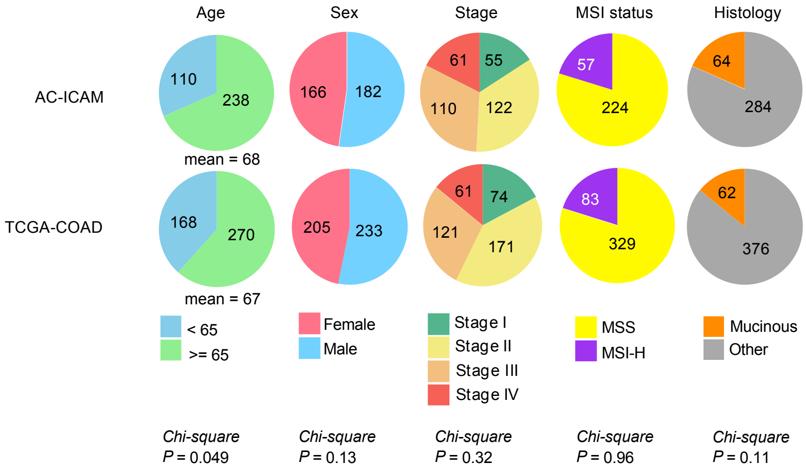

Supplementary Figure 3. Comparison of clinical data between AC-ICAM and TCGA-COAD. Schematic representation of distribution of clinico-pathological, and molecular features in AC-ICAM and TCGA-COAD. For AC-ICAM all characteristics are available for all patients (*n* = 348), except for MSI status which was only available for patients with available WES data (*n* = 281). For TCGA-COAD all characteristics are available for all patients (*n* = 438), except for stage (available for 427 patients) and MSI status (available for 412 samples). All *P* values are two-sided; *n* reflects the independent number of samples.

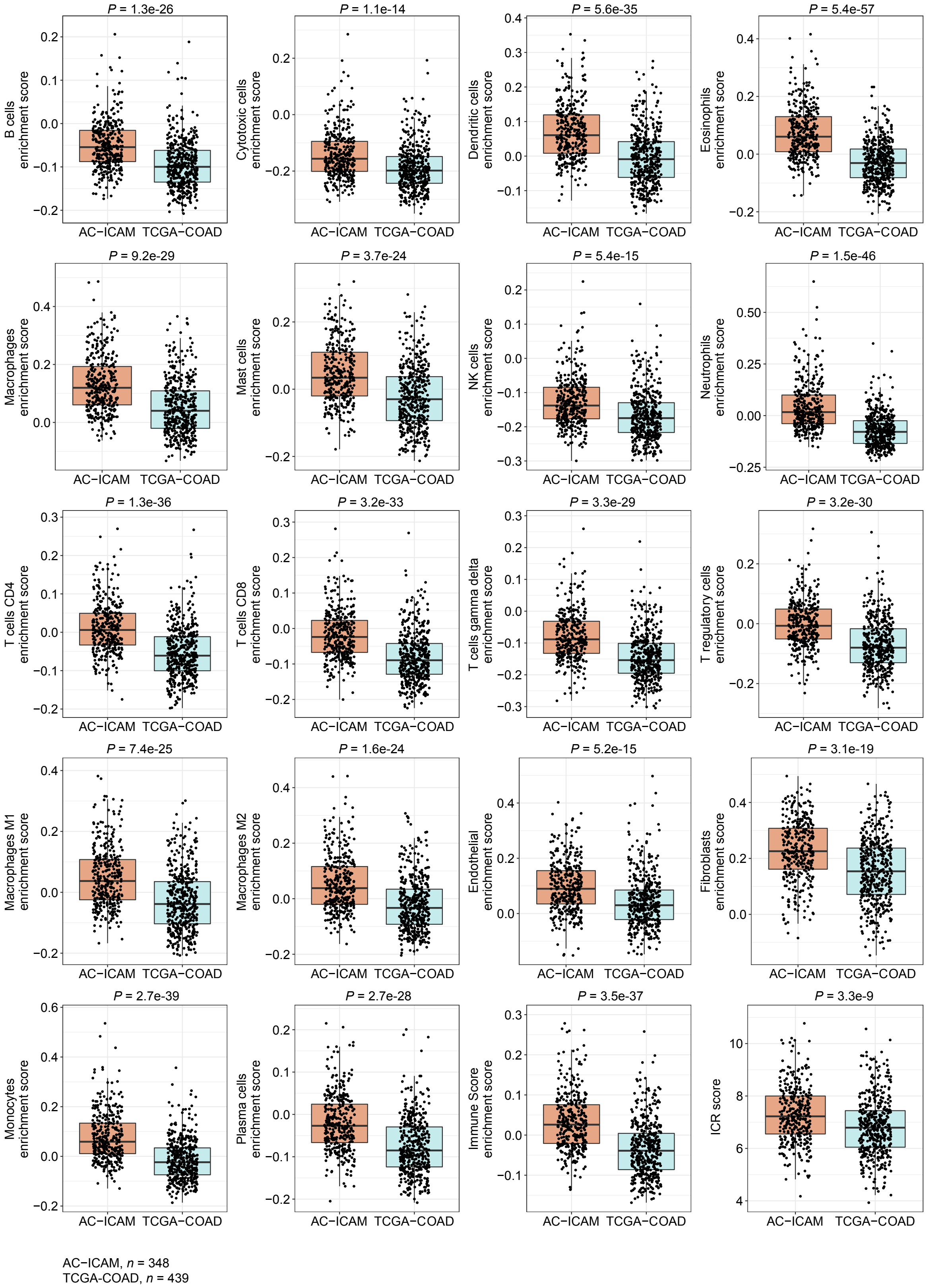

Supplementary Figure 4. Comparison AC-ICAM and TCGA-COAD ConsensusTME and ICR score. ConsensusTME and ICR scores in AC-ICAM and TCGA-COAD using re-normalized expression matrix. *P* values are calculated using an unpaired t test (two-sided). Center line, box limits, and whiskers represent the median, interquartile range and 1.5x interquartile range respectively. *n* = 348 independent samples from individual patients from AC-ICAM, and *n* = 439 independent samples from individual patients from TCGA-COAD.

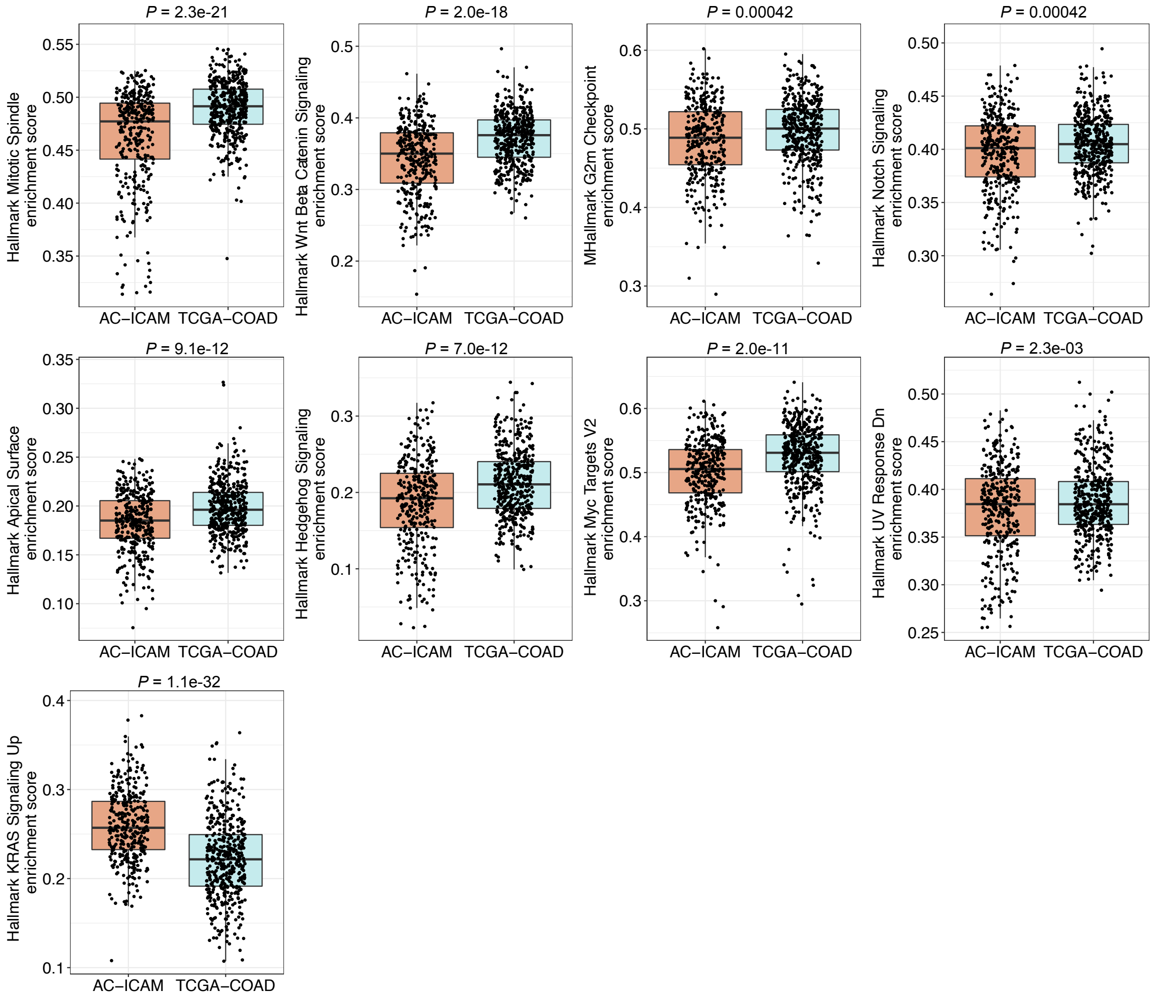

AC−ICAM, *n* = 348
TCGA-COAD, *n* = 439

Supplementary Figure 5. Comparison AC-ICAM with TCGA-COAD Hallmark signatures. Hallmark pathways that are significantly enriched in the TCGA-COAD compared to AC-ICAM using the renormalized expression matrix (unpaired t-test, $P < 0.05$, FDR < 0.1). *P* values are calculated using an unpaired t test (two-sided). FDR is calculated using Benjami-Hochberg's method. Center line, box limits, and whiskers represent the median, interquartile range and 1.5x interquartile range respectively. *n* = 348 independent samples from individual patients from AC-ICAM, and *n* = 439 independent samples from individual patients from TCGA-COAD.

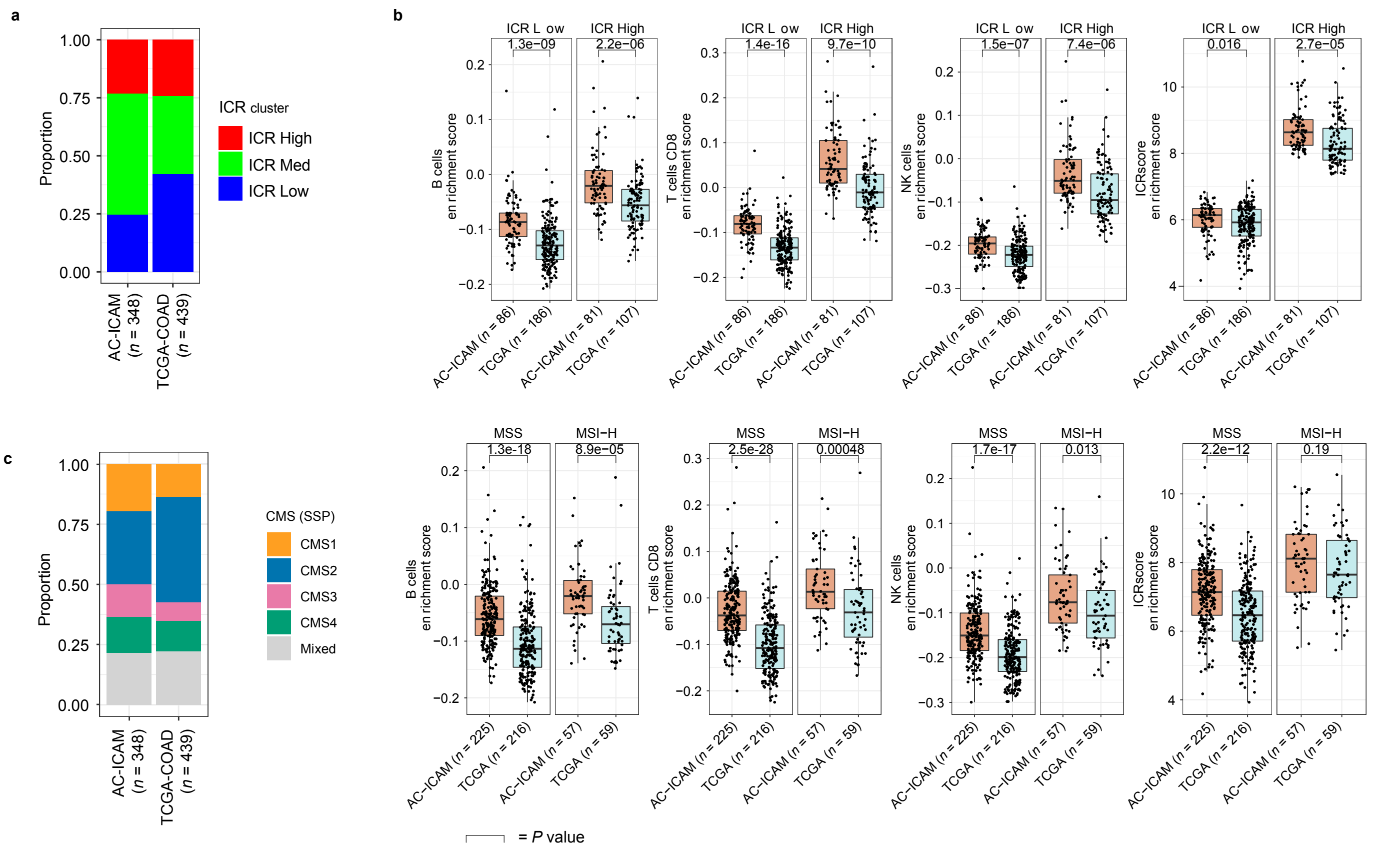

Supplementary Figure 6. Comparison ICR, immune subsets, and CMS distribution between TCGA-COAD and AC-ICAM. **a**, Stacked barcharts reflect the relative proportion of patients assigned to each ICR cluster in AC-ICAM and TCGA-COAD. **b**, B cell, CD8 T cell, and NK cell enrichment scores and ICR score calculated from combined, re-normalized matrix compared between AC-ICAM and TCGA-COAD samples within ICR clusters (upper panel) and within MSI status subgroups (lower panel). *P* values are calculated using unpaired, two-sided t-test. Center line, box limits, and whiskers represent the median, interquartile range and 1.5x interquartile range respectively. **c**, Stacked barcharts reflect the relative proportion of patients assigned to each CMS using the SSP algorithm. SSP: single sample predictor. All *P* values are two-sided; *n* reflects the independent number of samples in all panels.

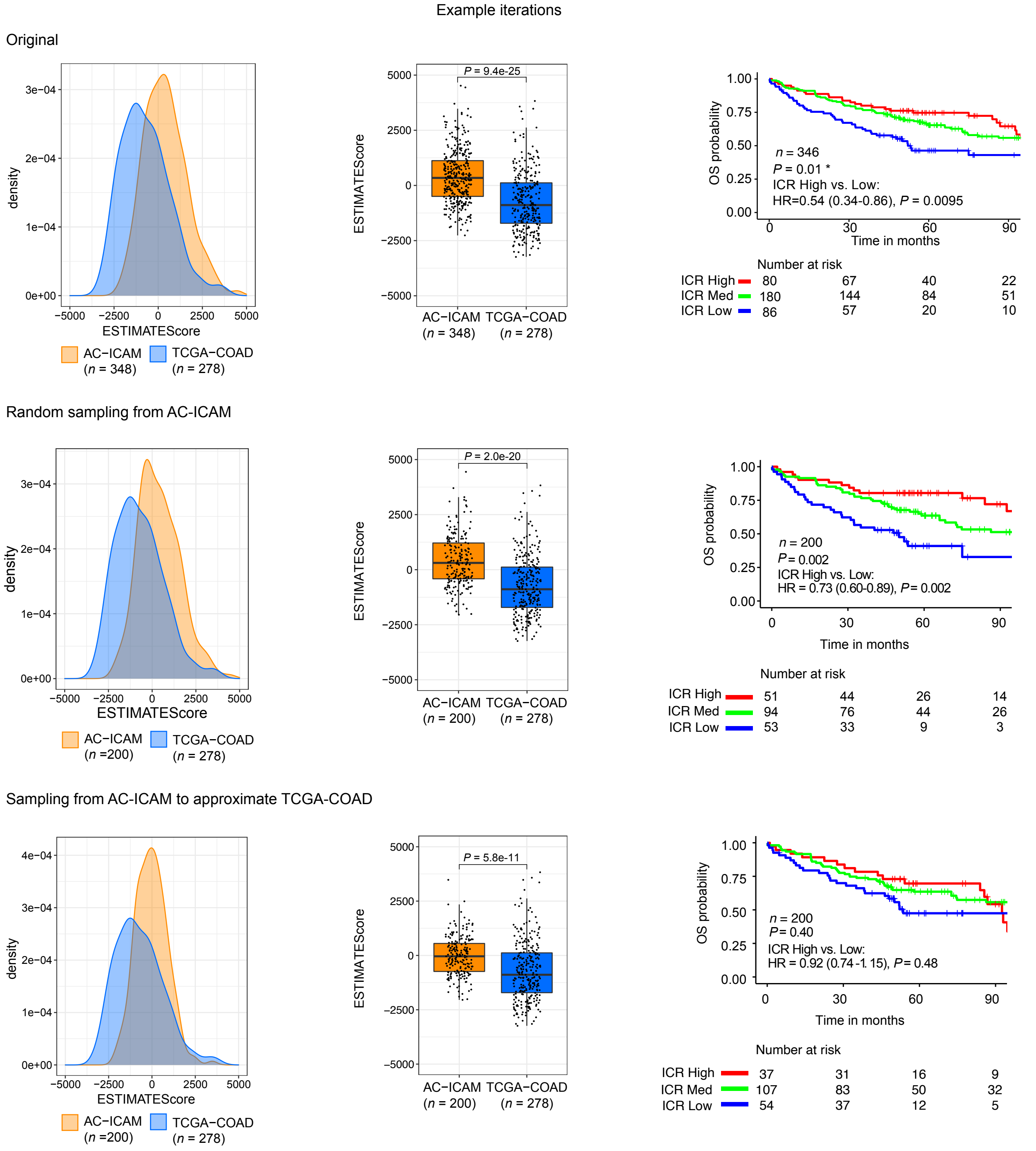

Supplementary Figure 7. Example iterations for subsampling for Cox Proportional Hazard analysis. Original distribution of AC-ICAM and TCGA-COAD ESTIMATEScore and illustrative examples of single iterations (R seed of 81) of random subsampling of AC-ICAM and sampling of AC-ICAM to approximate the TCGA-COAD distribution of ESTIMATE scores. The R “stats” function “approxfun()” was used to perform (linear or constant) interpolation of the ESTIMATE scores from TCGA. The resulting probability function approximating the ESTIMATE score distribution in TCGA was then used to sample from AC-ICAM data points. Density curves (left), boxplots (middle), and corresponding Kaplan Meier curves for OS by ICR cluster (right) are visualized for each example permutation. For the boxplots, center line, box limits, and whiskers represent the median, interquartile range and 1.5x interquartile range respectively, *P* values are calculated using unpaired t-test. For Kaplan-Meier curves: Overall *P* value is calculated by log-rank test and *P* value corresponding to HR is calculated using cox proportional hazard regression. *P* values are two-sided. Overall Survival (OS). *n* reflects the independent number of samples in each of the example iterations.

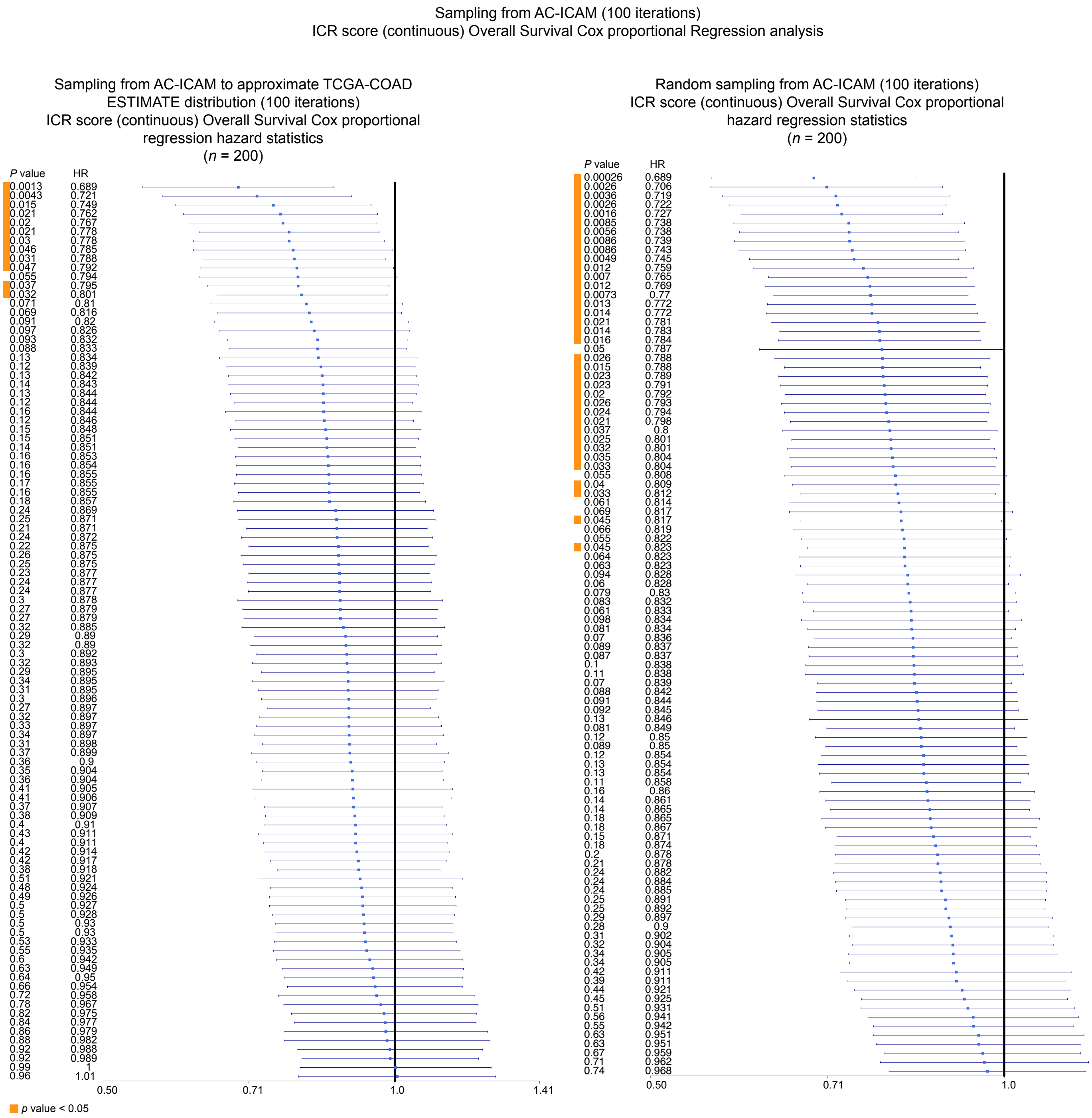

Supplementary Figure 8. Cox proportional hazard on subsampled data from AC-ICAM, 100 iterations. Forest plots of Cox Proportional Hazard Regression analysis for the association between ICR score as continuous variable and OS in subsamples of the AC-ICAM dataset (100 iterations). On the left side, subsampling to approximate TCGA-COAD ESTIMATE distribution, and on the right side, random subsampling. HRs, 95%-confidence intervals (error bars), and corresponding *P* values are calculated using cox proportional hazard regression analysis. Hazard ratio (HR). Overall Survival (OS). All *P* values are two-sided; *n* reflects the independent number of samples in all panels.

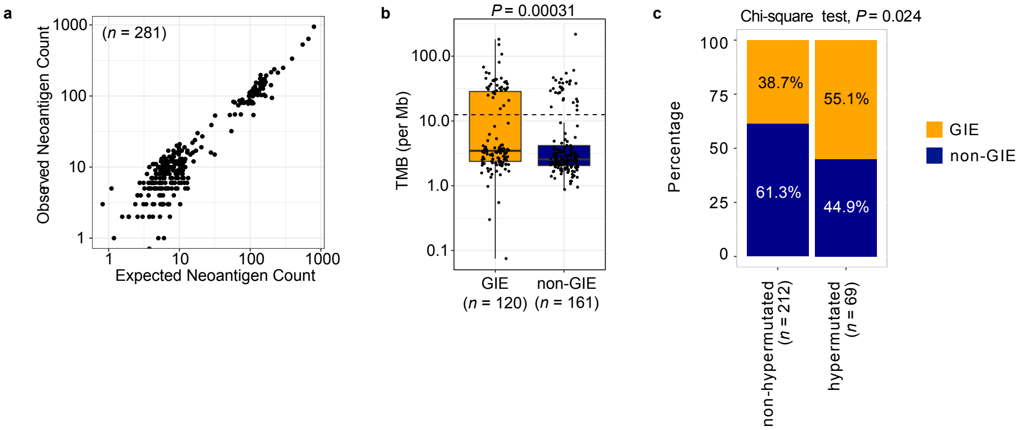

Supplementary Figure 9. Neoantigens, GIE and hypermutation status. a, Scatterplot of expected versus observed number of neoantigens for each sample. **b**, Tumor Mutational Burden (TMB) per Mb for GIE and non-GIE samples. Mann Whitney U-test statistic (two-sided) is indicated in the plot. Center line, box limits, and whiskers represent the median, interquartile range and 1.5x interquartile range respectively. **c**, Proportion of hypermutated and non-hypermutated samples with GIE or without GIE. GIE in non-hypermutated tumors is 38.7% vs 55.1% in hypermutated samples. Chi-square test significance is indicated in the plot. Genetic ImmunoEditing (GIE). All *P* values are two-sided; n reflects the independent number of samples in all panels.

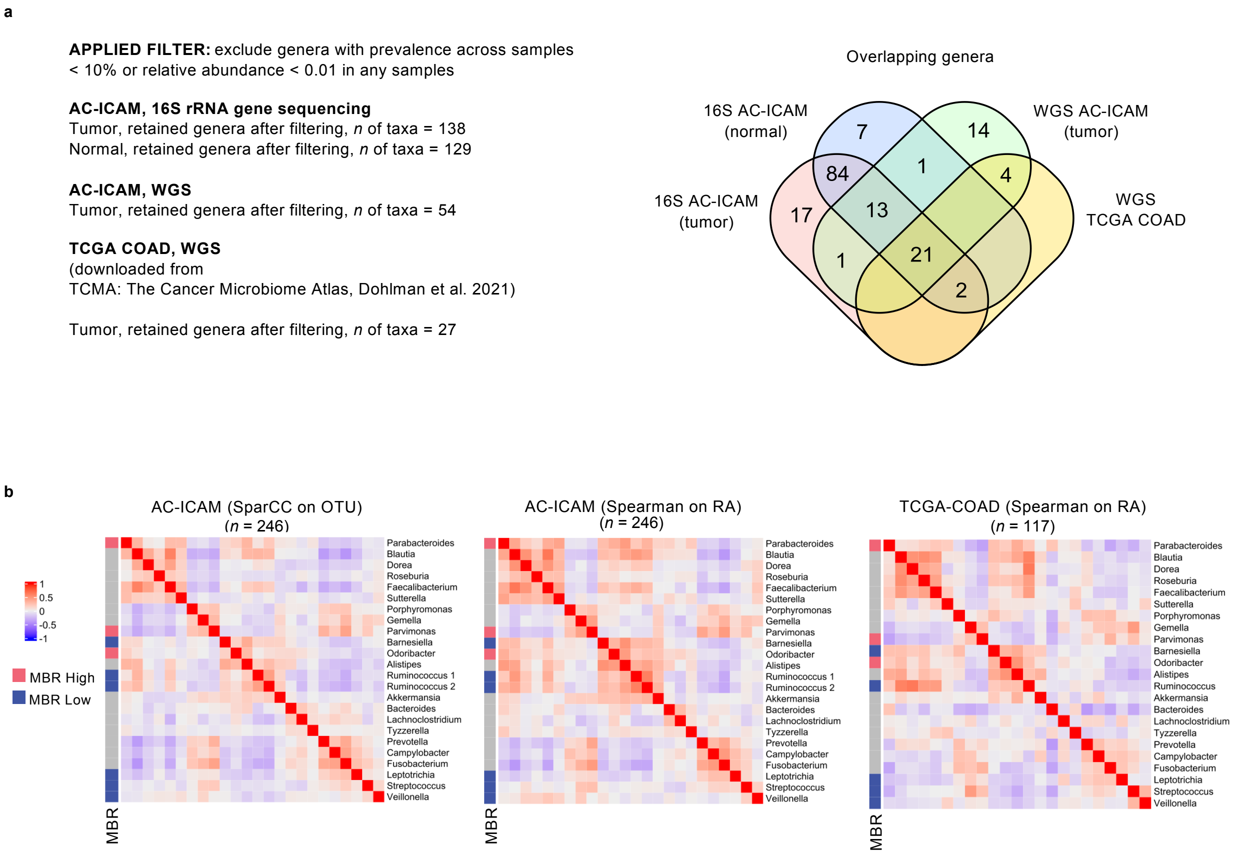

Supplementary Figure 10. TCGA-COAD and AC-ICAM microbiome overlap. a, Venn diagram showing overlap between the microbiome composition in TCGA-COAD and AC-ICAM. Genera from TCGA-COAD and AC-ICAM were filtered to only include those with a prevalence >10% samples and relative abundance of >0.01 in any sample. From AC-ICAM, 16S rRNA gene sequencing, 138 genera were retained for tumor samples, 129 genera for normal and 54 genera from AC-ICAM WGS tumor samples. For TCGA-COAD, only 27 genera were retained after applying the filter for tumor samples. For 16S rRNA gene sequencing in AC-ICAM, two *Ruminococcus* taxa (*Ruminococcus 1* and *Ruminococcus 2*) are identified, while for WGS metagenomic analysis in both AC-ICAM and TCGA-COAD only a single taxon of *Ruminococcus* is identified. Therefore, *Ruminococcus 2* is relabeled as *Ruminococcus* in 16S AC-ICAM genera matrix, while *Ruminococcus 1* label was kept and it overlaps only between 16S AC-ICAM tumor and normal samples. **b**, SparCC on OTU and Spearman correlation heatmap of the overlapping genera between AC-ICAM (*n* = 24 genera) and TCGA-COAD (*n* = 23 genera) that passed the minimum abundance filter in both cohorts (at least present in >10% of the tumor samples and a minimal relative abundance of 0.01 in at least one samples). In AC-ICAM, two *Ruminococcus* taxa (*Ruminococcus 1* and *Ruminococcus 2*) are represented, while in TCGA-COAD only a single taxon of *Ruminococcus* is included. Genera are ordered according to hierarchical clustering of the Spearman correlation matrix of AC-ICAM.

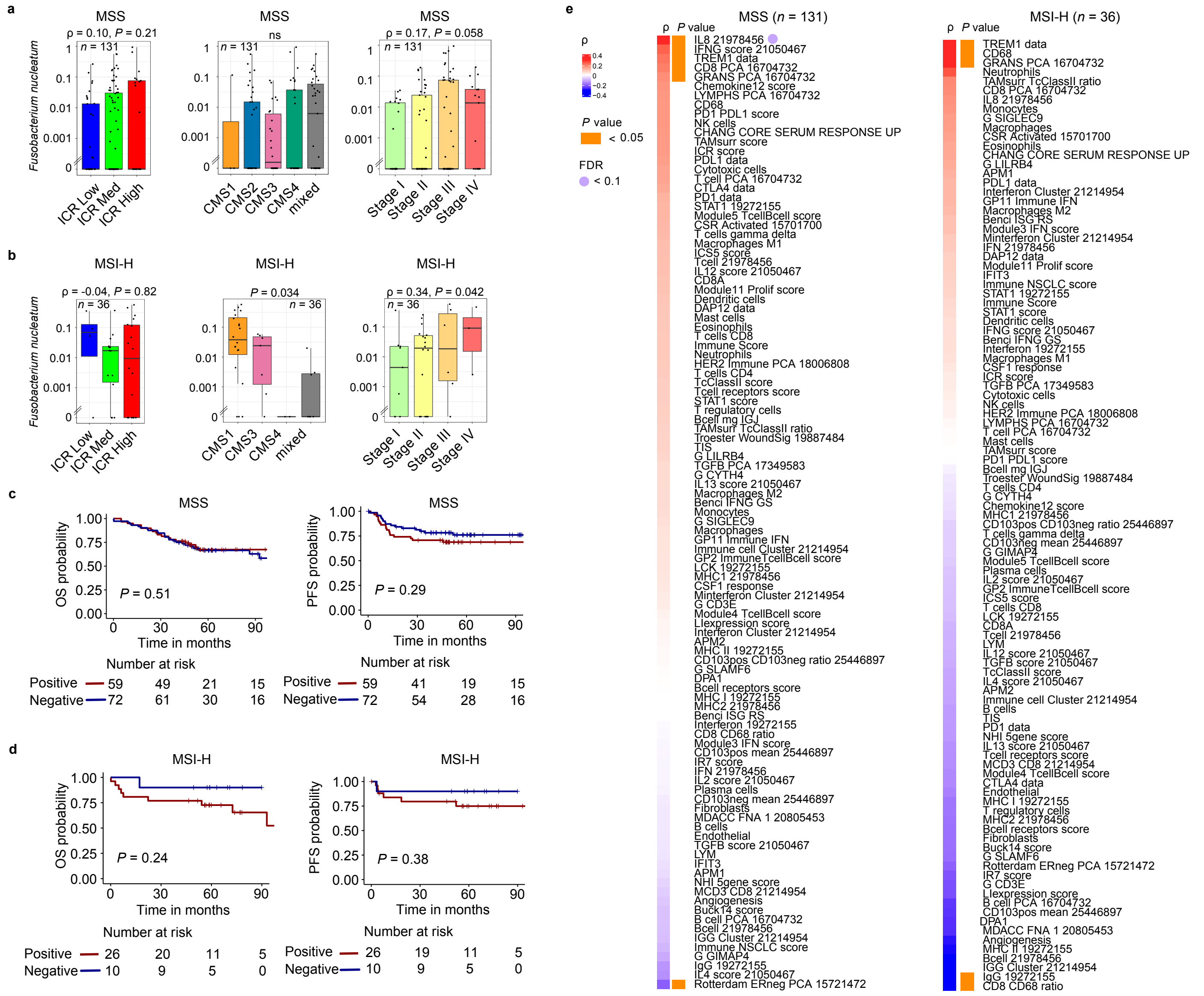

Supplementary Figure 11. Relation between relative abundance of *Fusobacterium nucleatum* and tumor characteristics in AC-ICAM stratified by MSI-H and MSS. a, Boxplot for relative abundance of *Fusobacterium nucleatum* as determined by metagenomic profiling of WGS data in MSS tumor samples (*n* = 131 tumor samples) by ICR cluster (left), CMS (middle), and by pathological stage (right), *P* values are calculated using unpaired t-test. Center line, box limits, and whiskers represent the median, interquartile range and 1.5x interquartile range respectively. **b**, Same as a, but in MSI-H tumor samples (*n* = 36). **c**, Kaplan-Meier curves corresponding to patients with tumor samples with a relative abundance of *Fusobacterium nucleatum* higher than median compared to those lower than median in MSS tumor samples (*n* = 131). Overall *P* value is calculated by log-rank test. Vertical lines indicate censor points. Overall Survival (OS). Progression Free Survival (PFS). **d**, Same as **c**, but in MSI-H (*n* = 36). **e**, Spearman correlation (two-sided) between the relative abundance of *Fusobacterium nucleatum* in tumor samples and immune gene signatures in MSS tumor samples (*n* = 131, left) and MSI-H (*n* = 36, right). All *P* values are two-sided; *n* reflects the independent number of samples in all panels.

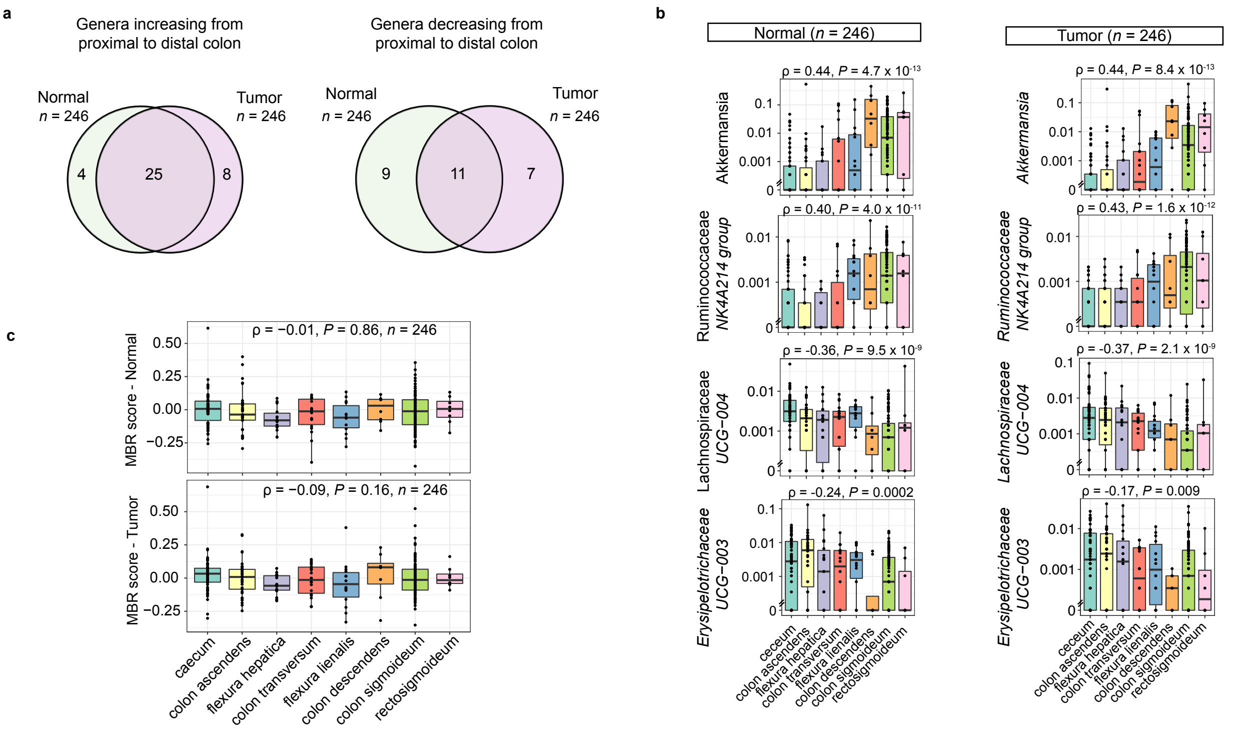

d

n = 246

Genus	ρ in Tumor	*P* value in Tumor	FDR in Tumor	ρ in Normal	*P* value in Norma	FDR in Normal
Veillonella	-0.20	1.3E-03	7.8E-03	-0.32	2.2E-07	4.1E-06
Treponema 2	-0.17	6.2E-03	2.6E-02	Filtered out	Filtered out	Filtered out
Erysipelotrichaceae UCG-003	-0.17	8.7E-03	3.5E-02	-0.24	1.9E-04	1.5E-03
Erysipelatoclostridium	-0.16	1.1E-02	4.0E-02	-0.22	6.1E-04	3.0E-03
Lachnospiraceae NK4A136 group	-0.16	1.3E-02	4.5E-02	-0.14	2.4E-02	6.5E-02
Oscillibacter	0.14	2.6E-02	7.7E-02	0.22	4.5E-04	2.3E-03
UBA1819	0.16	1.1E-02	4.0E-02	0.23	3.3E-04	1.9E-03
Bifidobacterium	0.18	5.7E-03	2.5E-02	0.16	1.4E-02	4.3E-02
Ruminiclostridium 6	0.19	3.3E-03	1.6E-02	0.19	3.2E-03	1.3E-02
Barnesiella	0.22	6.1E-04	4.0E-03	0.16	9.8E-03	3.2E-02
Ruminococcus 1	0.24	1.3E-04	1.1E-03	0.27	2.0E-05	2.1E-04
Family XIII AD3011 group	0.25	8.4E-05	7.8E-04	0.29	3.2E-06	4.1E-05
Ruminococcus 2	0.36	9.9E-09	2.7E-07	0.29	5.3E-06	6.2E-05
Ruminococcaceae NK4A214 group	0.43	1.6E-12	1.1E-10	0.40	4.0E-11	2.6E-09

Supplementary Figure 12. Microbiome by anatomical location. **a**, Venn diagram of genera showing the overlap between genera that have a significant change in abundance from the proximal to distal colon calculated by Spearman correlation in the normal colon tissue versus tumor tissue ($P < 0.05$, FDR < 0.1). **b**, Most significantly altered genera changing in abundance from proximal to distal colon. *Akkermansia* was the most significant, both in normal and tumor tissues. *Ruminococcus NK4A214 group* was the second most significant, both in normal and tumor tissues. *Lachnospiraceae* was the genus with the strongest inverse Spearman correlation with anatomical location, both in the normal as well as in tumor tissue. *Erysipeltrichaceae UCG-003* was included as this genus is part of the MBR classifier. All correlations of the represented genera pass FDR < 0.1. Center line, box limits, and whiskers represent the median, interquartile range and 1.5x interquartile range respectively. **c**, MBR score calculated in normal tissue (left) and tumor tissue (right) by anatomical location. Spearman correlation coefficient (Rho) and *P* value are indicated in the plot. Center line, box limits, and whiskers represent the median, interquartile range and 1.5x interquartile range respectively. **d**, Spearman correlation statistics of all genera in the tumor from the MBR classifier with a significant (FDR<0.1) association with anatomical location (ordinal variable). All *P* values are two-sided; *n* reflects the independent number of samples in all panels.

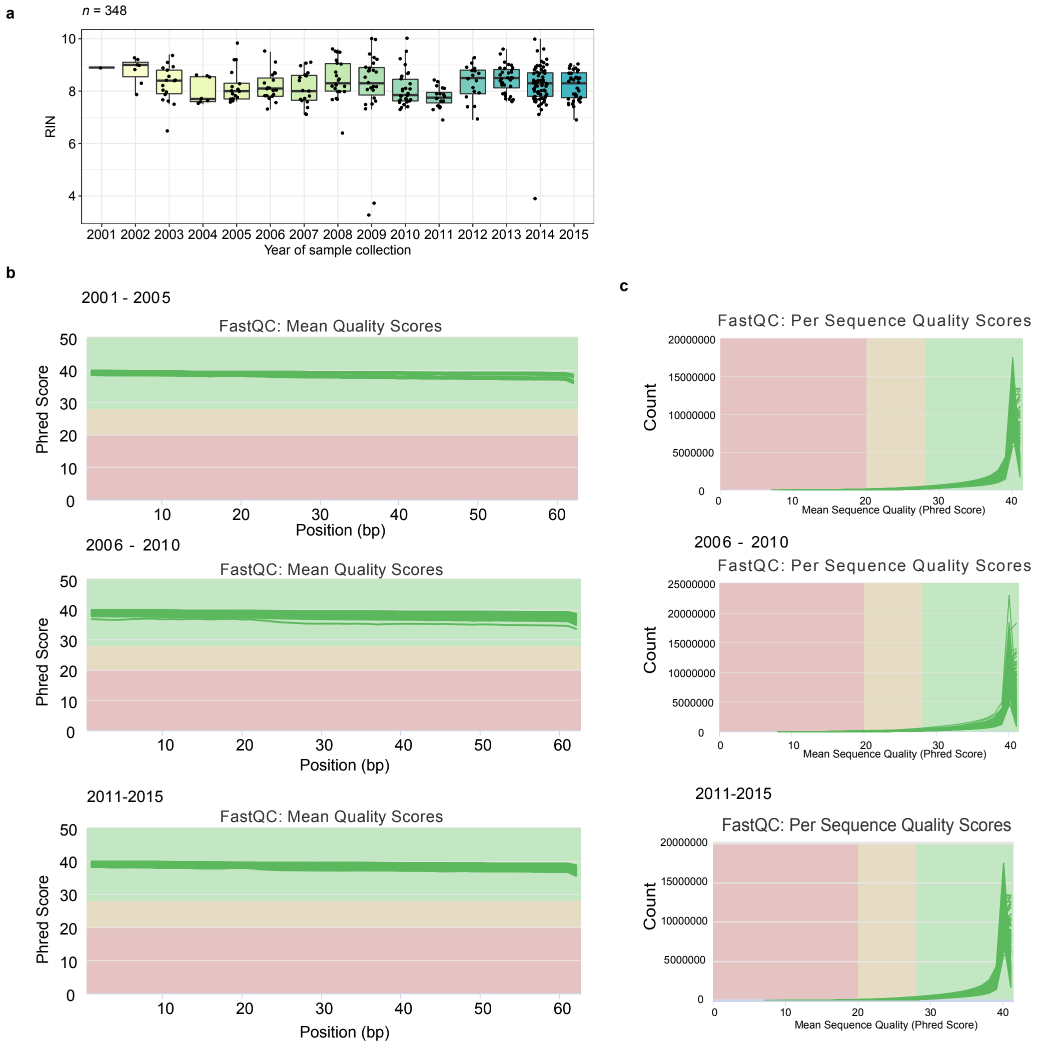

Supplementary Figure 13. Quality control of samples and generated data by year of sample collection for RNASeq. a, RNA Integrity Number (RIN) of extracted RNA from resected, frozen colon cancer samples collected over the years. Center line, box limits, and whiskers represent the median, interquartile range and 1.5x interquartile range respectively. **b**, Mean quality scores from FastQC for RNASeq data run on HiSeq4000. Plot reflects the mean quality value across each base position in the read. **c**, Per sequence quality scores from FastQC for RNASeq data. The number of reads with average quality scores is plotted. *n* reflects the independent number of samples in all panels.

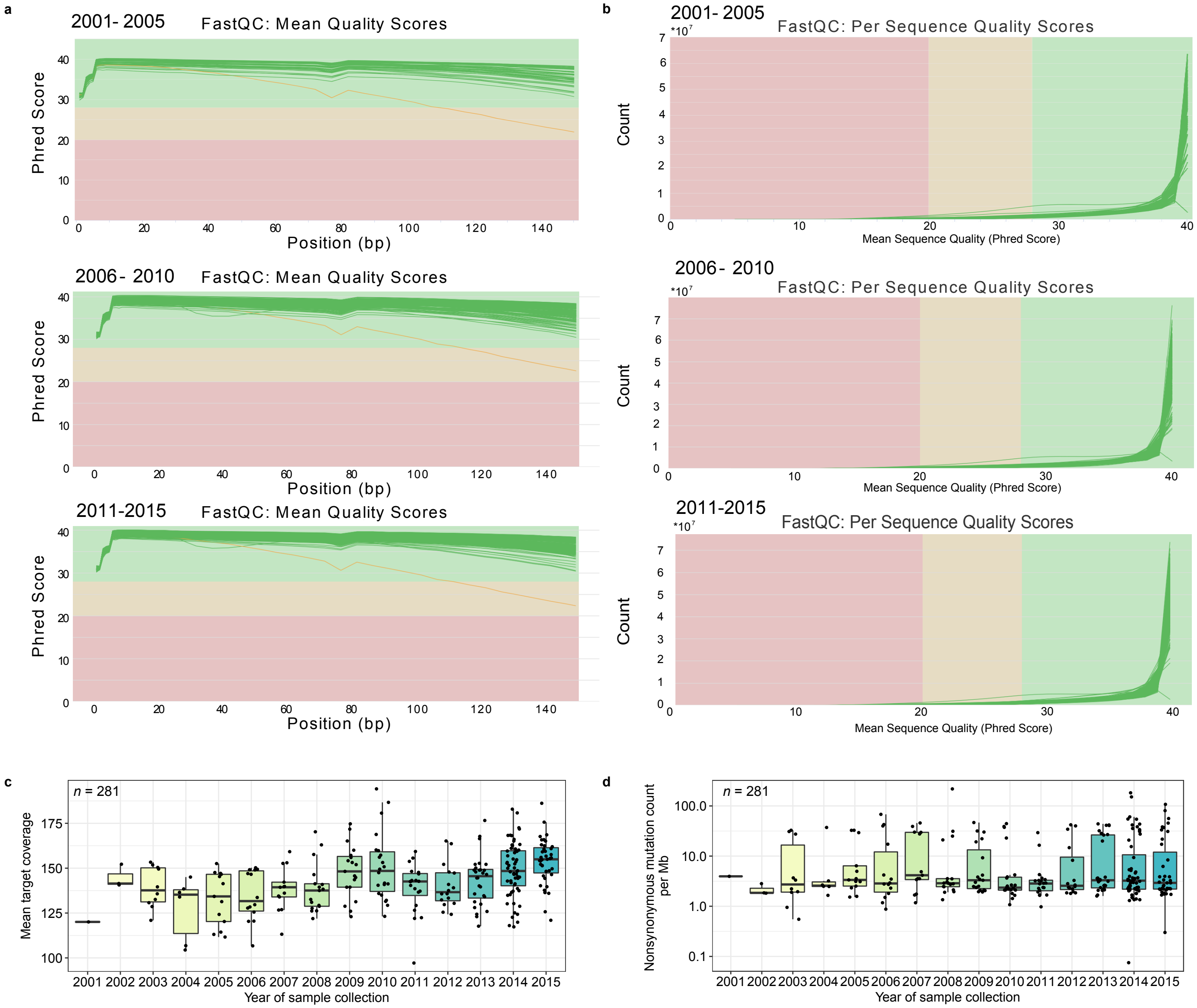

Supplementary Figure 14. Quality control of samples and generated data by year of sample collection for WES. a, Mean quality scores from FastQC for WES data. Plot reflects the mean quality value across each base position in the read. **b**, Per sequence quality scores from FastQC for WES data. The number of reads with average quality scores is plotted. **c**, Mean target coverage (WES) by year of sample collection. Center line, box limits, and whiskers represent the median, interquartile range and 1.5x interquartile range respectively. **d**, Mean target coverage an nonsynonymous mutation count per Mb by year of sample collection. Center line, box limits, and whiskers represent the median, interquartile range and 1.5x interquartile range respectively. *n* reflects the independent number of samples in all panels.

Supplementary Table 1. Clinical data overview AC-ICAM (*n* = 348)

**Sex**	*n*	%
Male	166	47.7
Female	182	52.3
**Age**		
<50 years	23	6.6
50-65 years	87	25.0
65-75 years	123	35.3
>= 75 years	115	33.0
**T stage**		
T1	16	4.6
T2	53	15.2
T3	243	69.8
T4	36	10.3
**N stage**		
N0	190	54.6
N1	90	25.9
N2	68	19.5
**M stage**		
M0	286	82.2
M1	39	11.2
Not available	23	6.6
**TNM stage**		
I	55	15.8
II	122	35.1
III	110	31.9
IV	61	17.2
**Year of diagnosis**		
2001	1	0.3
2002	7	2.0
2003	17	4.9
2004	7	2.0
2005	17	4.9
2006	19	5.5
2007	19	5.5
2008	23	6.6
2009	27	7.8
2010	30	8.6
2011	20	5.7
2012	17	4.9
2013	32	9.2
2014	73	21.0
2015	39	11.2

**Tumor anatomic location**		*n*	%
**Right sided**		183	52.6
	ceceum	79	22.7
	colon ascendens	52	14.9
	flexura hepatica	27	7.8
	colon transversum	25	7.2
**Left sided**		165	47.4
	flexura lienalis	20	5.7
	colon descendens	14	4.0
	colon sigmoideum	121	34.8
	rectosigmoideum	10	2.9
**Tumor morphology**			
	adenocarcinoma	198	56.9
	adenocarcinoma intestinal type	76	21.8
	mucineus adenocarcinoma	64	18.4
	signet ring cell carcinoma	3	0.9
	adenocarcinoma in villeus adenoom	2	0.6
	adenocarcinoma with mixed subtypes	3	0.9
	cribriform carcinoma	2	0.6
**Adjuvant treatment**			
No treatment		238	68.4
Adjuvant treated		110	31.6
	systemic chemotherapy	47	13.5
	chemotherapy incl. platinum	56	16.1
	chemotherapy with levamisol/leucovorin/ledervorin	1	0.3
	targeted chemo mAb	8	2.3
	radiotherapy	1	0.3
**Recurrences**			
	local	17	4.9
	regional	18	5.2
	distant	88	25.3
**History of cancer**			
	Yes	85	24.4
	No	260	74.7
**Second primary tumor in follow up**			
	Yes	69	19.8
	No	279	80.2

Supplementary Table 12 - The STORMS checklist. An editable version for adaptation and inclusion in publications is available from https://stormsmicrobiome.org

Number	Item	Recommendation	Item Source	Additional Guidance	Yes/No/NA	Comments or location in manuscript
**Abstract**						
1.0	Structured or Unstructured Abstract	Abstract should include information on background, methods, results, and conclusions in structured or unstructured format.	STORMS		Yes	Page 2
1.1	Study Design	State study design in abstract.	STORMS	See 3.0 for additional information on study design.	Yes	Page 2 (Cohort Study)
1.2	Sequencing methods	State the strategy used for metagenomic classification.	STORMS	For example, targeted 16S RNA gene expression by qPCR or sequencing, shotgun metagenomics, metatranscriptomics, etc.	Yes	Page 2
1.3	Specimens	Describe body site(s) studied.	STORMS		Yes	Page 2
**Introduction**						
2.0	Background and Rationale	Summarize the underlying background, scientific evidence, or theory driving the current hypothesis as well as the study objectives.	STORMS		Yes	Pages 2-3
2.1	Hypotheses	State the pre-specified hypothesis. If the study is exploratory, state any pre-specified study objectives.	STORMS		Yes	Pages 3-4
**Methods**						

3.0	Study Design	Describe the study design.	STORMS	Observational (Case-Control, Cohort, Cross-sectional survey, etc.) or Experimental (Randomized controlled trial, Non-randomized controlled trial, etc.). For a brief description of common study designs see: DOI: 10.11613/BM.2014.022<br><br>If applicable, describe any blinding (e.g. single or double-blinding) used in the course of the study.	Yes	Page 28 (Cohort Study)
3.1	Participants	State what the population of interest is, and the method by which participants are sampled from that population. Include relevant information on physiological state of the subjects or stage in the life history of disease under study when participants were sampled.	STORMS	Examples of the population of interest could be: adults with no chronic health conditions, adults with type II diabetes, newborns, etc. This is the total population to whom the study is hoped to be generalizable to. The sampling method describes how potential participants were selected from that population.<br><br>If the participants are from a substudy of a larger study, provide a brief description of that study and cite that study.<br><br>Clearly state how cases and controls are defined.<br><br>An example of relevant physiological state might be pre/post-menopausal for a vaginal microbiome study; examples of stage in the life history of disease could be whether	Yes	Page 28, and Supplementary_Data.sheet 2.1 (variables) and 2.2 (codebook).

				specimens were collected during active or dormant disease, or before or after treatment.		
3.2	Geographic location	State the geographic region(s) where participants were sampled from.	MIxS: geographic location (country and/or sea,region)	Geographic coordinates can be reported to prevent potential ambiguities if necessary.	Yes	Page 28 (The Netherlands)
3.3	Relevant Dates	State the start and end dates for recruitment, follow-up, and data collection.	STORMS	Recruitment is the period in which participants are recruited for the study. In longitudinal studies, follow-up is the date range in which participants are asked to complete a specific assessment. Finally, data collection is the total period in which data is being collected from participants including during initial recruitment through all follow-ups.	Yes	Page 28

3.4	Eligibility criteria	List any criteria for inclusion and exclusion of recruited participants.	Modified STROBE	Among potential recruited participants, how were some chosen and others not? This could include criteria such as sex, diet, age, health status, or BMI.<br><br>If there is a primary and validation sample, describe inclusion/exclusion criteria for each.	Yes	Page 28 and Extended Data Fig. 1
3.5	Antibiotics Usage	List what is known about antibiotics usage before or during sample collection.	STORMS	If participants were excluded due to current or recent antibiotics usage, state this here.<br><br>Other factors (e.g. proton pump inhibitors, probiotics, etc.) that may influence the microbiome should also be described as well.	NA	Information on antibiotic usage and other factors that may influence the microbiome was not collected
3.6	Analytic sample size	Explain how the final analytic sample size was calculated, including the number of cases and controls if relevant, and reasons for dropout at each stage of the study. This should include the number of individuals in whom microbiome sequencing was attempted and the number in whom microbiome sequencing was successful.	STORMS	Consider use of a flow diagram (see template at https://stormsmicrobiome.org/figures). Also state sample size in abstract.<br><br>If power analysis was used to calculate sample size, describe those calculations.	Yes	Page 28, 30, 46 and 54, and Extended Data Fig. 1.
3.7	Longitudinal Studies	For longitudinal studies, state how many follow-ups were conducted, describe sample size at follow-up by group or condition, and discuss any loss to follow-up.	STORMS	If there is loss to follow-up, discuss the likelihood that drop-out is associated with exposures, treatments, or outcomes of interest.	Yes	Survival curves indicating patients at risk, Fig. 1, 4, 5, and 6

3.8	Matching	For matched studies, give matching criteria.	Modified STROBE	"Matched" refers to matching between comparable study participants as cases and controls or exposed / unexposed.<br><br>Indicate whether participants were individual or frequency matched and in what ratio were they matched (e.g. 1 case to 1 control).	NA	Page 28 There was no matching for recruited subjects; instead, we collected matched tumor and normal colon tissue
3.9	Ethics	State the name of the institutional review board that approved the study and protocols, protocol number and date of approval, and procedures for obtaining informed consent from participants.	STORMS		Yes	Page 28
4.0	Laboratory methods	State the laboratory/center where laboratory work was done.	STORMS	Provide a reference to complete lab protocols if previously published elsewhere such as on protocols.io. Note any modifications of lab protocols and the reason for protocol modifications.	Yes	Page 44-48
4.1	Specimen collection	State the body site(s) sampled from and how specimens were collected.	MIxS: sample collection device or method; host body site	Use terms from the Uber-anatomy Ontology (https://www.ebi.ac.uk/ols/ontologies/uberon) to describe body sites in a standardized format.	Yes	Page 28
4.2	Shipping	Describe how samples were stored and shipped to the laboratory.	STORMS	Include length of time from collection to receipt by the lab and if temperature control was used during shipping.	Yes	Page 30

4.3	Storage	Describe how the laboratory stored samples, including time between collection and storage and any preservation buffers or refrigeration used.	STORMS	State where each procedure or lot of samples was done if not all in the same place.<br><br>Include reagent/lot/catalogue #s for storage buffers.	Yes	Page 30
4.4	DNA extraction	Provide DNA extraction method, including kit and version if relevant.	MIxS: nucleic acid extraction	If any DNA quantification methods were used prior to DNA amplification or at the pooling step of library preparation, state so here.	Yes	Page 30
4.5	Human DNA sequence depletion or microbial DNA enrichment	Describe whether human DNA sequence depletion or enrichment of microbial or viral DNA was performed.	STORMS		Yes	Page 45
4.6	Primer selection	Provide primer selection and DNA amplification methods as well as variable region sequenced (if applicable).	MIxS: pcr primers		Yes	Page 45 and 48
4.7	Positive Controls	Describe any positive controls (mock communities) if used.	STORMS	If used, should be deposited under guidance provided in the 8.X items.	Yes	Page 45
4.8	Negative Controls	Describe any negative controls if used.	STORMS	If used, should be deposited under guidance provided in the 8.X items.	Yes	Page 46
4.9	Contaminant mitigation and identification	Provide any laboratory or computational methods used to control for or identify microbiome contamination from the environment, reagents, or laboratory.	STORMS	Includes filtering of reagents and other steps to minimize contamination. It is relevant to state whether the specimens of interest have low microbial load, which makes contamination especially relevant.	Yes	Page 49-50

4.10	Replication	Describe any biological or technical replicates included in the sequencing, including which steps were replicated between them.	STORMS	Replication may be biological (redundant biological specimens) or technical (aliquots taken at different stages of analysis) and used in extraction, sequencing, preprocessing, and/or data analysis.	Yes	We validated 16S findings with WGS and PCR. Pages 46-48
4.11	Sequencing strategy	Major divisions of strategy, such as shotgun or amplicon sequencing.	MIxS: sequencing method	For amplicon sequencing (for example, 16S variable region), state the region selected. State the model of sequencer used.	Yes	Pages 44-48
4.12	Sequencing methods	State whether experimental quantification was used (QMP/cell count based, spike-in based) or whether relative abundance methods were applied.	STORMS	These include read length, sequencing depth per sample (average and minimum), whether reads are paired, and other parameters.	Yes	Pages 46-48
4.13	Batch effects	Detail any blocking or randomization used in study design to avoid confounding of batches with exposures or outcomes. Discuss any likely sources of batch effects, if known.	STORMS	Sources of batch effects include sample collection, storage, library preparation, and sequencing and are commonly unavoidable in all but the smallest of studies.	Yes	Page 46
4.14	Metatranscriptomics	Detail whether any mRNA enrichment was performed and whether/how retrotranscription was performed prior to sequencing. Provide size range of isolated transcripts. Describe whether the sequencing library was stranded or not. Provide details on sequencing methods and platforms.	STORMS	Provide details on any internal standards which may have been used as well as parameters and versions of any software or databases used.	NA	No Metatranscriptomics analysis was performed
4.15	Metaproteomics	Detail which protease was used for digestion. Provide details on proteomic methods and platforms (e.g. LC-MS/MS, instrument type, column type, mass range, resolution, scan speed, maximum injection time, isolation window, normalised collision energy, and resolution).	STORMS	Provide details on any internal standards which may have been used as well as parameters and versions of any software or databases used.	NA	No Metaproteomics analysis was performed

4.16	Metabolomics	Specify the analytic method used (such as nuclear magnetic resonance spectroscopy or mass spectrometry). For mass spectrometry, detail which fractions were obtained (polar and/or non-polar) and how these were analyzed. Provide details on metabolomics methods and platforms (e.g. derivatization, instrument type, injection type, column type and instrument settings).	STORMS	Provide details on any internal standards which may have been used as well as parameters and versions of any software or databases used.	NA	No Metabolomics analysis was performed
5.0	Data sources/ measurement	For each non-microbiome variable, including the health condition, intervention, or other variable of interest, state how it was defined, how it was measured or collected, and any transformations applied to the variable prior to analysis.	MIxS: host disease status	State any sources of potential bias in measurements, for example multiple interviewers or measurement instruments, and whether these potential biases were assessed or accounted for in study design.<br><br>Use terms from a standardized ontology such as the Experimental Factor Ontology (https://www.ebi.ac.uk/efo/) to describe variables of interest in a standardized format.	Yes	Supplementary_Data.sheet 2.1 (variables) and 2.2 (codebook).
6.0	Research design for causal inference	Discuss any potential for confounding by variables that may influence both the outcome and exposure of interest. State any variables controlled for and the rationale for controlling for them.	STORMS	For causal inference, this item refers to describing the assumptions that would be required to draw causal inferences from observational data. See Vujkovic-Cvijin, I., Sklar, J., Jiang, L. et al. Host variables confound gut microbiota studies of human disease. Nature 587, 448–454 (2020). https://doi.org/10.1038/s41586-020-2881-9 for more details on confounding in observational microbiome studies.	Yes	Multivariate analyses, Pages 53-54

				For example, hypothesized confounders may be controlled for by multivariable adjustment. Consider using a directed acyclic graph (DAG) to describe your causal model and justify any variables controlled for. DAGs can be made using www.dagitty.net.		
6.1	Selection bias	Discuss potential for selection or survival bias.	STORMS	Selection bias can occur when some members of the target study population are more likely to be included in the study/final analytic sample than others. Some examples include survival bias (where part of the target study population is more likely to die before they can be studied), convenience sampling (where members of the target study population are not selected at random), and loss to follow-up (when probability of dropping out is related to one of the things being studied).	Yes	Page 28, Extended Data Fig. 1
7.0	Bioinformatic and Statistical Methods	Describe any transformations to quantitative variables used in analyses (e.g. use of percentages instead of counts, normalization, rarefaction, categorization).	STORMS	If a variable is analyzed using different transformations, state rationale for the transformation and for each analyses which version of the variable is used.<br><br>In case of any complex or multistep transformations, egive enumerated	Yes	Page 46

				instructions for reproducing those transformations.		
7.1	Quality Control	Describe any methods to identify or filter low quality reads or samples.	MIxS: sequence quality check	If samples were excluded based on quality or read depth, list the criteria used, the number of samples excluded, and the final sample size after quality control.	Yes	Page 45
7.2	Sequence analysis	Describe any taxonomic, functional profiling, or other sequence analysis performed.	MIxS: feature prediction; similarity search method		Yes	Pages 46-47
7.3	Statistical methods	Describe all statistical methods.	Modified STROBE	Describe any statistical tests used, exploratory data analysis performed, dimension reduction methods/unsupervised analysis, alpha/beta metrics, and/or methods for adjusting for measurement bias.<br><br>If multiple statistical methods are possible, discuss why the methods used were selected.<br><br>If a multiple hypothesis testing correction method was used, describe the type of correction used.<br><br>State which taxonomic levels are analyzed.	Yes	Pages 49-54

7.4	Longitudinal analysis	If the study is longitudinal, include a section that explicitly states what analysis methods were used (if any) to account for grouping of measurements by individual or patterns over time.	STORMS		NA	Samples were only collected at baseline
7.5	Subgroup analysis	Describe any methods used to examine subgroups and interactions.	STROBE		Yes	Page 50
7.6	Missing data	Explain how missing data were addressed.	STROBE	"Missing data" refers to participant measurements such as covariates, exposures, outcomes, or time points that should have been collected but were not, not to zeros in taxonomic abundance tables or data points not applicable to that observation.	Yes	Page 29
7.7	Sensitivity analyses	Describe any sensitivity analyses.	STROBE		Yes	Pages 51-54
7.8	Findings	State criteria used to select findings for reporting.	STORMS	For example, false discovery rate with total number of tests, effect size threshold, significance threshold, microbes of interest.	Yes	Pages 49-50
7.9	Software	Cite all software (including read mapping software) and databases (including any used for taxonomic reference or annotating amplicons, if applicable) used. Include version numbers.	Modified STREGA	Installed packages, add-ons or libraries should be stated and cited in addition to the software used.<br><br>All parameters employed that differ from the default of that software/version should be provided.<br><br>This is in addition to, not a replacement for, publishing of code as outlined in the section Reproducible Research.	Yes	Pages 45-54

8.0	Reproducible research	Make a statement about whether and how others can reproduce the reported analysis.	STORMS	Any protected information that has been excluded or provided under controlled access should be listed along with any relevant data access procedures. "On request from authors" is not sufficiently detailed; formal data access procedures and conditions should be defined.<br><br>If data are unavailable, state so clearly.<br><br>Consider using a specialized rubric for reproducible research (such as: https://mbio.asm.org/content/9/3/e00525-18.short).<br><br>Consider preregistering the study protocol (such as on osf.io or https://plos.org/open-science/preregistration/).	Yes	All raw and derived data is shared along with the code to analyze the data – See Data Availability Statement and Code Availability Statement, Pages 56-57
8.1	Raw data access	State where raw data may be accessed including demultiplexing information.	STORMS	Robust, long-term databases such as those hosted by NCBI and EBI are preferred. If using a private repository, provide rationale.	Yes	See Data Availability Statement and Code Availability Statement, Pages 56-57

8.2	Processed data access	State where processed data may be accessed.	STORMS	Unfiltered data should be provided.<br><br>Robust, long-term databases such as those hosted by NCBI and EBI-EMBL are preferred. Repositories like zenodo (https://zenodo.org/) or publisso (https://www.publisso.de/en/working-for-you/doi-service/) can be used to provide a DOI and long-term storage for processed datasets, even those which cannot be published openly.	Yes	See Data Availability Statement and Code Availability Statement, Pages 56-57
8.3	Participant data access	State where individual participant data such as demographics and other covariates may be accessed, and how they can be matched to the microbiome data.	STORMS	If re-categorized, transformed, or otherwise derived variables were used in the analysis, these variables or code for deriving them should be provided.<br><br>Examples of how participant data can be matched to microbiome data are: using the same set of anonymized identifiers, or using different anonymized identifiers but providing a map.<br><br>Provided data should be sufficient to independently replicate the current analysis.	Yes	See Data Availability Statement and Code Availability Statement, Pages 56-57
8.4	Source code access	State where code may be accessed.	STORMS	If a standard or formalized workflow was employed, reference it here.	Yes	See Code Availability Statement, Pages 57

8.5	Full results	Provide full results of all analyses, in computer-readable format, in supplementary materials.	STORMS	For example, any fold-changes, p-values, or FDR values calculated, provided as a spreadsheet.<br><br>Use a machine-readable, plain-text format such as csv or tsv.		Supplementary Tables 5,6,7,10, and 11
**Results**						
9.0	Descriptive data	Give characteristics of study participants (e.g. dietary, demographic, clinical, social) and information on exposures and potential confounders.	STROBE	Typically reported in a table included in the paper or as a supplementary table. Indicate number of participants with missing data for each variable of interest.<br><br>This includes environmental and lifestyle factors that may affect the relationship between the microbiome and the condition of interest. Participant diet and medication use should be summarized, if known.<br><br>At minimum, age and sex of all participants should be summarized.	Yes	Supplementary_Data.sheet 2.1 (variables) and 2.2 (codebook).
10.0	Microbiome data	Report descriptive findings for microbiome analyses with all applicable outcomes and covariates.	STORMS	This includes measures of diversity as well as relative abundances. These descriptive findings should be reported both for the sample overall and for individual groups.	Yes	Pages 11-14
10.1	Taxonomy	Identify taxonomy using standardized taxon classifications that are sufficient to uniquely identify taxa.	STORMS	If not using full taxonomic hierarchy, make sure it is clear whether names stated are species, genera, family, etc.	Yes	Pages 11-13

				Italicize genus/species pairs. Consult journal guidelines or standardized references on taxonomic nomenclature. For instance, https://wwwnc.cdc.gov/eid/page/scientific-nomenclature		
10.2	Differential abundance	Report results of differential abundance analysis by the variable of interest and (if applicable) by time, clearly indicating the direction of change and total number of taxa tested.	STORMS	If there are more than two groups, include omnibus (multigroup) test results if applicable to the research question.<br><br>If applicable, reported effect sizes should include a measure of uncertainty such as the confidence interval.	Yes	Pages 11-13
10.3	Other data types	Report other data analyzed--e.g. metabolic function, functional potential, MAG assembly, and RNAseq.	STORMS		Yes	Pages 5-11
10.4	Other statistical analysis	Report any statistical data analysis not covered above.	STORMS	This could include subgroup analysis, sensitivity analyses, and cluster analysis.<br><br>Visualizations should be easily interpretable and colorblind-friendly. The caption and/or main text should provide a detailed description of visualizations for visually-impaired readers.	Yes	Pages 11-15
**Discussion**						
11.0	Key results	Summarise key results with reference to study objectives	STROBE		Yes	Pages 15-16

12.0	Interpretation	Give a cautious overall interpretation of results considering objectives, limitations, multiplicity of analyses, results from similar studies, and other relevant evidence.	STROBE	Define or clarify any subjective terms such as "dominant," "dysbiosis," and similar words used in interpretation of results.<br><br>When interpreting the findings, consider how the interpretation of the findings may be summarized or quoted for the general public such as in press releases or news articles.<br><br>If causal language is used in the interpretation (such as "alters," "affects," "results in," "causes," or "impacts"), assumptions made for causal inference should be explicitly stated as part of 6.0 and 13.0.<br><br>Distinguish between function potential (ie inferred from metagenomics) and observed activity (ie metatranscriptomic, metabolomic, proteomic) if discussing microbial function.	Yes	Pages 15-17
13.0	Limitations	Discuss limitations of the study, taking into account sources of potential bias or imprecision.	STROBE	Also consider limitations resulting from the methods (especially novel methods), the study design, and the sample size.	Yes	Page 17
13.1	Bias	Discuss any potential for bias to influence study findings.	STORMS	May include sampling method, representativeness of study participants, or potential confounding.	Yes	Page 17

13.2	Generalizability	Discuss the generalisability (external validity) of the study results	STROBE	To what populations or other settings do you expect the conclusions to generalize?	Yes	Page 17
14.0	Ongoing/future work	Describe potential future research or ongoing research based on the study's findings.	STORMS		Yes	Page 17
**Other information**						
15.0	Funding	Give the source of funding and the role of the funders for the present study and, if applicable, for the original study on which the present article is based	STROBE		Yes	Page 18
15.1	Acknowledgements	Include acknowledgements of those who contributed to the research but did not meet criteria for authorship.	STORMS	For general guidelines on authorship, see http://www.icmje.org and https://www.elsevier.com/authors/journal-authors/policies-and-ethics/credit-author-statement	Yes	Page18
15.2	Conflicts of Interest	Include a conflicts of interest statement.	STORMS		Yes	Page 20
16.0	Supplements	Indicate where supplements may be accessed and what materials they contain.	STORMS		Yes	See Data Availability Statement, Page 56
17.0	Supplementary data	Provide supplementary data files of results with for all taxa and all outcome variables analyzed. Indicate the taxonomic level of all taxa.	STORMS	Depending on the analysis performed, examples of the supplemental results included could be mean relative abundance, differential abundance, raw p-value, multiple hypothesis testing-adjusted p-values, and standard error.	Yes	Supplementary Tables 5,6,7,10, and 11

				All discussed taxa should include the taxonomic level (e.g., class, order, genus).		
