# Supplementary material for: An integrated tumor, immune and microbiome atlas of colon cancer
Source: Nat Med. 2023 May 19;29(5):1273–86. doi: 10.1038/s41591-023-02324-5 (PMC10202816; doi:10.1038/s41591-023-02324-5)
Supplement: Supplementary file 2 — Reporting Summary [file 41591_2023_2324_MOESM2_ESM.pdf]

nature portfolio

Corresponding author(s): Davide Bedognetti and Wouter Hendrickx
NMED-RS117225

Last updated by author(s): Mar 26, 2023

Reporting Summary

Nature Portfolio wishes to improve the reproducibility of the work that we publish. This form provides structure for consistency and transparency in reporting. For further information on Nature Portfolio policies, see our Editorial Policies and the Editorial Policy Checklist.

Statistics

For all statistical analyses, confirm that the following items are present in the figure legend, table legend, main text, or Methods section.

n/a	Confirmed	
☐	☒	The exact sample size (*n*) for each experimental group/condition, given as a discrete number and unit of measurement
☐	☒	A statement on whether measurements were taken from distinct samples or whether the same sample was measured repeatedly
☐	☒	The statistical test(s) used AND whether they are one- or two-sided<br>*Only common tests should be described solely by name; describe more complex techniques in the Methods section.*
☐	☒	A description of all covariates tested
☐	☒	A description of any assumptions or corrections, such as tests of normality and adjustment for multiple comparisons
☐	☒	A full description of the statistical parameters including central tendency (e.g. means) or other basic estimates (e.g. regression coefficient) AND variation (e.g. standard deviation) or associated estimates of uncertainty (e.g. confidence intervals)
☐	☒	For null hypothesis testing, the test statistic (e.g. *F*, *t*, *r*) with confidence intervals, effect sizes, degrees of freedom and *P* value noted<br>*Give P values as exact values whenever suitable.*
☒	☐	For Bayesian analysis, information on the choice of priors and Markov chain Monte Carlo settings
☒	☐	For hierarchical and complex designs, identification of the appropriate level for tests and full reporting of outcomes
☐	☒	Estimates of effect sizes (e.g. Cohen's *d*, Pearson's *r*), indicating how they were calculated

Our web collection on statistics for biologists contains articles on many of the points above.

Software and code

Policy information about availability of computer code

Data collection	For AC-ICAM, clinical data was collected in excel from LUMC medical records. All samples were processed in the current study and all obtained (omics-) data is shared within the current resource manuscript (see data availability statement).

Data analysis

Tools for (pre-)processing of sequencing data included: FastQC (v.0.11.2), Flexbar (v3.0.3), Hisat2 (v2.1.0), SAMtools (v.1.3), (Bowtie2, v.2.3.4.2), subreads (v1.5.1), BWA (v.0.7.12),Bcl2fastq2 (v2.20) , Trimadap (v.0.1.3), Mutect (v.1.1.7), Strelka2 (bcbio-nextgen v1.1.1), VCFtoMAF (v.1.6.16), ConPair (Bergmann et al. 2016), RepeatFinder (Volfovsky et al. 2001), MANTIS (Kautto et al, 2017), OptiType (bcbio-nextgen v1.1.5), pVACtools (Hundel et al, 2020), MiXCR (v3.0.13), MetaPhlAn2. Downstream analyses were performed using R (v.3.5.1, or later). Transcriptome data analyses using R packages: EDASeq (v.2.12.0), preprocessCore (v.1.36.0), ConsensusClusterPlus (v.1.42.0), CMSclassifier (v.1.0), Rtsne (v.0.15), ConsensusTME (v.0.0.1.9), ESTIMATE (v.1.0.13), GSVA (v.1.38.2). Survival analysis using R package: survival (v.2.41–3), survminer (v.0.4.9), forestplot (v.1.7.2 & v2.0.1). WES data analysis using: R package maftools (v2.6.05), IGV (v2.11.0). Microbiome analysis was performed using: R package Phyloseq (v.1.34.0), R package vegan (v.2.5–6), python package SparCC3 (based on python3), R package NetCoMI (1.1.0), and visualized using Cytoscape (v3.9.1). Machine learning models were trained and tested using the R packages: glmnet (v4.1.4), doParallel (v1.0.17) to build glmnet models in parallel, factoextra (v1.0.7) and pracma (v2.3.8) for making PCA plots, survivalAnalysis (v0.3.0) for survival analysis, ggfortify (v0.4.14) for plotting results of ML models. Specific for TCGA data analysis: TCGAmutations (v 0.3.0), TCGAbiolinks (v2.18.0). Additional packages used for data formatting/manipulation included the following: stringr (v1.4.1), dplyr (v1.0.8), purrr (v0.3.4), data.table (1.14.2). R packages used for plotting and associated statistical analyses included: circlize (v.0.4.8), ComplexHeatmap (v.2.1.2), ggplot2 (v.3.3.2), ggpubr (v.0.4.0). and Ingenuity Pathway Analysis (IPA) software was used for core network analysis and visualization of the Global Molecular Network correlated with immunoSEQ productive TCR clonality. Analysis scripts and custom code can be found on the zenodo github release (DOI:10.5281/zenodo.7766220)

For manuscripts utilizing custom algorithms or software that are central to the research but not yet described in published literature, software must be made available to editors and reviewers. We strongly encourage code deposition in a community repository (e.g. GitHub). See the Nature Portfolio guidelines for submitting code & software for further information.

Data

Policy information about availability of data

All manuscripts must include a data availability statement. This statement should provide the following information, where applicable:

- Accession codes, unique identifiers, or web links for publicly available datasets
- A description of any restrictions on data availability
- For clinical datasets or third party data, please ensure that the statement adheres to our policy

Datasets used: Raw counts from RNASeq from TCGA were downloaded and processed using R package Biolinks (v.2.18.0). Somatic mutation calls from the TCGA MC3 Project were downloaded using R package TCGAmutations (v 0.3.0) using the function tcga_load() with parameters "COAD" for study and "MC3" for source. The microbiome genus relative abundance matrix for TCGA-COAD cohort (125 tumor samples) was downloaded from TCMA: The Cancer Microbiome Atlas (https://tcma.pratt.duke.edu).TCGA-COAD relative abundance matrix was filtered to exclude duplicated samples (samples from vial B, 8 samples).

Data Availability

BAM files for RNA and Whole Exome Sequencing data along with FastQ files for 16S rDNA sequencing and non-aligned WGS reads are made available through controlled access at dbGaP (phs002978.v1.p1) and public access SRA (PRJNA941834 (16S) & SUB12936752 (WGS)) . Names of the raw data files contain barcodes with a fixed structure:

-Study category: SER (Sidra Extrant Research)
-Study: SILU (Sidra-LUMC)
-Cancer type: CC (Colon Cancer)
-Patient ID: P001 (P for patient followed by 4-digit number)
-Sample: PT (primary tumor), AN (adjacent normal)
-Portion: 01, 02, 03 (in case of multiple PT from same patient)
-Assay + pipeline: A-01: RNASeq, GRCh38 (used for gene expression)
A-02: RNASeq, GRCh37 (used for MiXCR and neoantigen prediction)
B-02: WES, GRCh37
C-01: TCRSeq, Adaptive pipeline
D-01: 16S rRNA gene sequencing
D-02: WGS unaligned nonhost reads

Source Data for all main Figures, Extended Data Figures and Supplementary Figures 1-12 are available as "Supplementary Data". The "Supplementary Data" workbook includes per sample metrics from RNASeq, WES, TCR immunoSEQ, and microbiome profiling. A complete list of all the Source Data is available on Sheet 1 of the "Supplementary Data" workbook, followed by a Source Data Figure Location in Sheet 2.
A secondary repository for Supplementary Data is available via FigShare (DOI:10.6084/m9.figshare.16944775), including large files such as the Mutation Annotation Format (MAF) files for WES, segmentation file for the analysis of copy number genomic aberrations, the 16S Operational Taxonomic Unit (OTU) tables. FigShare will be also updated with metrics that will be generated in the future.
All processed data and clinical data are also available via cBioportal for interactive data exploration.
Access to SRA, cBioportal and Figshare is unrestricted and immediate, controlled access through dbGAP is managed by the NIH/NCI data access committee (DAC) through the dbGAP portal. For estimation of the required time to obtain access to the data, detailed statistics on the outcome and timeline of the data access request can be found here.

Human research participants

Policy information about studies involving human research participants and Sex and Gender in Research.

Reporting on sex and gender	Self-reported sex was not considered in the study design. The proportion of male and female (self-reported sex) is included in Supplementary Table 1 and Extended Data Figure 1. No sex-related analysis was performed as the identification of sex-specific immune/microbiome modulation was outside the scope of the present work.
Population characteristics	Extensive clinico-pathological and survival data of all 348 patients that were included in AC-ICAM are available (Supplementary Source Data). A summary of the population characteristics of is presented in Supplementary Table 1 and Supplementary Figure S3. These included age (range: 25-91 years; mean = 68 years, median= 69 years), self-reported sex (n = 182 males, n = 166 females), tumor stage (n = 55 Stage I, n = 122 Stage II, n = 110 Stage III, n = 61 Stage IV), tumor anatomic location (n = 183 right-sided colon, n = 165 left-sided colon), adjuvant treatment (n = 238 without treatment, n = 110 with adjuvant treatment), and history of cancer (n = 260 without history of cancer, n = 85 with history of cancer), among others.
Recruitment	Samples used in this research (tumor tissue and matched normal colon tissue, AC-ICAM cohort) are from colon cancer patients diagnosed at Leiden University Medical Center from 2001 to 2015 that did not object for future use of human tissues for scientific research and that were consented on biospecimen protocol "Immunology and Genetic of colon Cancer" approved by the Committee on Medical Ethics of Leiden University Medical Center (study protocol n. P00.193 (06/2001).
Ethics oversight	Samples used in this observational cohort study (tumor tissue and matched normal colon tissue, AC-ICAM cohort) are from colon cancer patients diagnosed at Leiden University Medical Center, the Netherlands, from 2001 to 2015 that did not object for future use of human tissues for scientific research and that were consented on biospecimen protocol "Immunology and Genetic of colon Cancer" approved by the Committee on Medical Ethics of Leiden University Medical Center (study protocol n. P00.193 (06/2001)). DNA and RNA from those samples were extracted at Leiden University Medical Center and then transferred to Sidra Medicine for sequencing together with de-identified clinico-pathological data of the corresponding patients (Sidra Medicine IRB study protocols n. 1768087-1 (04/2016) / 1602002725 (06/2022)). All genomic assays (i.e., WES, WGS, 16S RNA gene sequencing, RNA-seq, TCR sequencing, and PCR were performed at Sidra Medicine, Doha, Qatar). Patient information was de-identified and patient samples were anonymized and handled according to the medical guidelines described in the Code of Conduct for Proper Secondary Use of Human Tissue of The Federation of Dutch Medical Scientific Societies. This research was performed according to the recommendations outlined in the Helsinki Declaration.

Note that full information on the approval of the study protocol must also be provided in the manuscript.

Field-specific reporting

Please select the one below that is the best fit for your research. If you are not sure, read the appropriate sections before making your selection.

☒ Life sciences ☐ Behavioural & social sciences ☐ Ecological, evolutionary & environmental sciences

For a reference copy of the document with all sections, see nature.com/documents/nr-reporting-summary-flat.pdf

Life sciences study design

All studies must disclose on these points even when the disclosure is negative.

Sample size	Sample size calculation is challenging in multi-omics studies due to the multitude of parameters that could be examined (implying the use of different tests from different platforms generating data with different data distribution) and empirical methods have been used by many consortia. Correlation between ICR and survival was declared as primary objective in the research proposal submitted to the funding agency before any genomic data was generated, representing therefore a prospective-retrospective validation (JSREP07-010-3-005).<br>In the submitted proposal (2015), we planned to profile 400 tumors for gene expression analysis (samples from 456 patients were screened, samples from 391 patients were available for processing and samples from 348 patients retained after QC in the final cohort, see Extended Data Fig. 1), and at least 100 tumor-normal pairs for WES analysis (initially planned only for a subgroup of ICR high vs Low tumors), and 100 TCR sequencing using immunoSEQ assay considering the high amount of DNA that is necessary (> 2ug). Securing of additional funds allowed us to perform WGS and 16S rRNA sequencing, and to expand the WES and TCR analyses to any sample with sufficient DNA available. No specific power calculation was performed at that time and the targeted sample size was based on the estimated number of samples that could be retrieved from LUMC (n = 400), which compared favorably with the sample size of similar studies in the field. For instance the TCGA Colon and Rectal Cancer dataset available at that time had 276 patients (The Cancer Genome Atlas Network, Comprehensive molecular characterization of human colon and rectal cancer, Nature, volume 487, pages330–337 (2012)).<br>Regarding the detection of somatic mutations, and considering the overall somatic mutations frequency in colon cancer, 150 tumor exomes will give a power > 90% to detect a 10% mutational frequency in 90% of genes. (Spratt, D. E. et al. Racial/Ethnic Disparities in Genomic Sequencing. JAMA Oncol. 2, 1070–1074 (2016))<br>Regarding the survival analysis, in terms of ICR (primary objective in the submitted proposal), for the comparison between ICR High vs ICR Low, with 77 OS events detected, our study has a power > 80% for an HR of 0.5 with a two-sided α of 0.05. With 154 OS events in the whole cohort, our study has a power of 90% for an HR of 0.59 (assuming two group of equal size c), and a power of 90% for an HR of 0.57 (assuming groups with unequal sample size, 2:1) with a two-sided α of 0.05.

Data exclusions

The initial patient cohort for which samples were screened consisted of 456 patients. Specimen requirements included that the corresponding tumor anatomic site should be colon, the collected specimen included malignant tissue of the primary tumor, and the primary tumor is of epithelial origin. This resulted in the exclusion of 22 patients for which tumor anatomic site was not colon (i.e., rectum, jejunum, ilieum), 17 patients for which collected tissues were non-malignant (including carcinoma in-situ), 11 patients for which collected tissues were relapses or metastases of the primary tumor and 7 patients with a primary tumor of non-epithelial origin. Patients that received radiotherapy and/or chemotherapy prior to resection (n = 8). These exclusion criteria led to a total of primary colon tumors from 391 patients for sample processing. For thirty of these patients, insufficient material was available for DNA and RNA isolation. Sequencing was performed on 361 primary tumor samples. Following stringent quality control criteria, sequencing data of 13 patients were removed, which left a total of 348 patients in the AC-ICAM cohort. An overview of sample exclusions is presented in Extended Data Fig. 1.

Replication

The immunoSEQ assay, a dedicated assay for deep sequencing of the TRB gene, was applied to 114 tumors and 9 normal colon tissues. As second method, TCRB gene sequence information was also extracted from bulk RNA sequencing using the software MiXCR from data of 341 tumor samples. For samples that were profiled by both methodologies, TCR clonality derived from the immunoSEQ assay was correlated to the TCRB clonality derived from MiXCR using the Pearson correlation test (Fig. 2b).

The relationships between ICR and CMS depicted in Fig. 1 were confirmed in the TCGA colon cancer cohort (TCGA-COAD, Supplementary Fig. 2). Overall, in TCGA-COAD, the survival differences were attenuated (in the PFS analysis) or absent (in the OS analysis) for ICR, immune infiltrates, and CMS. Nevertheless, ICR still stratified survival in patients with CMS4 cancers (Supplementary Fig. 2, PFS analysis).

Microbiome genus relative abundance matrix for TCGA-COAD cohort estimated by WGS was downloaded from TCMA: The Cancer Microbiome Atlas (Dohlman et al). This dataset was used to confirm the presence of microbial genera in colon cancer. After applying the same abundance filter to AC-ICAM246 and TCGA-COAD datasets, AC-ICAM captured all the genera detected in TCGA-COAD. Furthermore, the co-abundance patterns of microbial genera were compared between cohorts (Supplementary Fig. 10).

An elastic net OS cox regression model was run on the AC-ICAM246 training set. Mean cross validation of the best model was used for optimization of hyperparameters using data of the AC-ICAM246 training set only. The resulting MBR classifier and corresponding calculated scores were strongly associated with survival in the AC-ICAM246 cohort (n = 246). Two independent testing cohorts were used to confirm the association between MBR and overall survival. These included an independent set of 42 samples of the AC-ICAM cohort (AC-ICAM42, testing set) that were reserved for internal validation, and 117 samples of the TCGA-COAD cohort as external dataset (TCGA-COAD, testing set), as well as the combined testing set (AC-ICAM42 + TCGA-COAD, n = 159). The concordance indexes of the final MBR model in both test sets were equal to those obtained through cross-validation of the best MBR model in the training set, suggesting a high generalizability of the model to new data.

The genus with the strongest effect in the MBR classifier was Ruminococcus 2. Using WGS data, we were able to identify the actual Ruminococcus species and demonstrated that Ruminococcus 2 reads mapped to Ruminococcus bromii (R. bromii). We further validated these findings with a third technique, R. bromii PCR. R. bromii presence was confirmed by PCR, which had strong correlation with sequencing data (i.e., 91% concordance between WGS and PCR) (Extended Data Figure 10).

We used data from TCGA-COAD as external validation cohort to test the mICRoScore (testing set). TCGA-COAD cohort includes 107 patients with both tumor microbiome data and RNASeq data available (used for ICR estimation). The survival between patients with mICRoScore High and mICRoScore Low was compared using a log-rank test.

Randomization

This is an observational cohort study. The study does not involve an intervention, so patients were not randomized. Samples biobanked at LUMC were used and processed according to sample availability. Initially we decided, for microbiome analysis, to only include patients for whom there was sufficient material to perform 16S RNA gene sequencing in both tumor and normal colon samples (246 patients, AC-ICAM246). This analysis was presented in the first version of the submitted manuscript.
During the review process a request was made to expand the number of samples analyzed for microbiome composition. We then analyzed tumor samples from 42 patients for whom there was no sufficient material from normal colon (ICAM42). Those samples were used to validate the MBR score that was developed in the 246 samples (AC-ICAM246).

Blinding

The study does not involve an intervention and did not compare treatments so there was no blinding.
Sample processing was performed by operators that did not have access to outcome data at that time.

Reporting for specific materials, systems and methods

We require information from authors about some types of materials, experimental systems and methods used in many studies. Here, indicate whether each material, system or method listed is relevant to your study. If you are not sure if a list item applies to your research, read the appropriate section before selecting a response.

Materials & experimental systems

n/a	Involved in the study
☒	☐ Antibodies
☒	☐ Eukaryotic cell lines
☒	☐ Palaeontology and archaeology
☒	☐ Animals and other organisms
☒	☐ Clinical data
☒	☐ Dual use research of concern

Methods

n/a	Involved in the study
☒	☐ ChIP-seq
☒	☐ Flow cytometry
☒	☐ MRI-based neuroimaging
