# Supplementary figures and images for: An integrated tumor, immune and microbiome atlas of colon cancer
Source: Nat Med. 2023 May 19;29(5):1273–86. doi: 10.1038/s41591-023-02324-5 (PMC10202816; doi:10.1038/s41591-023-02324-5)

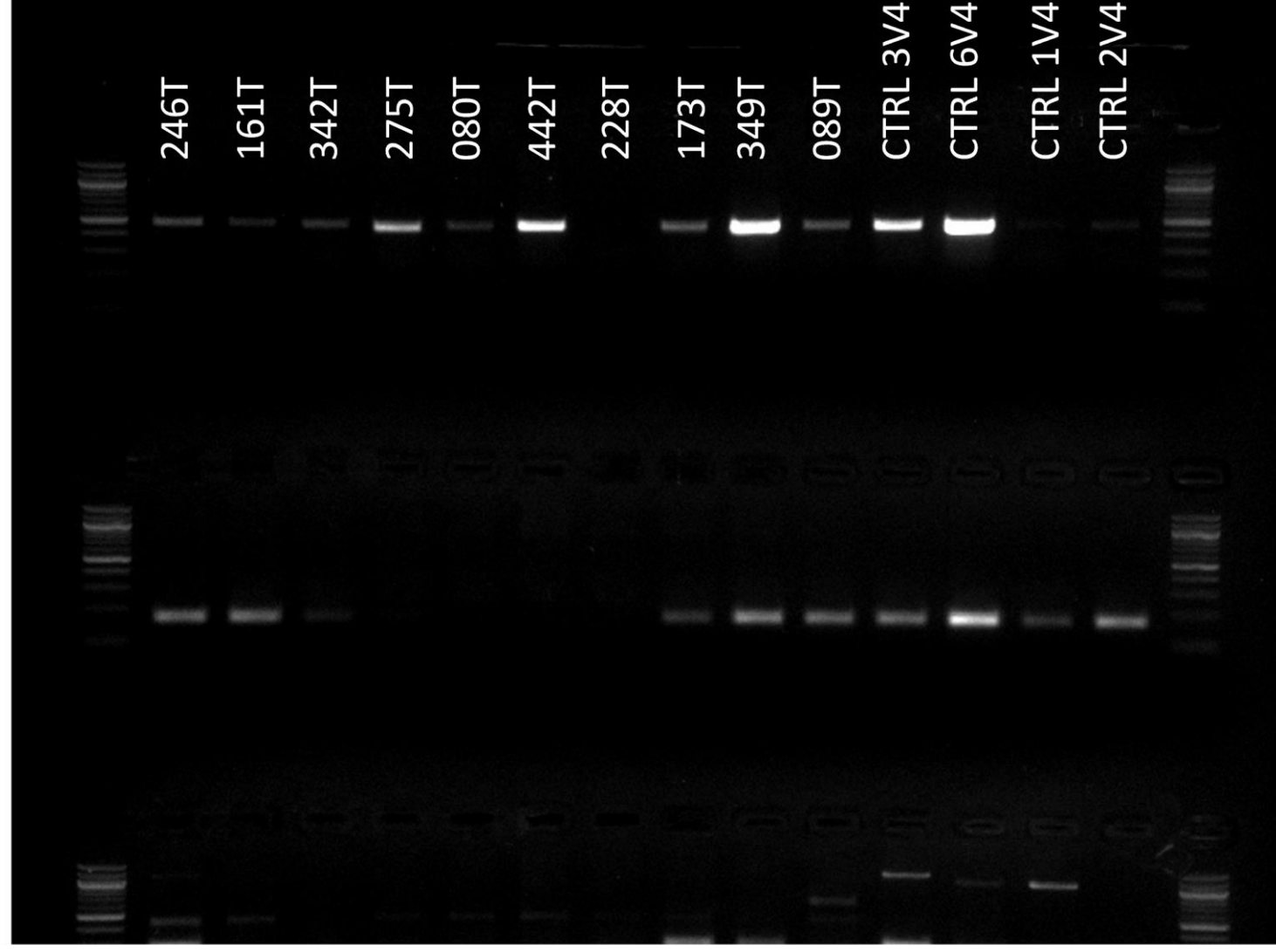

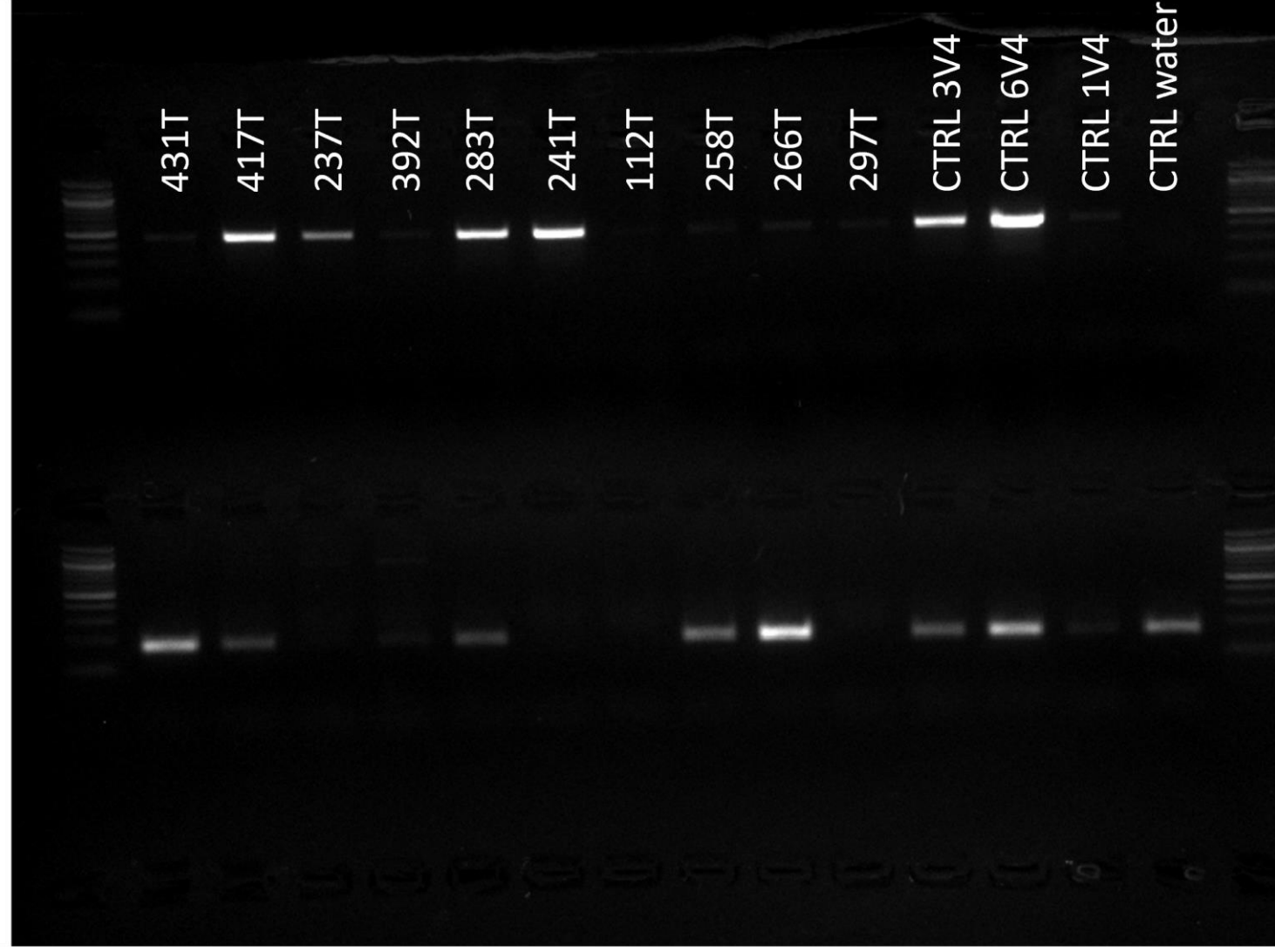

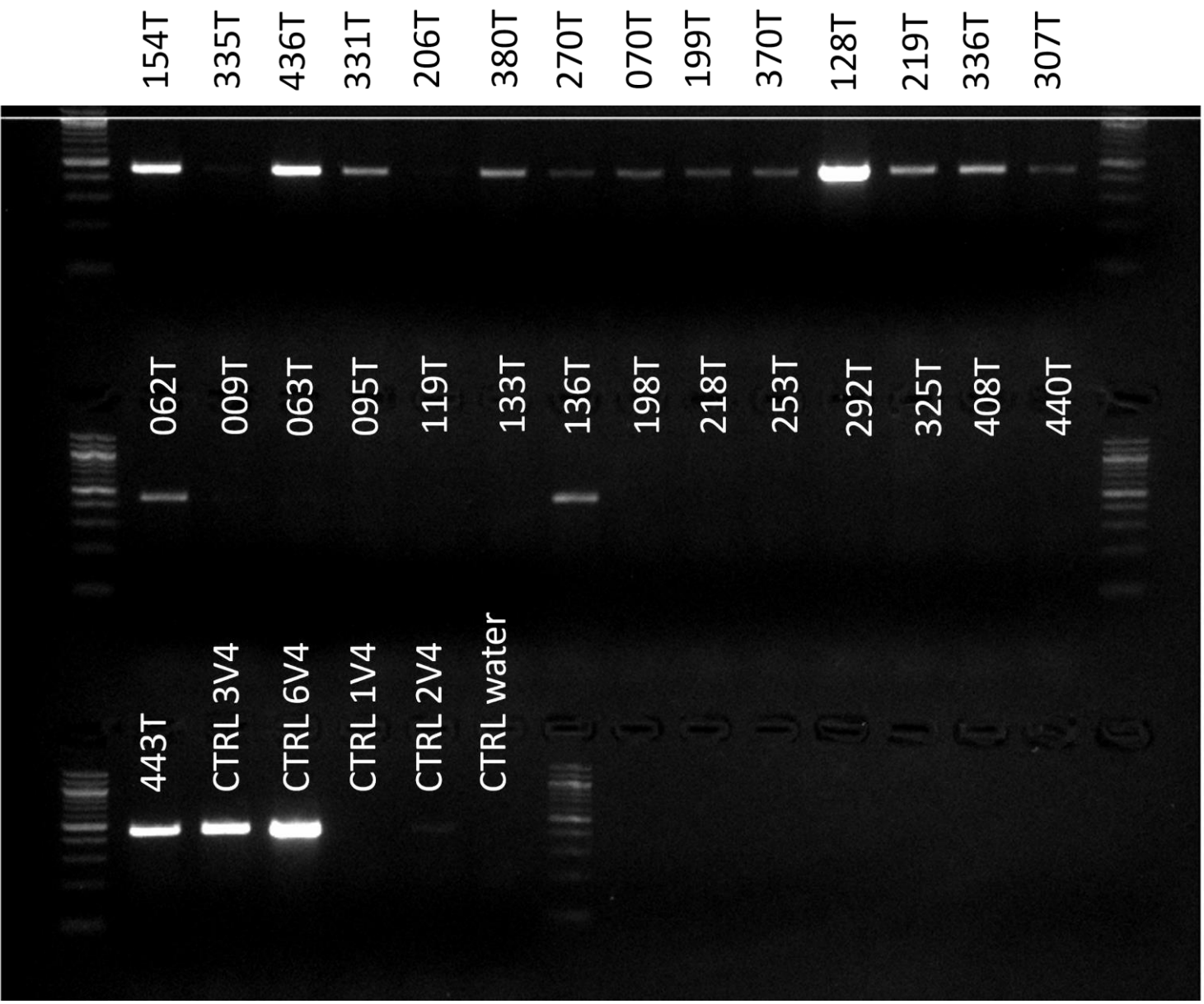

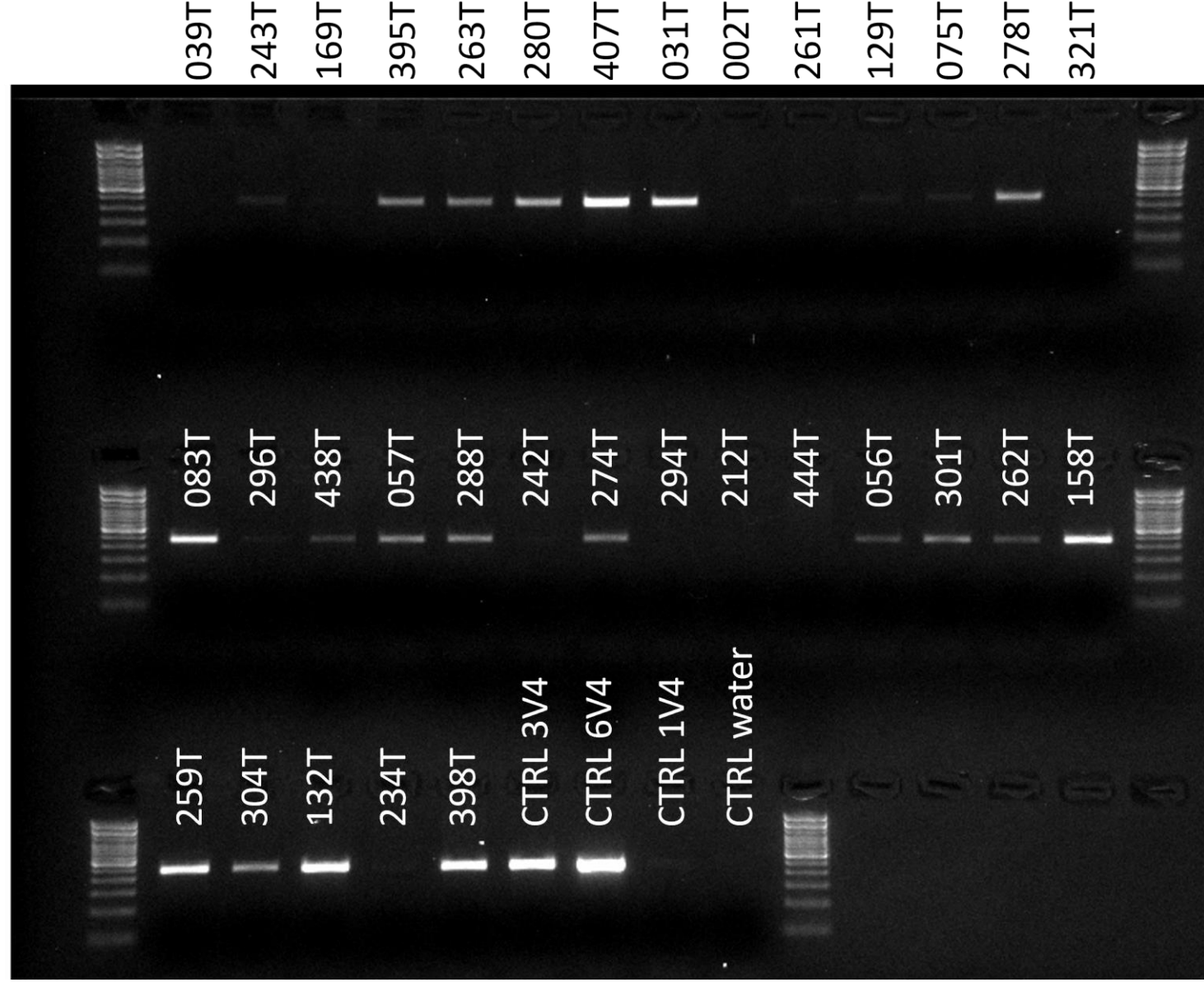

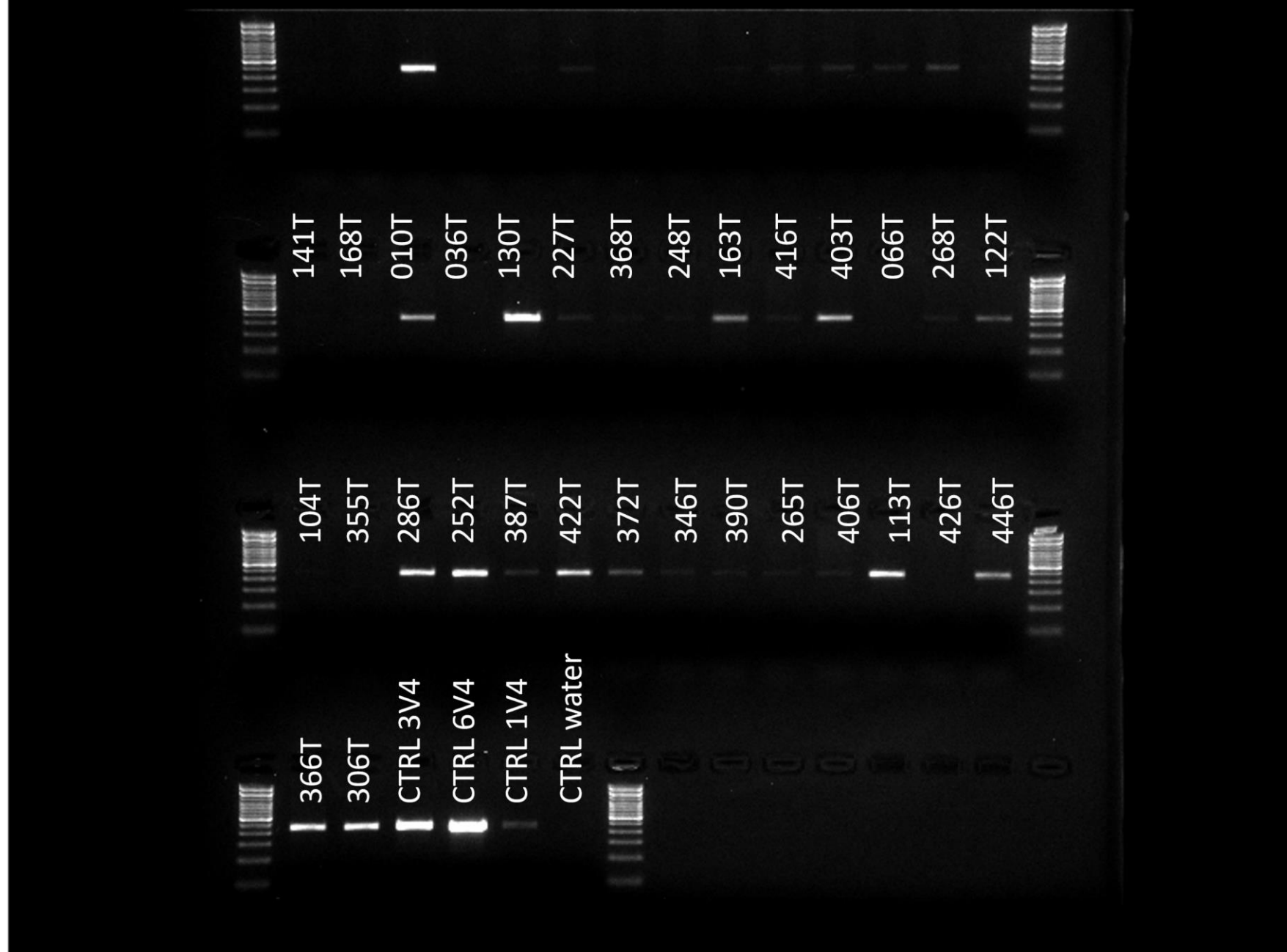

143T  
285T  
290T  
298T  
389T  
140T  
354T  
410T  
413T  
155T  
238T  
250T  
167T  
123T

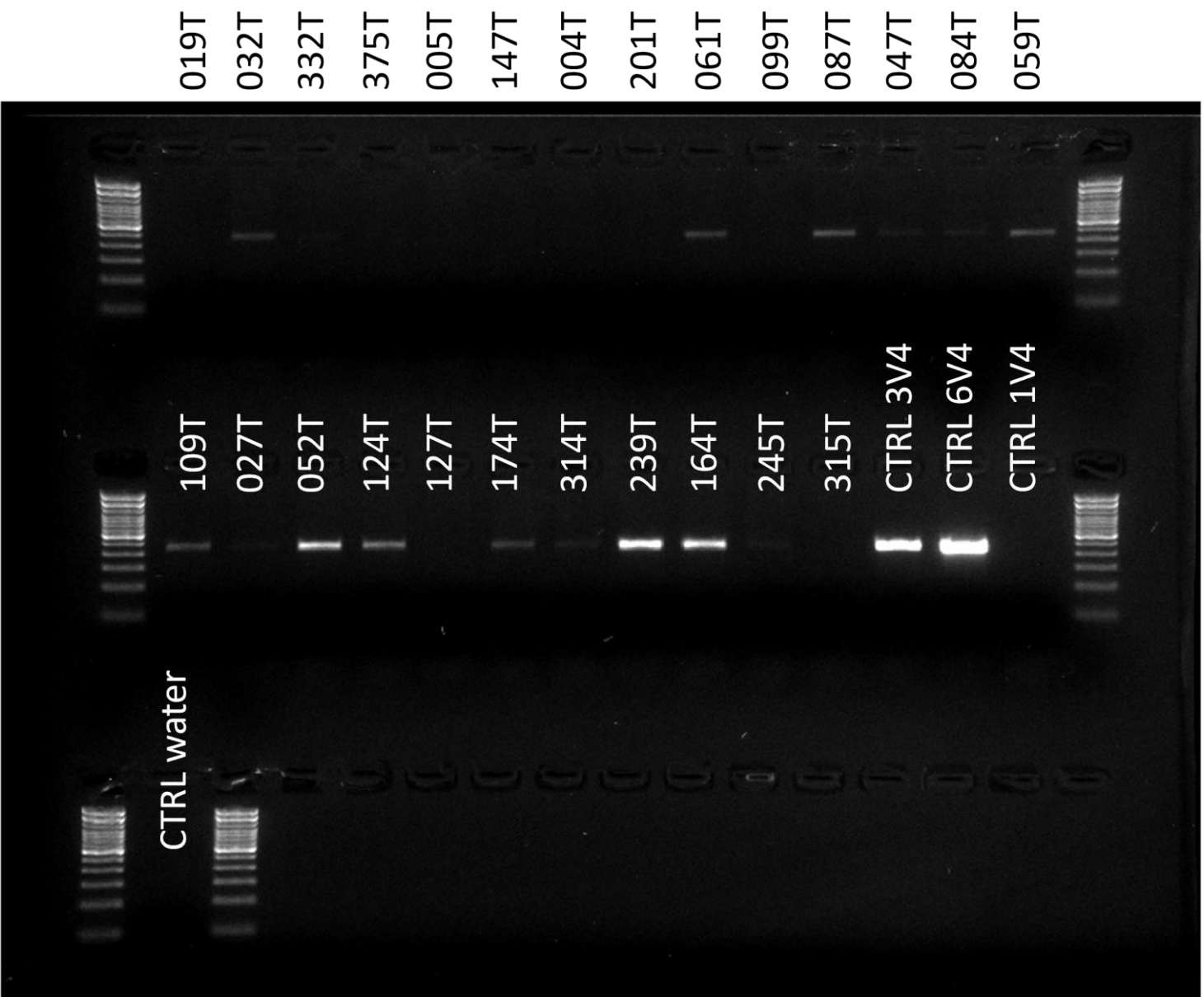

Supplement: Supplementary file 5 — PDF file of the raw PCR blots seen in Extended Data Fig.10b. [file 41591_2023_2324_MOESM5_ESM.pdf]
